# A new perspective on *Icriomastax* (Diptera: Tipulidae): phylogeny and description of five new species

**DOI:** 10.7717/peerj.21121

**Published:** 2026-04-16

**Authors:** Jéssica Gouvêa, Leonardo H. Gil-Azevedo

**Affiliations:** Departamento de Entomologia, Museu Nacional, Universidade Federal do Rio de Janeiro, Rio de Janeiro, RJ, Brazil

**Keywords:** Tipuloidea, Neotropical region, Systematics, Morphology, Crane fly

## Abstract

*Icriomastax* Enderlein, 1912, **stat rev.** historically treated as a subgenus of *Ischnotoma* Skuse, 1890, has remained taxonomically neglected since the description of its last species in 1945. Here, we provide a comprehensive phylogenetic analysis of the group, based on a matrix with 153 morphological characters across 61 taxa. Parsimony analyses under equal and implied weighting consistently recovered *Icriomastax* as a monophyletic group, supporting its elevation to full genus status. In contrast, *Ischnotoma* and *Holorusia* Loew, 1863 were recovered as paraphyletic, and the position of *Is*. (*Neotipula*) remained unstable, reinforcing the need for a broader review of *Ischnotoma sensu lato*. Additionally, we describe five new species: *Icriomastax catia* sp. nov.,* Icriomastax coscaroni* sp. nov., *Icriomastax craigi* sp. nov.,* Icriomastax lopesae* sp. nov., and* Icriomastax monnei* sp. nov. These results provide a new and consistent taxonomic framework and highlight the relevance of morphology-based systematics for underexplored lineages in Tipulidae. Our results raise questions about Southern Hemispheric diversification patterns in Tipulidae and offer a basis for future integrative studies combining morphological and molecular data.

## Introduction

Tipulidae is one of the most diverse families of Diptera, with over 4,300 described species worldwide (Oosterbroek, 2025). Despite its diversity, taxonomic studies focusing on South American species remain limited. Most recent studies in the last 25 years have focused primarily on fossils (*e.g*., Ribeiro, Reis Santos & Santos, 2021). In broader phylogenetic analyses of the family, South American species are rarely included (*e.g*., Lukashevicha & Ribeiro, 2019; Song et al., 2023), resulting in a significant gap in the understanding of the evolutionary history and classification of Neotropical taxa.

*Icriomastax* Enderlein, 1912 is a Neotropical genus traditionally treated as a subgenus of *Ischnotoma* Skuse, 1890. Vane-Wright (1967) suggests a close relationship between *Ischnotoma* (including *Icriomastax*), *Zelandotipula* Alexander, 1922, and *Holorusia* Loew, 1863 based on morphological similarities. However, these insights were not tested with phylogenetic methods, relying solely on comparative morphology. Since then, no study has formally tested the relationship between *Icriomastax* and other related genera, leaving its phylogenetic placement uncertain.

*Ischnotoma* currently comprises three subgenera: *Is.* (*Icriomastax*), with ten species distributed in Brazil and north Argentina; *Is.* (*Ischnotoma*) with 40 species occurring in Australia and southern South America (Argentina and Chile); and *Is.* (*Neotipula*) Alexander, 1940 with four species ranging from Peru to Guatemala (Oosterbroek, 2025). A recent geometric morphometric study suggested that *Is.* (*Neotipula*) should be considered a separate genus (Gouvêa & Gil-Azevedo, 2022). Furthermore, the fossil record for the group is extremely scarce, with the only known fossil being *Ischnotoma vasifera* (Cockerell & Haines, 1921) from Oligocene deposits in the Isle of Wight.

Here, we describe five new species, and transfer a species previously assigned to *Zelandotipula* to *Icriomastax*. We also perform a comprehensive phylogenetic analysis to test the monophyly of *Icriomastax sensu* Alexander & Alexander (1970) and to clarify its relationship with other Tipulidae genera. Our study provides a revised framework for future taxonomic and evolutionary studies for this group.

## Materials & Methods

### Taxon sampling

The ingroup comprises all 15 species of *Icriomastax,* comprising the ten species previously described, and five new species described herein. To establish a robust phylogenetic framework and test the monophyly of the genus, we selected a diverse outgroup composed of *Ischnotoma*, four of *Is*. (*Neotipula*), seven of *Holorusia*, and six of *Zelandotipula*, all previously proposed as related genera (Vane-Wright, 1967). The tree root was defined using *Tipula* (*Tipula*) *oleracea* Linnaeus, 1758, the type species of *Tipula*, allowing for appropriate polarization of character states. The fossil species *Is. vasifera* was not included in the analysis due to the absence of codifiable morphological characters compatible with the dataset.

### Examined material

Specimens were examined from the following collections (acronyms in parentheses):

Academy of Natural Sciences of Drexel University, Philadelphia, USA (ANSP); California Academy of Sciences, San Francisco, USA (CAS);

Canadian National Collection of Insects, Arachnids and Nematodes, Ottawa, Canada (CNC);

Carnegie Museum of Natural History, Pittsburgh, USA (CMNH);

Instituto Nacional de Pesquisas da Amazônia, Manaus, Amazonas, Brazil (INPA); KwaZulu-Natal Museum, Pietermaritzburg, South Africa (KZN);

Museu de Zoologia, Universidade de São Paulo, São Paulo, Brazil (MZSP);

Museu Nacional, Universidade Federal do Rio de Janeiro, Rio de Janeiro, Brazil (MNRJ);

Museu Paraense Emílio Goeldi, Belém, Brazil (MPEG); National Museum of Natural History, Smithsonian Institute, Washington, USA (NMNH);

National Museum of Victoria, Melbourne, Australia (NMV);

Natural History Museum, London, UK (BMNH);

Naturalis Biodiversity Center, Leiden, Netherlands (NCB);

Tasmanian Museum and Art Gallery, Hobart, Australia (TMAG);

Universidade Federal do ABC, Santo André, Brazil (UFABC);

Zoologisches Forschungsmuseum Alexander Koenig, Bonn, Germany (ZFMK).

### Morphological characters: construction and terminology

Characters were derived from direct examination of adult morphology, supported by previous works (*e.g.*, Alexander, 1936; Alexander, 1942; Vane-Wright, 1967). Primary homology hypotheses were formulated following De Pinna (1991), and the logical construction of characters followed Sereno (2007) and Fitzhugh (2006). Characters were preferentially coded reductively (Wilkinson, 1995) and treated as unordered (Fitch, 1971). When multiple states occurred in the same terminal taxon, they were treated as polymorphic. We codified those hypotheses in a data matrix using the program Mesquite (Maddison & Maddison, 2017). Terminology of the external morphology of the adults follows Cumming & Wood (2017), wings follow De Jong (2017), and the gonostyle of the male genitalia follows Ribeiro (2006).

### Phylogenetic analyses

Parsimony analyses were conducted under both equal weights (EW) and implied weights (IW) approaches. In IW, characters were down-weighted based on their degree of homoplasy (Goloboff, 1993). We followed the rationale of Goloboff et al. (2008), who demonstrated that weighting against homoplasy increases the stability of clades when novel characters or taxa are added. Although some authors argue that weighting introduces subjectivity, equal weighting also makes implicit assumptions namely, that all characters are equally reliable (Wheeler, 1995; Goloboff et al., 2008).

To explore topological stability across weighting schemes, we tested 11 values of the concavity constant *k* (Goloboff et al., 2008; Mirande, 2009; Gil-Azevedo & Coscarón, 2020). Analyses were run in TNT version 1.5 (Goloboff & Catalano, 2016), using 5,000 random addition sequence (RAS) replicates, saving 500 trees per replicate under tree-bisection-reconnection (TBR) swapping.

Clade stability was assessed through parameter sensitivity analysis (PSA), represented graphically using the “Navajo rugs” (Wheeler, 1995; Gil-Azevedo & Coscarón, 2020), in which black squares indicate that a clade is recovered under the given parameter. According to Whiting et al. (1997), greater confidence can be placed in clades supported across a wide range of analytical conditions. Support values were estimated through bootstrap resampling using Poisson independent reweighting, with GC frequency differences (Goloboff et al., 2003), in 5,000 replicates with RAS + TBR.

The electronic version of this article in Portable Document Format (PDF) will represent a published work according to the International Commission on Zoological Nomenclature (ICZN), and hence the new names contained in the electronic version are effectively published under that Code from the electronic edition alone. This published work and the nomenclatural acts it contains have been registered in ZooBank, the online registration system for the ICZN. The ZooBank LSIDs (Life Science Identifiers) can be resolved and the associated information viewed through any standard web browser by appending the LSID to the prefix http://zoobank.org/. The LSID for this publication is: (urn:lsid:zoobank.org:pub:9342C62F-53CF-4A99-9247-E6EDFFBE429A). The online version of this work is archived and available from the following digital repositories: PeerJ, PubMed Central SCIE and CLOCKSS.

## Results

The data matrix included 61 terminal taxa and 153 characters derived from adult male and female specimens (Appendix 1). The list of characters and character states is provided in Appendix 2 and all terminal taxa in Appendix 3.

The parsimony analysis under equal weights (EW) yielded in 12 most parsimonious trees (MPTs), with a tree length of 993 steps, a consistency index (CI) of 0.15, and a retention index (RI) of 0.54. Under implied weighting (IW), 11 values of the concavity constant *k* were tested, corresponding to average character fit of 50%, 54%, 58%, 62%, 66%, 70%, 74%, 78%, 82%, 86%, and 90% (Goloboff et al., 2008; Mirande, 2009). Each IW analysis produced a single MPT (Table 1).

**Figure 1:**
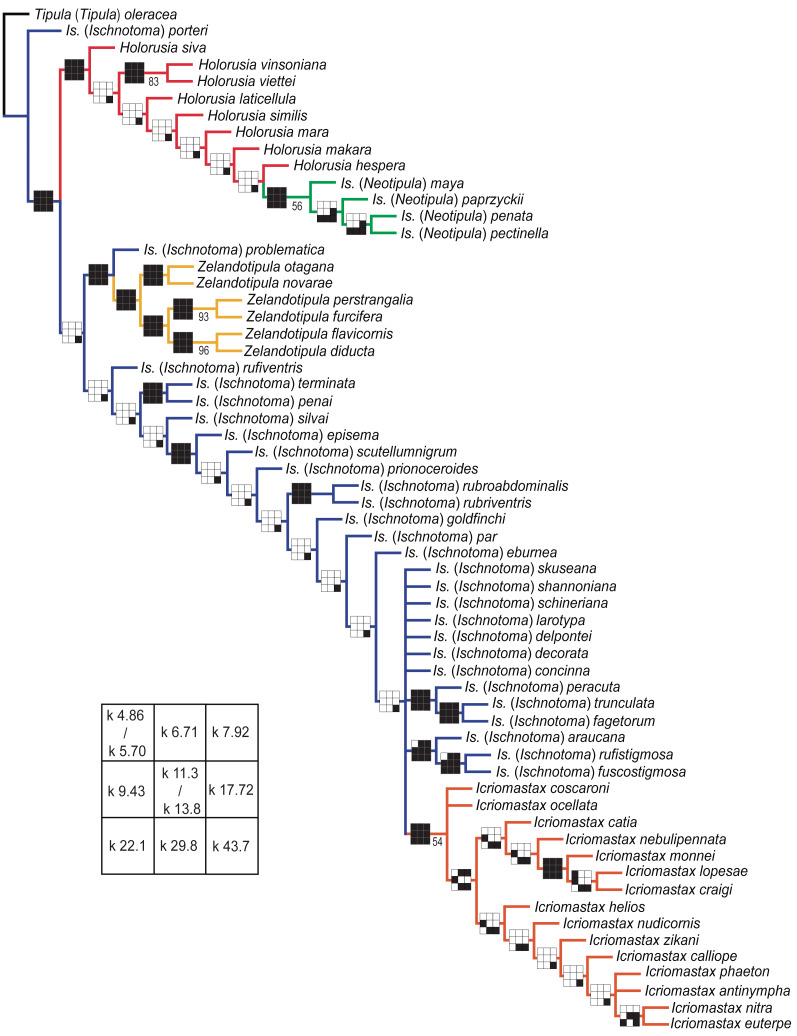
Most parsimonious trees topologies across implied weighting k-values. Summary of scores and topological differences among the Most Parsimonious Trees (MPT) obtained by Implied Weighting (IW) analyses, under concavity constant (k) calculated with average character fits (k-value) of 50, 54, 58, 62, 66, 70, 74, 78, 82, 86, and 90%.

To assess topological stability across parameter sets, we used the strict consensus tree obtained under EW as a reference framework to plot parameter sensitivity analysis (PSA) and bootstrap values (Fig. 1). Although IW is our preferred criterion for evaluating relationships, the EW consensus tree provided a more inclusive topology for graphical comparisons, allowing for direct visualization of support and variation across analytical conditions.

All analyses recovered *Ischnotoma* and *Is*. (*Ischnotoma*) as paraphyletic groups. *Holorusia* and *Is*. (*Neotipula*) formed a strongly supported clade, although *Holorusia* appeared as paraphyletic in all topologies, while *Is*. (*Neotipula*) was always monophyletic. *Zelandotipula* was recovered as a monophyletic genus in all analyses, with the Australian species consistently placed as sister to the clade formed by the Neotropical *Zelandotipula* species. The monophyly of *Icriomastax* was recovered across all topologies.

## Discussion

All phylogenetic analyses consistently recovered *Ischnotoma* as paraphyletic, reinforcing long-standing doubts about the monophyly of the genus. From this point forward, we refer to *Ischnotoma* only in the sense of the nominal subgenus *Is.* (*Ischnotoma*), which itself was also recovered as paraphyletic. This result is consistent with the observed morphological heterogeneity among its species, such as variation in antennae, wing venation and pigmentation, and male terminalia. These differences point to the need for a thorough revision of the group, including all 35 valid species. Such a revision will likely require division into smaller, morphologically coherent genera. Similar taxonomic instability has been reported in other crane fly lineages undergoing reassessment with expanded sampling and phylogenetic methods (Petersen et al., 2025).

**Table 1:** Most Parsimonious Trees topologies across implied weighting k-values. Summary of scores and topological differences among the Most Parsimonious Trees (MPT) obtained by Implied Weighting (IW) analyses, under concavity constant (k) calculated with average character fits (k-value) of 50, 54, 58, 62, 66, 70, 74, 78, 82, 86, and 90%.

*k* value	Distref	Kref	Lenght	MPT	*Fit*	Defspr	Agree	Nodcons
k0	50	4.860	906	1	59.611	0	0	0
k1	54	5.705	906	1	55.524	0	61	59
k2	58	6.711	891	1	51.356	14	39	39
k3	62	7.929	890	1	47.093	1	55	55
k4	66	9.434	887	1	42.758	4	53	50
k5	70	11.340	886	1	38.325	6	54	49
k6	74	13.832	886	1	33.784	0	60	58
k7	78	17.231	885	1	29.118	2	57	55
k8	82	22.140	884	1	24.300	1	57	57
k9	86	29.854	882	1	19.307	7	50	45
k10	90	43.740	879	1	14.105	12	38	31

The close relationship between *I*. (*Neotipula*) and *Holorusia* observed in our analyses, corroborates previous suggestions based on wing morphometric studies (Gouvêa & Gil-Azevedo, 2022). These four *I*. (*Neotipula*) species share several morphological characters with *Holorusia*, such as the absence of a vertical tubercle on the vertex (character 2, state 0), serrated antennae (character 13, state 1), unmarked wings (characters 57-74), and parallel R_4_ and R_5_ veins (character 80, state 0). Together, these traits support their close affinity. Although these *I*. (*Neotipula*) species could potentially be included within *Holorusia*, the unique configuration of the male genitalia, especially sternite VIII, indicates a distinct lineage. This supports their recognition as a separate genus.

Our results strongly support elevating *Icriomastax* to full genus rank, distinct from *Ischnotoma*. Its monophyly was consistently recovered in all analyses, provided *Ic. nebulipennata* (Alexander, 1980) **comb. nov.** is included. This species, previously placed in *Zelandotipula*, lacks any diagnostic characters justifying that placement and is instead morphologically and phylogenetically congruent with *Icriomastax*. The clade is stable, as it is recovered in all k-value analyses, and well supported, with bootstrap values above 50%, which fall within the expected range for clades supported exclusively by morphological data. Morphology-based analyses typically yield lower statistical support relative to molecular datasets due to lower character sampling, higher levels of homoplasy, and the inherent constraints of discrete morphological coding. Therefore, the recovered support value is consistent with analytical expectations and does not undermine the validity of the clade. On the other hand, only one internal branch of *Icriomastax* can be considered stable, but without support. For this reason, we prefer not to discuss the internal organization of the genus.

The monophyly of *Icriomastax* is supported by a unique combination of synapomorphies. These include the presence of a stripe on the lateral side of the rostrum (character 8, state 1); filiform antenna (character 13, state 0); pleura with a stripe from the cervical sclerites to the base of the wing (character 28, state 1) and a spot between the prescutum and anepisternum (character 29, state 1); cell C hyaline (character 53, state 0); stigma without a whitened streak (character 54, state 0); wings with spots on almost every cell (character 57-74); tergite IX produced outwardly (character 101, state 0) and the presence of projections on sternite VIII (character 143, state 1). These characters are stable across all included species and form a robust morphological basis for genus-level recognition.

While species of *Ischnotoma* sensu stricto present a broad range of morphological states, such as spotted or entirely hyaline wings; filiform to serrate antennae; and high diversity in male genitalia structures, the diagnostic characters seen in *Icriomastax* are unique, stable and non-overlapping. This morphological consistency contrasts with the heterogeneous condition observed in *Ischnotoma*, in which states considered distinctive for *Icriomastax* are either absent or occur only as isolated, non-synapomorphic conditions. This level of discontinuity supports recognition of *Icriomastax* as a distinct genus rather than as a subgenus within *Ischnotoma*.

Although 27 of the 35 extant species were included in our analysis, a full resolution of relationships within *Ischnotoma* was beyond the scope of the present study. In this work, *Ischnotoma* is employed primarily as an outgroup to polarize characters and evaluate the monophyly of *Icriomastax*, which is the focal lineage of our analysis.

These results reinforce the relevance of morphology-based phylogenetics, especially when supported by rigorous character selection and matrix construction, particularly in groups with limited or no molecular data available. In addition, our findings raise broader questions about the biogeographic history of Neotropical Tipulidae. The disjunction between southern South America, New Zealand and Australia lineages, evident in both *Zelandotipula* and *Ischnotoma*, reflect the South Pacific Track patterns. Similar patterns have been reported in other Tipuloidea lineages (Ribeiro & Eterovic, 2011). The close relationship between *Holorusia* and *Is*. (*Neotipula*) further complicates traditional zoogeographic boundaries within Tipulidae and invites future biogeographical analyses integrating fossil records and molecular data.

Our approach aligns with recent calls to broaden systematic frameworks in Tipuloidea, like Petersen et al. (2025), who emphasize the need to overcome long-standing taxonomic bottlenecks through expanded character sampling and integrative methodologies. By reassessing morphological diversity and proposing a revised classification for *Icriomastax*, this study contributes to a more coherent and testable understanding of Tipulidae systematics.

Nonetheless, the absence of molecular data for *Icriomastax* and its putative sister groups remains a major limitation. Future research should aim to test the hypotheses presented here using genomic-scale data and integrative analytical frameworks. In addition, the paraphyletic status of *Ischnotoma* and *Holorusia*, consistently recovered across analyses, reinforces the need for comprehensive systematic revisions of these groups. Complementary morphological and molecular datasets will be essential for reconstructing the evolutionary history of Tipulidae in the Neotropics and to refine the classification of its diverse lineages.

### Taxonomy

**Table:** 

*Icriomastax* Enderlein, 1912 ** stat. rev.**
*Icriomastax*: Enderlein (1912: 9).

*Ischnotoma* (*Icriomastax*): Vane-Wright (1967: 515, 518, 521–524, 527, 530, 538, 540, 541, 546 figs. 8, 11). Alexander & Alexander (1970: 2, 8). Gouvêa & Gil-Azevedo (2017: 198-203; figs. 1–4). Gouvêa & Gil-Azevedo (2022: 1-20).

**Type** **species**: *Icriomastax ocellata* Enderlein, 1912

Other **species**: *Icriomastax antinympha* (Alexander, 1942) **comb. nov.;** *Icriomastax calliope* (Alexander, 1945) **comb. nov.**; *Icriomastax catia* **sp. nov.**; *Icriomastax coscaroni* **sp. nov.**; *Icriomastax craigi* **sp. nov.**; *Icriomastax euterpe* (Alexander, 1945) **comb. nov.**; *Icriomastax helios* (Alexander, 1949) **comb. nov.**; *Icriomastax lopesae* **sp. nov.**; *Icriomastax monnei* **sp. nov.**; *Icriomastax nebulipennata* (Alexander, 1980) **comb. nov.**; *Icriomastax nitra* (Alexander, 1945) **comb. nov.**; *Icriomastax nudicornis* (Macquart, 1838) **comb. nov.**; *Icriomastax phaeton* (Alexander, 1945) **comb. nov.**; *Icriomastax zikani* (Alexander, 1936) **comb. nov.**

**Diagnosis**: Nasus present; antennae filiform with 12-segmented and a few short verticils in the basal segments; vertex with vertical tubercle; wings moderate to heavily stained where all the stains are confined within the cells limits and never go over the veins; vein R_4_ curved upwards near wing margin; veins R_4_ and R_5_ smoothly curved towards each other near mid-length; Rs nearly as long as crossvein m-cu; axillary area moderately developed; alar squama bare. Male terminalia with tergite IX with posterior margin produced outwardly; lobe of gonostylus usually with wider apex and a short expansion on posterodorsal margin and in some species with short triangular to long and narrow black spines; sternite VIII with two projections on posterior margin; the female genitalia appear fundamentally the same in the group.

**Remarks:** In comparison to the closely related genera *Holorusia*, *Ischnotoma*, and *Zelandotipula*, *Icriomastax* is easily recognized by the wing spots, which can be either dark and distinct or more subtle, unlike *Holorusia* and Is. (*Neotipula*), which generally lack spots (except for the species *H. calliergon* (Alexander, 1940), *H. carmichaeli* (Brunetti, 1913), and with a spots placement entirely different from that found in some species of *Ischnotoma*, where most of the spots are located near the margin between veins R_3_ and M_2_ (*e.g*., *Is. delpontei* (Alexander, 1929), *Is. eburnea* (Walker, 1848), *Is. peracuta* Alexander, 1971, *Is. schneriana* (Alexander, 1928), *Is. shanonniana* (Alexander, 1929), *Is. skuseana* Alexander, 1928, *Is. trunculata* (Alexander, 1962)), and from that found in *Zelandotipula*, where the most common pattern is a single spot at the origin of the Rs vein, although in some species, there are widely distributed spots in the upper region of the wing, following the C vein and some in the br and bm cells (*e.g*., *Z. acutistyla* (Alexander, 1946), *Z. associans* (Walker, 1861), *Z. cristifera* (Alexander, 1946), *Z. daedalus* Alexander, 1976, *Z. horni* (Alexander, 1926)). In addition to the wing spots, the male genitalia are completely different from any other related genus, as the tergite IX and the gonostylus have unique shapes and structures compared to other groups.

Distribution and biology: *Icriomastax* has a Neotropical distribution, with records from northern Argentina, southern, and southeastern Brazil. They can be found from sea level up to 2,650 m. Unfortunately, only adult individuals have been studied, so no associations can be made regarding the biology of the immature stages.

We also described five new species of *Icriomastax*:

**Table:** 

**Figure 2:**
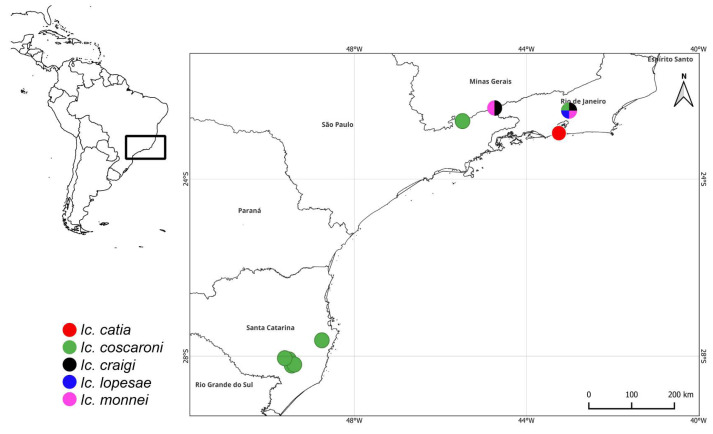
Geographic distribution map of *Icriomastax* new species. Circles colors represent the distribution of the new species: red, *Icriomastax catia*; green, *Ic. coscaroni*; black, *Ic. craigi*; blue, *Ic. lopesae*, pink, *Ic. monnei*. Multi-colored sectorized circles indicate localities where two or more species occur together.

**Figure 3:**
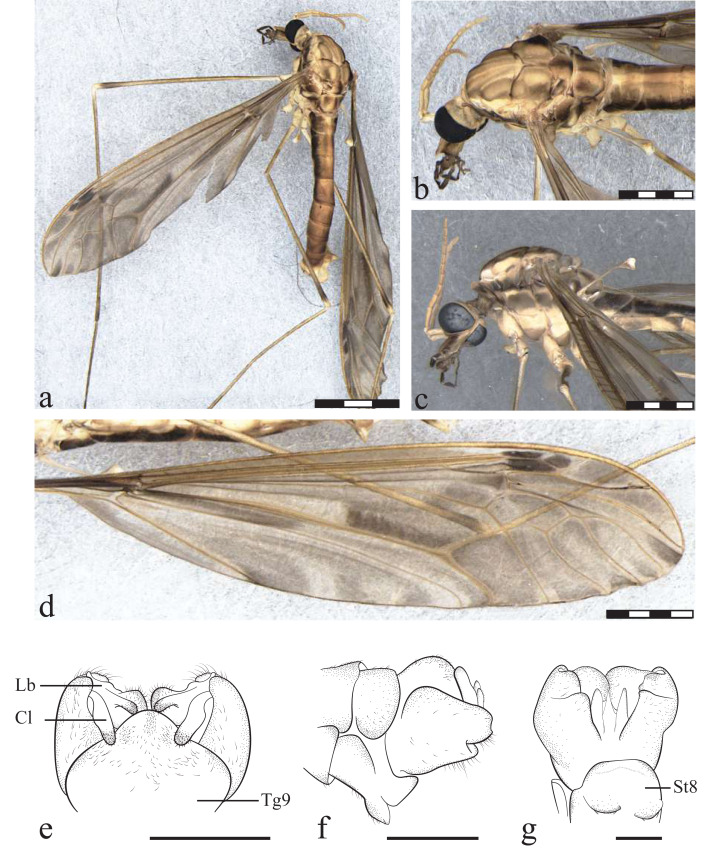
*Icriomastax catia* sp. nov. Holotype. Male (MNRJ31,130). (A) Habitus, dorsal view. Scale bar two mm. (B) Thorax, dorsal view. (C) Thorax, lateral view. (D) Wing. (E) Genitalia, dorsal view. (F) Genitalia, lateral view. (G) Genitalia, ventral view. Scale bar one mm. Abbreviations: Cl, clasper of gonostylus; Lb, lobe of gonostylus; St8, sternite VIII; Tg9, Tergite IX.

**Figure 4:**
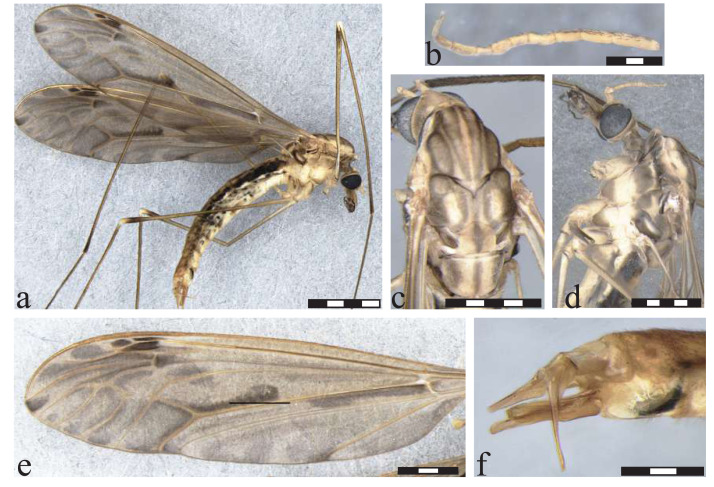
*Icriomastax catia* sp. nov. Paratype. Female (MNRJ31,743). (A) Habitus, lateral view. Scale bar two mm. (B) Antenna. Scale bar 250 µm. (C) Thorax, dorsal view. (D) Thorax, lateral view. (E) Wing. Scale bar one mm. (F) Genitalia, lateral view. Scale bar 500 µm.

*Icriomastax catia***sp. nov.**
urn:lsid:zoobank.org:act:7B009A34-D84B-45CF-8D4D-0FBB8EF2ED3B
Figs. 2–4

**Type material:** HOLOTYPE: ♂ (in alcohol): BRAZIL, Rio de Janeiro, Rio de Janeiro, Parque Nacional da Tijuca, Trilha para Ponte Pênsil, MVO de Almeida leg., 17 Dec 2019, specimen number MNRJ#31,130 (MNRJ). PARATYPE: 1♀ (in alcohol), same data of holotype, specimen number MNRJ#31,743 (MNRJ) –.

**Diagnosis:** Second to fourth segment of palpus bicolored. Stripes on prescutum and presutural scutum with complete brown margins and lighter centers. Lateral region of the thorax appears to have a thick latitudinal stripe on the upper margin near prescutum and presutural scutum, including pleura and anepisternum. Wing clouded with spread light centers.

**Description** Male (Fig. 3). **Body length** 18.0 mm (Fig. 3A). **Wing length** 18.0 mm. **Head** Coloration light brown. **Rostrum** light brown, with a brown stripe laterally. **Nasus** short and stout. **Vertex** with a low bilobed vertical tubercle. **Antenna** 12-segmented. Scape yellowish, about three times longer than pedicel, pedicel yellow, Flagellomere ten light brown cylindrical flagellomeres, dark setae on dorsal view. **Palpus** dark brown. First segment light brown with a dark spot, second segment dark brown with white distal tip, third segment dark brown with white distal tip, fourth segment dark brown with white distal tip, fifth segment longer than previous segments combined, entirely brown. **Thorax. Prescutum and presutural scutum** with four longitudinal light brown stripes with complete brown margins and lighter centers, lateral ones darker. Background closer to pronotum and between lateral and center stripes with a brown spot. **Postsutural scutum** with four light brown spots, bottom one with a lighter center. Background on lateral margin, near wing base, darker. **Scutellum** brown with a dark thick central stipe. **Mediotergite** brown with a center dark stripe and dark lateral spots, not reaching central stripe (Fig. 3B). **Pleura** with dark brown latitudinal stripe near **Anepisternum** and a smaller spot near prescutum and presutural scutum. **Anepisternum** yellow background with a dark brown wide spot covering most of the area. **Katepisternum** bright yellow with dark brown thick spot on posterior margin. **Anepimeron** bright yellow with large center dark spot. **Katepimeron** bright yellow. **Meron** bright yellow with brown spot in posterior margin, between second and third coxa. **Anatergite** dark brown. **Katatergite** white with dark brown margin. **Coxa** yellow with faint small brown spot on anterior half. **Trochanter** yellow (Fig. 3C). **Femora** brown with dark subterminal ring. **Tibiae** brown. **Tarsi** dark brown. **Wing** extensively clouded by grayish brown in every cell, darker spots on mid-length of cell bm, closer to vein Cu, base of cell cua, margin of cells r5 and m2 and apex of vein A. Spots on cell br close to veins r-r, r-m, last portion of vein M; posts covering most of cells r3, r4, m3 and m4; spot on cell r5 large from base closer to discal cell, becoming interrupted at mid-length and with a smaller darker spot near margin; spots on cell bm close to base, following vein M and on center of the cell; discal cell with distal half near vein m-m and M_1+2_ darker, cell m1 and m2 with spots at the base of the cell and darker and smaller ones near wing margin; on cell cua a large darker spot near the base, and a fainter one from the center to wing margin; sparsely distributed at cell cup, leaving lighter center on the one closer to vein CuP; spot on anal lobe almost covering the entire cell. **Venation** Vein Rs curved and subequal to m-cu. Vein R_4_ curved in mid-length and straight towards wing margin, Vein R_5_ curved at mid length, Vein M_1+2_ about 1/3 of m-m, Squama bare (Fig. 3D). **Abdomen.** **Tergites** brown, darker laterally and with segments 1 and 2 lighter dorsally. **Sternites** yellow, posterolateral margin darker on segments 2-6. **Hypopygium**. **Tergite IX** shortly produced outwardly, its margin curved with few sparse setae (Fig. 3E). **Gonocoxite** broad in mid-length and apex narrower (Fig. 3F). **Lobe of gonostylus** with asymmetric general shape (resembled to a square), base portion, closer to gonocoxite, short and narrow, posterior portion broad with outer angle with a well-defined long lobe and a smaller one below and on the opposite angle three short triangular spines and dorsal face with an elongate and cylindrical flap. **Clasper of gonostylus** elongate and cylindrical, subequal in length to lobe of gonostylus. **Sternite VIII** with two small round projections (Fig. 3G).

**Description** Female (Figs. 4A–4F). **Body length** 19.0 mm. **Wing length** 19.0 mm. Generally similar to male except: second segment of **palpus** dark brown; **prescutum, presutural scutum** **and scutum** with stripes darker than male; **wing** with spots on the apex of the wing darker and more defined and the remaining fainter; **ovipositor** with **cercus** slender, broader at base; **hypogynial valve** broad almost same length of cercus.

**Etymology:** The species name *catia* is in honor of Dr. Catia Antunes de Mello-Patiu (1958-2021), Brazilian dipterist specialist in Sarcophagidae and Conopidae, former professor of the Departamento de Entomologia, Museu Nacional—UFRJ, Brazil. The name is noun in apposition.

**Remarks:** Tergite IX of *Ic. catia* **sp. nov.** closely resembles that of *Ic. euterpe*, but other characteristics of the genitalia differentiate the two species, such as the presence of a flap on the lobe of the gonostylus and the shape of the clasper of the gonostylus. *Icriomastax. catia* **sp. nov.** and *Ic. euterpe* are quite different regarding the rest of the body such as general color, being *Ic. catia* **sp. nov.** darker then *Ic. euterpe.*

**Distribution:** BRAZIL, *Rio de Janeiro*, Rio de Janeiro (type locality) (Fig. 2).

**Material examined:** BRAZIL (Fig. 2), *Rio de Janeiro* (type locality).

**Table:** 

**Figure 5:**
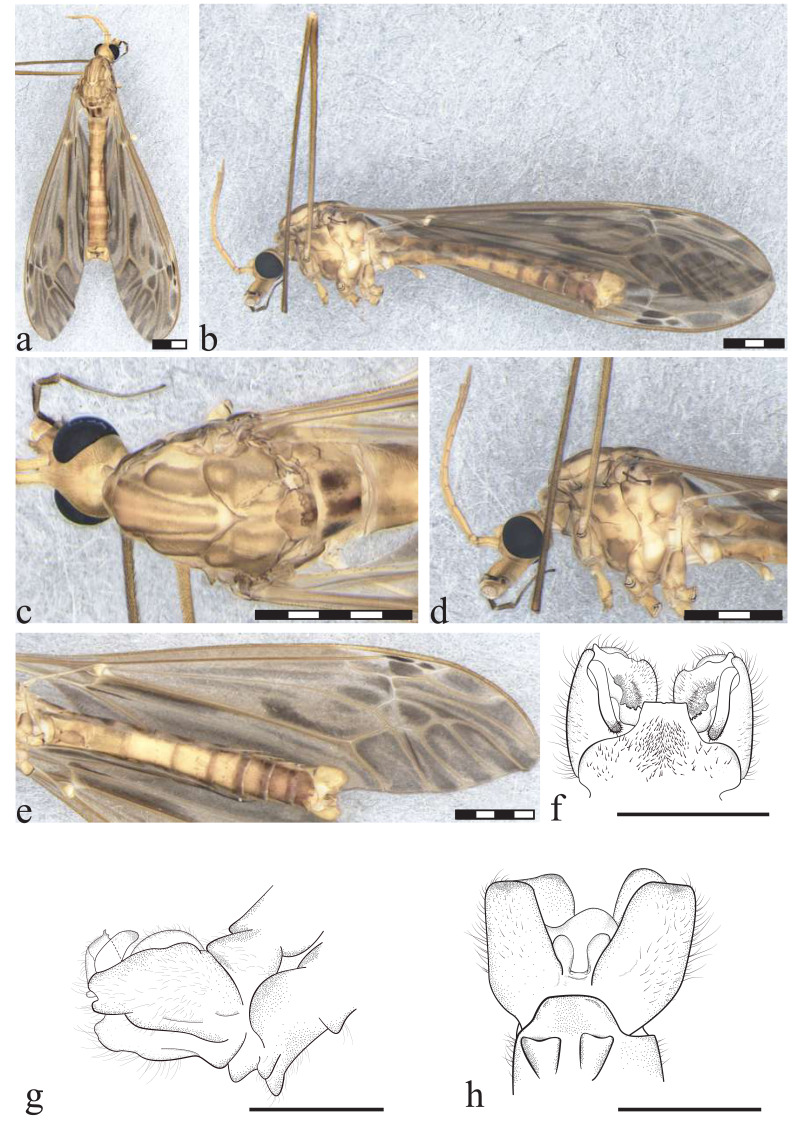
*Icriomastax coscaroni* sp. nov. Holotype. Male (MNRJ31,646). (A) Habitus, dorsal view. (B) Habitus, lateral view. (C) Thorax, dorsal view. (D) Thorax, lateral view. (E) Wing. Scale bar one mm. (F) Genitalia, dorsal view. (G) Genitalia lateral view. (H) Genitalia, ventral view. Scale bar 500 µm.

**Figure 6:**
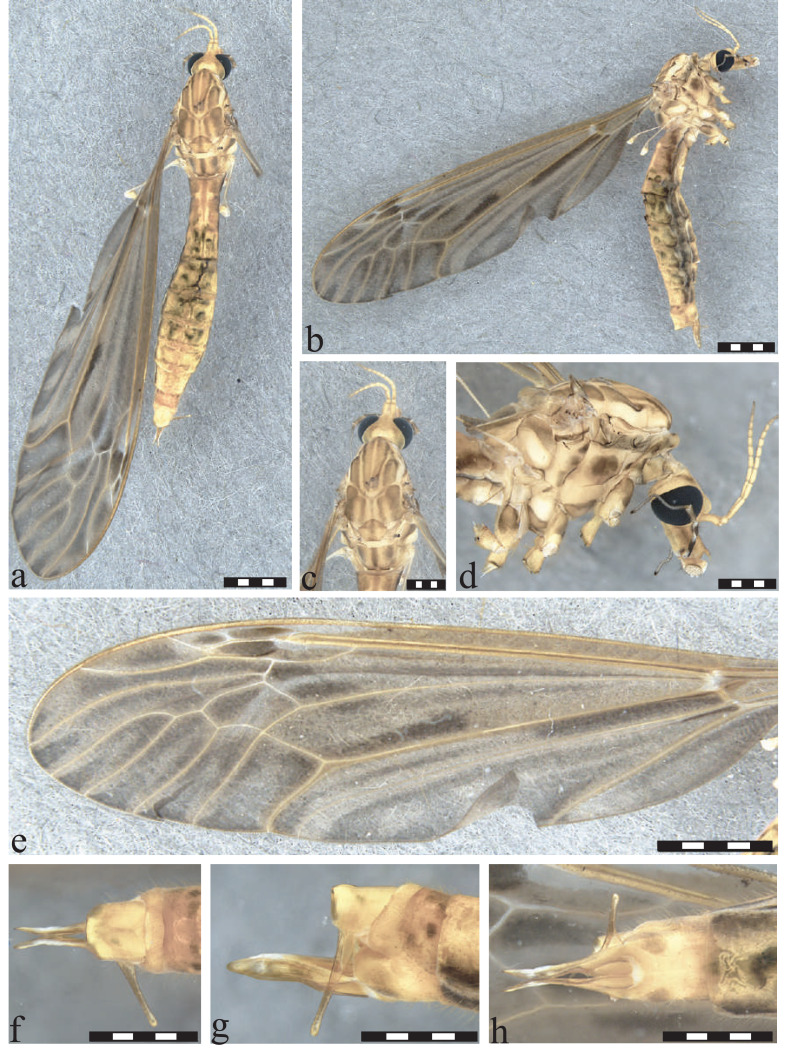
*Icriomastax coscaroni* sp. nov. Paratype. Female (MNRJ31,648). (A) Habitus, dorsal view. (B) Habitus, lateral view. Scale bar four mm. (C) Thorax, dorsal view. (D) Thorax, lateral view. Scale bar two mm. (E) Wing. Scale bar four mm. (F) Genitalia, dorsal view. (G) Genitalia lateral view. (H) Genitalia, ventral view. Scale bar one mm.

*Icriomastax coscaroni***sp. nov.**
urn:lsid:zoobank.org:act:371FD209-C8E3-426C-8981-02C0BFBFB191
Figs. 2, 5–6

**Type material:** HOLOTYPE: ♂ (in alcohol, terminalia dissected and stored with the adult): BRAZIL, São Paulo, Campos do Jordão, Parque Estadual Campos do Jordão, lago próximo do alojamento, armadilha CDC, S22°41′25.8″W45°29′08.9″, 1,533 m, LHG Azevedo, CCD Correa, IKL de Souza, AVG Falcón legs., 6 Oct. 2021, specimen number MNRJ#31,646 (MNRJ). –, PARATYPES: 1♀ (in alcohol), same data of holotype, specimen number MNRJ#31,647 (MNRJ) and 5♂ + 3♀ (in alcohol), same data of holotype, specimen numer MNRJ#31,648 (MNRJ).

**Diagnosis:** Antenna with last four segments slightly darker. Fourth segment of palpus with lighter anterior tip. Fifth segment of palpus longer than previous segments combined. Anepisternum and katepisternum with spots darker than the ones on anepimeron in males, but with similar shade on female. Anepisternum yellow with brown middle spot, almost circular especially in female. Wings with spots around cells m1, m2, m3 and m4 leaving a lighter center. Tergite IX with a plate produced outwardly with a square outline. Both males and females have pronounced spots, such as on the wings.

**Description:** Male (Fig. 5). **Body length** 12.0 mm (Figs. 5A–5B). **Wing length** 15.0 mm. **Head** Coloration light brown. **Rostrum** yellow, with a brown stripe laterally. **Nasus** with long yellow setae. **Vertex** with a high and bilobed vertical tubercle. **Antenna** 12-segmented. Scape yellow, softly wrinkled in dorsal view, about three times longer than pedicel; pedicel short and light yellow, Flagellomere ten light brown cylindrical flagellomeres, last four segments slightly darker. **Palpus** brown. First segment short rounded, brown with a dark spot, Second segment brown, Third segment brown, Fourth segment brown with lighter anterior tip, Fifth segment brown, mid-length somewhat lighter, longer than previous segments combined. **Thorax.** **Prescutum and presutural scutum** with four light brown stripes, the lateral ones darker and with complete dark margins. The central fainter but only with external margins, facing the lateral stripes. **Postsutural scutum** with four spots with lighter centers and darker margins near V-shaped suture, bottom one lighter. Posterolateral margin, near wing base, darker than background. **Scutellum** light brown background with faint brown middle stripe and dark spread spot on posterios margin. **Mediotergite** dark brown middle stripe and two dark large lateral spots not reaching the posterior margin nor the middle stripe (Fig. 5C). **Pleura** with dark brown latitudinal stripe. **Anepisternum** yellow with brown middle spot. **Katepisternum** with brown spot between first and second coxa. **Anepimeron** yellow with small three brown spots, the darker one near pleura and **Anepisternum**. **Katepimeron** uniformly yellow. **Meron** yellow with brown spot in posterior margin, between second and third coxa. **Anatergite** brown. **Katatergite** yellow. **Coxa** yellow with faint brown spot on anterior half. **Trochanter** yellow (Fig. 5D). **Femur** yellow near trochanter and becoming brown towards tibia. **Tibia** brown. **Wing** with large dark spots in every cell except cell Sc, darker ones on center of cell bm and base of cell cua. Spots also present near vein r-r and r-m, at last portion of vein M, center of cells r3, r4 and r5, around cells m1, m2, m3 and m4 leaving a lighter center, and spread on cell discal, cup and anal. **Venation** Vein Rs curved and subequal to m-cu. Vein R_4_ curved in mid-length and straight towards wing margin, Vein R_5_ curved at mid length, Vein M_1+2_ half m-m, Macrotrichia only on veins R, R_4_, R_5_, M_1_ and M_2_, Squama bare (Fig. 5E). **Abdomen.** **Tergites** light brown, darker on the posterior margin giving a striped appearance and with dark lateral stripe. **Sternites** 1 to 5 with a posterior stripe, remaining segments darker towards terminalia. **Hypopygium**. **Tergite IX** with a plate produced outwardly with a square outline and spread dark setae (Fig. 5F). **Gonocoxite** with apex narrower than mid-length and smoothly sinuous (Fig. 5G). **Lobe of gonostylus** with asymmetric general shape (resembled to a square), posterior portion broad with curved angles and with a dark flap with serrate margins on dorsal face. **Clasper of gonostylus** narrowly cylindrical shape with similar width through length and with dark thick setae at the apex. **Sternite VIII** with two small truncate projections (Fig. 5H).

**Description:** Female (Figs. 6A–6H). **Body length** 13.0 mm (Figs. 6A–6B). **Wing length** 15.0 mm. Generally similar to male except: **palpus** lighter than male with second segment brown, posterior tip white, third segment brown, both tips white, fourth segment brown, both tips white; **scutellum** light brown background with faint brown middle stripe; **mediotergite** as male, but lighter spots; **anepisternum** spot a little darker and wider than male; **katepisternum** spot darker and thiner than male; **anepimeron** spots darker than male; **katepimeron** yellow with brown middle spot; **meron** spot darker than male; **coxa** spots darker than male; **wings** with vein M_1+2_ subequal to m-m; **sternites** light brown with dark lateral spots; **ovipositor** with **cercus** slender, broader at base; **hypogynial valve** long, almost same length as cercus, and stout.

**Etymology:** The species name *coscaroni* is in honor of Dr. Sixto Coscarón (1926-2022), Argentine dipterist specialist in Simuliidae and Tabanidae, former professor of the Facultad de Ciencias Naturales y Museo de La Plata, Argentina.

**Remarks.** *Icriomastax coscaroni* **sp. nov.** stands out due to the strong wing spots, which are darker and more pronounced than in most species of the group. Although it has darker lateral stripes than the central ones on the prescutum and presutural scutum, as seen in *Ic. calliope* for instance, the center of the stripes is lighter, creating a greater contrast than that observed in *Ic. helios*. The same is observed for the spots on the scutum. Another distinguishing feature is the dark central spot, almost circular especially in the female, on the anepisternum, similar to that found in *Ic. ocellata* and *Ic. lopesae* **sp. nov.**, but stronger and more prominent than in those species. Also similar to *Ic. ocellata* is the square shape of the projection of tergite IX, but in *Ic. coscaroni* **sp. nov.** this shape is wider and angular, while in *Ic. ocellata* it is slightly narrower with curved sides.

**Distribution:** BRAZIL (Fig. 2), *Rio de Janeiro*, Teresópolis. *São Paulo*, Campos do Jordão (type locality). *Santa Catarina*: Santo Amaro da Imperatriz.

**Material examined:** BRAZIL, *São Paulo:* Campos do Jordão; Parque Estadual Campos do Jordão; lago próximo do alojamento; S22°41′25.8 W45°29′08.9″; 1,533 m.a.s.l.: 1♂ Alcohol (MNRJ#31,646, Holotype); 5♂ + 4♀ Alcohol (MNRJ#31,647 and MNRJ#31,648, Paratype). *Rio de Janeiro*: Teresópolis, Parque Nacional da Serra dos Órgãos, Barragem do Rio Beija Flor; 1,158 m.a.s.l.; S22°27′03.8″W43°00′02.5″: 1♂; (MNRJ#31,504); Teresópolis, Parque Nacional da Serra dos Órgãos, Cachoeira do Papel: 2♂ (MNRJ#31,487). *Santa Catarina*: Santo Amaro da Imperatriz, Pagará-Colônia, Sta. Luzia, Malaise brejo, S27°38′31″W48°45′17″1♂ (MNRJ#30,992), 4♂ (MNRJ#31,006); Orleans, Malaise, S28°12′42″W49°27′21″, 1♂ (MNRJ#30,996); Grão-Pará, Parque Estadual da Serra Furada, Malaise, S28°11′23″W49°23′32″, 1♂ (MNRJ#30,999); Urubici, Cascata Avencal, S28°02′48″W49°37′01″, 1♂ (MNRJ#31,001); Rio do Bispo, Casa Hipster 1♀ (MNRJ#31,005).

**Table:** 

**Figure 7:**
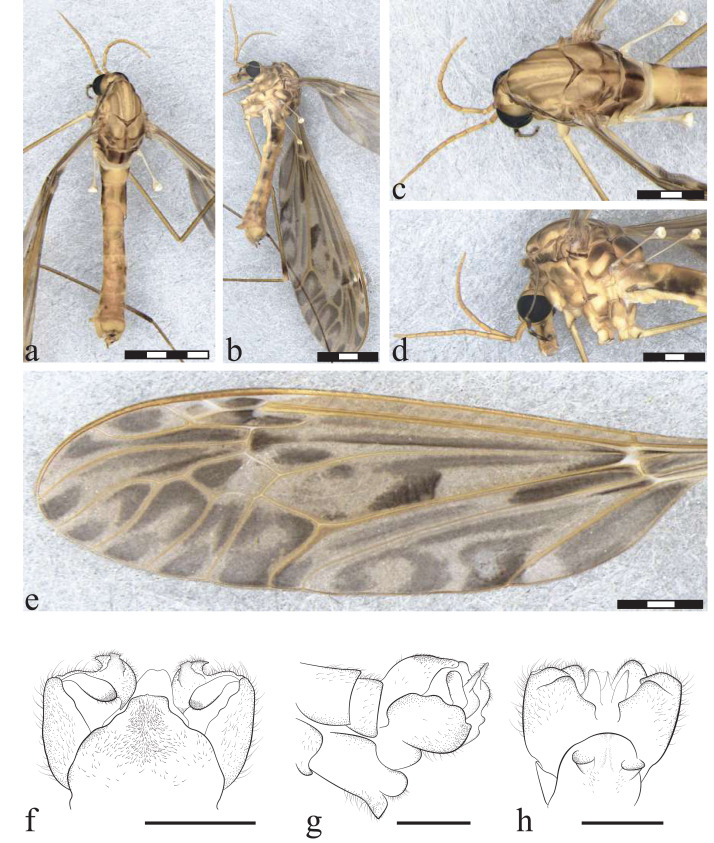
*Icriomastax craigi* sp. nov. Holotype. Male (MNRJ31,231). (A) Habitus, dorsal view. (B) Habitus, lateral view. Scale bar two mm. (C) Thorax, dorsal view. (D) Thorax, lateral view. (E) Wing. (F) Genitalia, dorsal view. (G) Genitalia, laterial view. (H) Genitalia, ventral view. Scale bar one mm.

**Figure 8:**
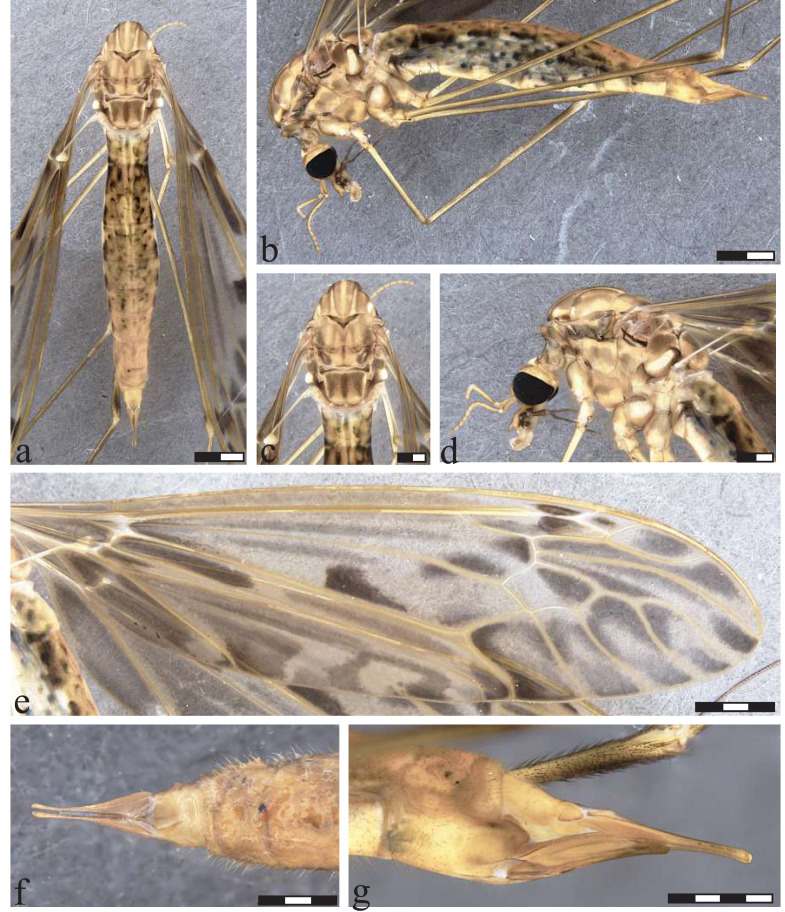
*Icriomastax craigi* sp. nov. Paratype. Female (MNRJ31,652). (A) Habitus, dorsal view. (B) Habitus, lateral view. Scale bar two mm. (C) Thorax, dorsal view. (D) Thorax, lateral view. Scale bar one mm. (E) Wing. Scale bar two mm. (F) Genitalia, dorsal view. (G) Genitalia, lateral view. Scale bar one mm.

*Icriomastax craigi***sp. nov.**
urn:lsid:zoobank.org:act:D4813148-81C7-42BD-AD90-0AA2CF7042EC
Figs. 2 and 7–8

**Type material:** HOLOTYPE: ♂ (in alcohol, terminalia dissected and stored with the adult): BRAZIL, Rio de Janeiro, Teresópolis, Parque Nacional da Serra dos Órgãos, Trilha Pedra do Sino, S22°27′11.2″W43°00′53.4″, 1,700 m, LH Gil-Azevedo & AC Mendes leg., 04 May 2019, specimen number MNRJ#31,231 (MNRJ). PARATYPE: ♀ (in alcohol), same data of holotype, specimen number MNRJ#31,652 (MNRJ). 4♂ + 1♀ (in alcochol). BRAZIL, Minas Gerais, Itamonte, Parque Nacional do Itatiaia, road between Casa de Pedra and Brejo da Lapa, specimen number MNRJ#31,160 (MNRJ).

**Diagnosis:** Mediotergite almost entirely dark brown. Spots on anepisternum and katepisternum connected on males. Wing with large and almost equally dark spots in every cell. Tergite IX with a high plate produced outwardly with smoothly sinuous margins and distal margin with a short triangular projection. Gonocoxite round. Lobe of gonostylus with with a strong curve on posterior margin and pointed extremities in dorsal view, dorsal face with a dark flap with larger base and narrower apex Both males and females have pronounced spots, such as on the wings.

**Description** Male (Fig. 7). **Body length** 18.0 mm (Figs. 7A–7B). **Wing length** 20.0 mm. **Head** Coloration light brown. **Rostrum** light brown, with a brown stripe laterally. **Nasus** short and stout. **Vertex** with a low bilobed vertical tubercle. **Antenna** 12-segmented. Scape light brown, about four times longer than pedicel, pedicel light brown, Flagellomere ten light brown cylindrical flagellomeres, dark setae on dorsal view. **Palpus** dark brown. First segment light brown with a dark spot, second segment dark brown with white distal tip, Third segment dark brown with white distal tip, Fourth segment dark brown with white distal tip, Fifth segment longer than previous segments combined, getting lighter through length, distal tip black. **Thorax.** **Prescutum and presutural scutum** with four longitudinal opaque brown stripes, lateral ones darker and with complete brown margins. Central stripes fainted closer to scutum and with margins facing lateral stripes more defined. Background closer to pronotum and between lateral and center stripes with a brown spot. **Postsutural scutum** with four light brown spots, bottom one with a lighter center. Background on lateral margin, near wing base, darker. **Scutellum** brown with a dark thin central stipe. **Mediotergite** brown with a center dark stripe and wide dark lateral spots, almost reaching central stripe (Fig. 7C). **Pleura** with dark brown latitudinal stripe near **Anepisternum** and a smaller spot near prescutum and presutural scutum. **Anepisternum** light brown with a wide grayish spot covering most of the area. **Katepisternum** light brown background with a large spot near **Anepisternum** and a weaker spot on posterior margin. **Anepimeron** light brown with a thin brown spot following the suture between **Anepisternum** and katepisternum. **Katepimeron** yellow. **Meron** yellow with brown spot in posterior margin, between second and third coxa. **Anatergite** dark brown. **Katatergite** white with dark brown margin. **Coxa** yellow with a faint brown spot on the anterior half. **Trochanter** yellow (Fig. 7D). **Femur** brown with dark subterminal ring. **Tibia** brown. **Tarsus** dark brown. **Wing** extensively clouded by brown in every cell, most of them equally dark. Spots on cell br close to veins r-r, r-m, last portion of vein M and a long center one from the base to origin of vein Rs; wide dark spot on center of cell r3; spot on cell r4 covering almost entire cell but smaller near wing margins; spot on cell r5 large from base closer to discal cell, becoming interrupted at mid-length and with a smaller spot near margin; spots on cell bm close to base, following vein M and on center of the cell; discal cell with distal half near vein m-m and M_1+2_ darker, cells m1, m2, m3 and m4 with large spots at base becoming interrupted at mid-length and with a smaller spot near margin; on cell cua a large darker spot near the base, and fainter at the center and close to wing margin; sparsely distributed at cell cup, leaving lighter centers on the large ones; spot on anal lobe almost covering the entire cell, except closer to apex of vein A near margin;. **Venation** Vein Rs curved and subequal to m-cu. Vein R_4_ curved in mid-length and sinuous towards wing margin, Vein R_5_ curved at mid length, Vein M_1+2_ 2/3 of m-m, Squama bare (Fig. 7E). **Abdomen.** **Tergites** light brown, darker on posterolateral margin of segments 2-4, remaining segments fainter. **Sternites** yellow, posterolateral margin darker on segments 7-8. **Hypopygium**. **Tergite IX** with a plate produced outwardly, smoothly sinuous at antero lateral margins, the posterior angles strongly curved and distal margin with a short triangular projection (Fig. 7F). **Gonocoxite** with a round shape (Fig. 7G). **Lobe of gonostylus** with asymmetric general shape with a strong curve on posterior margin and pointed extremities in dorsal view, dorsal face with a dark flap with larger base and narrower apex. **Clasper of gonostylus** cylindrical shape with similar width through length and with dark thick setae at the apex. **Sternite VIII** with two small truncate projections (Fig. 7H).

**Description** Female (Figs. 8A–8G). **Body length** 16.0 mm (Figs. 8A–8B). **Wing length** 20.0 mm. Generally similar to male except: **palpus** with fifth segment longer than previous segments combined, entirely brown; prescutum and presutural scutum with central stripe not fainted closer to postsutural scutum; **katepisternum** with posterior spot thinner than male; **anepimeron** with spot wider than male; **wing** with most of spots outline less defined than male; vein M_1+2_ subequal to m-m; t**ergites** light brown, darker on posterolateral margin of segments 2-5, remaining segments fainter; **sternites** yellow, posterolateral margin darker on segments 2-6; **ovipositor** with **cercus** slender, broader at base; **hypogynial valve** broad reaching half of cercus length.

**Etymology.** The species name *craigi* is in honor of Dr. Douglas “Doug Senior” A. Craig (1939-2020), New Zealand dipterist specialist in Simuliidae and Blephariceridae, former professor of the University of Alberta, Canada.

**Remarks.** *Icriomastax craigi* **sp. nov.** is similar to *Ic. monnei* **sp. nov.**, differing mainly in the male genitalia. Another difference, though much more subtle, is on the lateral thorax of the females, where *Ic. craigi* **sp. nov.** has lighter spots and there is no connection between the spots on the anepisternum and katepisternum as seen in the female of *Ic. monnei* **sp. nov.**

**Distribution:** BRAZIL (Fig. 2), Minas Gerais, Itamonte; Rio de Janeiro, Teresópolis (type locality).

**Material examined:** BRAZIL, *Rio de Janeiro*, Teresópolis, Parque Nacional da Serra dos Órgãos, Trilha da Pedra do Sino, S22°27′11.2″W43°00′53.4″, 1,700 m.a.s.l.: 1♂ Alcohol (MNRJ#31,231, Holotype). *Rio de Janeiro*, Teresópolis, Parque Nacional da Serra dos Órgãos, Trilha da Pedra do Sino, S22°27′11.2″W43°00′53.4″, 1,700 m.a.s.l. 1♀ Alcohol (MNRJ#31,652, Paratype). *Minas Gerais*, Itamonte, Parque Nacional do Itatiaia, road between Casa de Pedra and Brejo da Lapa: 3♂ + 1♀ (MNRJ#31,160, Paratype); Itamonte, Parque Nacional do Itatiaia, road between Casa de Pedra and Brejo da Lapa 1♂ (MZ053526).

**Table:** 

**Figure 9:**
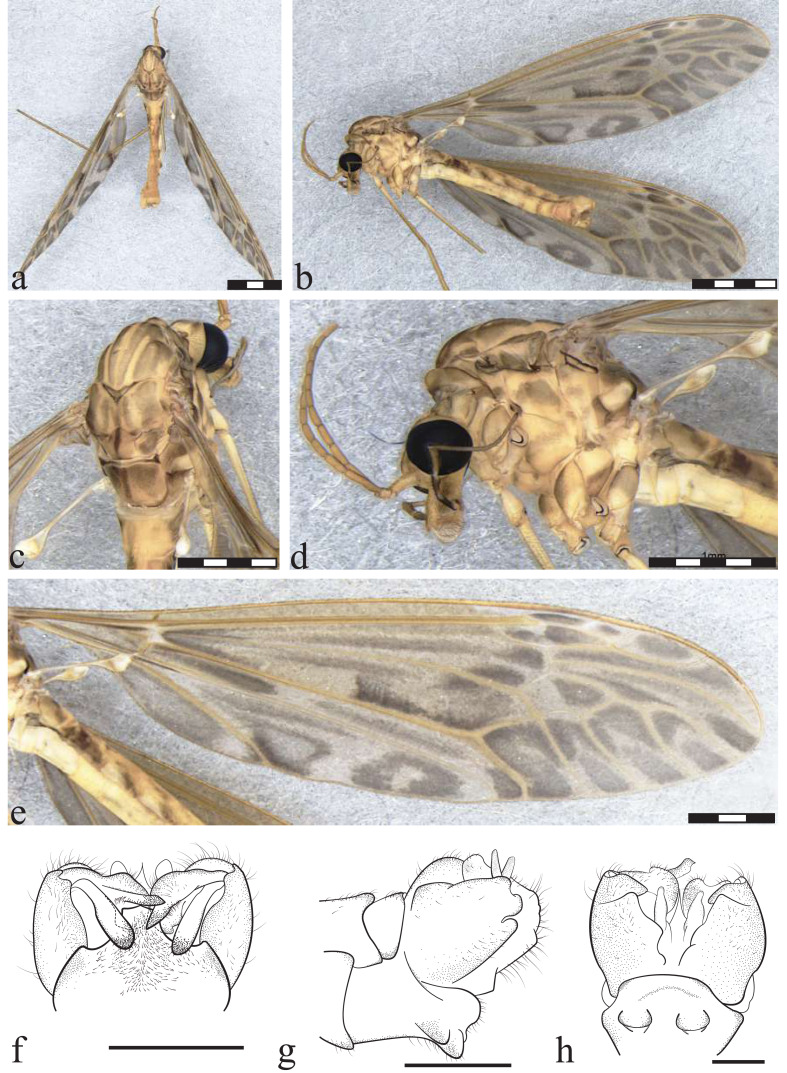
*Icriomastax lopesae* sp. nov. Holotype. Male (MNRJ31,133). (A) Habitus, dorsal view. (B) Habitus, lateral view. Scale bar two mm. (C) Thorax, dorsal view. (D) Thorax, lateral view. (E) Wing. Scale bar one mm. (F) Genitalia, dorsal view. G. Genitalia, lateral view. (H) Genitalia, ventral view. Scale bar one mmm.

*Icriomastax lopesae***sp. nov.**
urn:lsid:zoobank.org:act:5D894885-8267-4D24-B24A-4B89D499D4A4
Figs. 2 and 9

**Type material:** HOLOTYPE: ♂ (in alcohol, terminalia dissected and stored with the adult): BRAZIL, *Rio de Janeiro*, Teresópolis, Trilha da Pedra do Sino, Travessia Teresópolis-Petrópolis, 1,900 m–1,700 m, LH Gil-Azevedo leg., 04 Oct 2019, specimen number MNRJ#31,133 (MNRJ). PARATYPE: ♂ (in alcohol)–BRAZIL, *Rio de Janeiro*, Teresópolis, Parque Nacional da Serra dos Órgãos, Trilha da Pedra do Sino, 1,300–1,500 m.a.s.l., LH Gil-Azevedo leg., 04 May 2019, specimen numberMNRJ#31,651 (MNRJ).

**Diagnosis:** Second to fourth segment of palpus bicolored. Central stripes on prescutum and presutural scutum with margins facing lateral stripes more defined. Central spot on anepisternum darker than those on katepisternum and anepimeron. Wing clouded with light centers. Tergite IX with a high plate produced outwardly, with acute lateral angles and a small triangle on the apex.

**Description** Male (Fig. 9). **Body length** 18.0 mm (Figs. 9A–9B). **Wing length** 19.0 mm. **Head** Coloration light brown. **Rostrum** light brown, with a brown stripe laterally. **Nasus** short and stout. **Vertex** with a low bilobed vertical tubercle. **Antenna** 12-segmented. Scape light brown, about four times longer than pedicel, pedicel light brown, Flagellomere ten light brown cylindrical flagellomeres, dark setae on dorsal view. **Palpus** dark brown. First segment light brown with a dark spot, second segment dark brown with white distal tip, third segment dark brown with white distal tip, fourth segment both tips white, fifth segment longer than previous segments combined, entirely brown. **Thorax.** **Prescutum and presutural scutum** with four longitudinal light brown stripes with margins facing lateral stripes more defined, lateral ones a little darker. Background closer to pronotum and between lateral and center stripes with a brown spot. **Postsutural scutum** with four light brown spots, bottom one with a lighter center. Background on lateral margin, near wing base, darker. **Scutellum** brown with a dark thick central stipe. **Mediotergite** brown with a center dark stripe and dark lateral spots, closer to proximal margin and reaching half of mediotergite (Fig. 9C). **Pleura** with dark brown latitudinal stripe near **Anepisternum** and a smaller spot near prescutum and presutural scutum. **Anepisternum** light brown with a center grayish spot. **Katepisternum** light brown background with a very weak grayish narrow spot at center. **Anepimeron** light brown with three small faint spots. **Katepimeron** yellow with faint brown middle spot. **Meron** yellow with brown spot in posterior margin, between second and third coxa. **Anatergite** dark brown. **Katatergite** white. **Coxa** light brown with faint small brown spot on anterior half. **Trochanter** light brown (Fig. 9D). **Femur** light brown. **Tibia** brown. **Wing** extensively clouded by grayish brown in every cell, most of them equally dark. Spots on cell br close to veins r-r, r-m, last portion of vein M and a long fainter one from the base to origin of vein Rs; wide spot on center of cell r3; spot on cell r4 covering almost entire cell; spot on cell r5 covering almost entire cell but somewhat darker following M and M_1+2_ and close to wing margin; spots on cell bm close to base, following vein M and on center of the cell; discal cell with distal half near vein m-m and M_1+2_ darker, cells m1, m2, m3 and m4 with large spots at base becoming interrupted at mid-length and with a smaller spot near margin; on cell cua a large darker spot near the base, a fainter at the center and a darker close to wing margin; spots sparsely distributed at cell cup, leaving lighter centers on the large ones; spot on anal lobe almost covering the entire cell, except at base and closer to apex of vein A near margin;. **Venation** Vein Rs curved and subequal to m-cu. Vein R_4_ curved in mid-length and straight towards wing margin, Vein R_5_ curved at mid-length, Vein M_1+2_ subequal to m-m, Squama bare (Fig. 9E). **Abdomen.** **Tergites** light brown, darker laterally becoming fainter towards genitalia. **Sternites** yellow, posterolateral margin a littler darker on segments 3-5. **Hypopygium**. **Tergite IX** with a high plate produced outwardly, with acute lateral angles and a small triangle on the apex (Fig. 9F). **Gonocoxite** with a broader mid-length and a narrower apex (Fig. 9G). **Lobe of gonostylus** with asymmetric general shape (resembles a square), with an elongate and narrow flap on dorsal face. **Clasper of gonostylus** cylindrical shape with similar width through length and with dark thick setae at the apex. **Sternite VIII** with two small truncate projections (Fig. 9H).

**Etymology.** The species name *lopesae* is in honor of Dr. Sônia Maria Lopes Fraga (1950-2021), Brazilian specialist in Blattaria and Diptera, former professor of the Departamento de Entomologia, Museu Nacional—UFRJ, Brazil.

**Remarks.** The wing spots with lighter centers are very similar to the pattern found in *Ic. monnei* **sp. nov.** and *Ic. craigi* **sp. nov.** However, *Ic. lopesae* **sp. nov.** does not have a broadly dark mediotergite or a dark spot on the lower margin of the katepisternum between the first and second coxae as seen in *Ic. monnei* **sp. nov.** and *Ic. craigi* **sp. nov.** The plate produced on tergite IX is similar in height to *Ic. monnei* **sp. nov.**, but the shape of the external angles, both of the plate and the lateral of the tergite, is not curved. Additionally, the long and narrow shape of the flap on the lobe of the gonostylus bears no resemblance to what is found in *Ic. monnei* **sp. nov.** and *Ic. craigi* **sp. nov.**

**Distribution:** BRAZIL(Fig. 2), *Rio de Janeiro*, Teresópolis (type locality).

**Material examined:** BRAZIL, *Rio de Janeiro*, Teresópolis, Parque Nacional da Serra dos Órgãos, Trilha da Pedra do Sino; Travessia Teresópolis-Petrópolis; 1,700–1,900 m.a.s.l. 1♂ Alcohol (MNRJ#31,133, Holotype). Teresópolis, Parque Nacional da Serra dos Órgãos, Trilha da Pedra do Sino, 1,300–1,500 m.a.s.l. 1♂ Alcohol (MNRJ#31,651, Paratype).

**Table:** 

**Figure 10:**
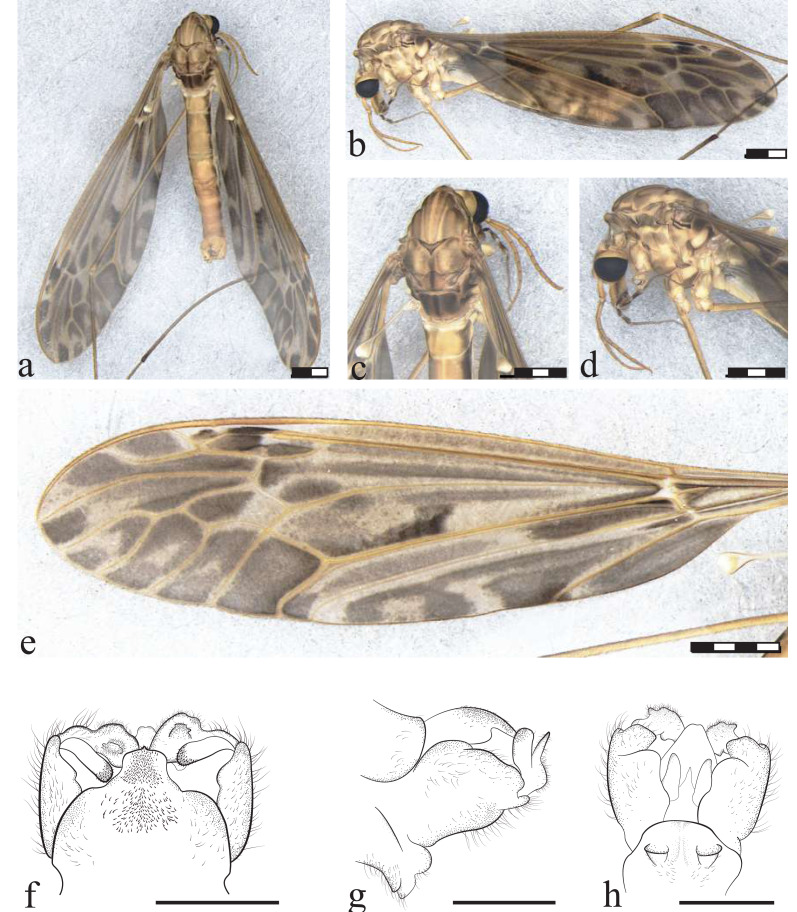
*Icriomastax monnei* sp. nov. Holotype. Male (MNRJ31,649). (A) Habitus, dorsal view. (B) Habitus, lateral view. (C) Thorax, dorsal view. (D) Thorax, lateral view. (E) Wing. (F) Genitalia, dorsal view. (G) Genitalia, lateral view. (H) Genitalia, ventral view. Scale bar one mm.

**Figure 11:**
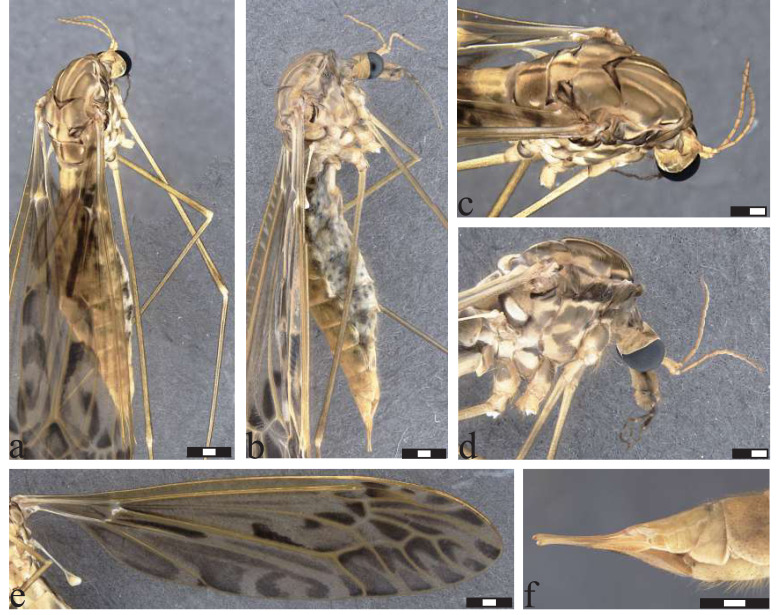
*Icriomastax monnei* sp. nov. Paratype. Female (MNRJ31,653). (A) Habitus, dorsal view. (B) Habitus, lateral view. Scale bar two mm.C. Thorax, dorsal view. (D) Thorax, lateral view. Scale bar one mm. (E) Wing. Scale bar two mm. (F) Genitalia, lateral view. Scale bar one mm.

*Icriomastax monnei***sp. nov.**
urn:lsid:zoobank.org:act:6092A27F-4A88-4EAC-83BE-0C514D347113
Figs. 2, 10 and 11

**Type material:** HOLOTYPE: ♂ (in alcoholterminalia dissected and stored with the adult): BRAZIL, *Rio de Janeiro*, Teresópolis, Parque Nacional da Serra dos Órgãos, Riacho atrás da Casa do Pesquisador, CCD Corrêa leg., 29 Mar 2022, specimen number MNRJ#31,649 (MNRJ). PARATYPES: 2♂ (in alcohol) (MNRJ#31,136) –*Minas Gerais*, Itamonte, Parque Nacional do Itatiaia, Casa de Pedra, S22°22′05.6″W44°44′42.5″; 2012 m.a.s.l., specimen number MNRJ#31,136 (MNRJ).♀ (in alcohol): Brazil, *Rio de Janeiro*, Teresópolis, Parque Nacional da Serra dos Órgãos, Travessia Teresópolis-Petrópolis, S22°27′11,2″W43°00′53,4″, 1,700 m, LH Gil-Azevedo & AC Mendes leg., 23 May 2019, specimen number MNRJ#31,653 (MNRJ). ♂ (in alcohol): Teresópolis, Parque Nacional da Serra dos Órgãos, Casa do Pesquisador, S22°27′17.1″W42°59′50.0″, 1,134 m.a.s.l.; CDC trap, 29-30 Oct 2019, specimen number MNRJ#31,215 (MNRJ).7♂1♀ (in alcohol): Teresópolis, Parque Nacional da Serra dos Órgãos, Travessia Teresópolis-Petrópolis, S22°26′55.3″W43°00′45.1″, 29 Mar 2020, specimen number MNRJ#31,497 (MNRJ)2♂ (in alcohol): Teresópolis; Parque Nacional da Serra dos Órgãos; Travessia Teresópolis-Petrópolis, S22°26′55.3″W43°00′45.1″, 22 May 2019, specimen number MNRJ#31,303 (MNRJ). ♂ (in alcohol): Teresópolis; Parque Nacional da Serra dos Órgãos; Travessia Teresópolis-Petrópolis, S22°27′11.2″W43°00′53.4″, 23 May 2019, specimen number MNRJ#31,322 (MNRJ); 2♂ (in alcohol): Teresópolis; Parque Nacional da Serra dos Órgãos; Trilha Pedra do Sino, 4 Oct 2019, specimen number MNRJ#31,137 (MNRJ). 1♂ (in alcohol): Teresópolis; Parque Nacional da Serra dos Órgãos; Trilha Pedra do Sino, Travessia Teresópolis-Petrópolis, specimen number MNRJ#31,131 (MNRJ).. 1♂ (in alcohol) (MNRJ#31,134). Teresópolis; Parque Nacional da Serra dos Órgãos; Alojamento, specimen number MNRJ#31,134 (MNRJ). 1♂3♀ (in alcohol): Teresópolis; Parque Nacional da Serra dos Órgãos, 29 May 2022, specimen number MNRJ#31,558 (MNRJ). ♀ (in alcohol): Teresópolis; Parque Nacional da Serra dos Órgãos, Riacho atrás da Casa do Pesquisador, 29 Mar 2022, specimen number MNRJ#31,650 (MNRJ).

**Diagnosis:** Mediotergite almost entirely dark brown. Spots on anepisternum placed at the center, not forming a stripe. Spots on katepisternum forming a shape the resembles a zigzag. All spots on anepisternum and katepisternum are similar in shade to the ones on anepimeron. Wing with large and almost equally dark spots in every cell. Cell m4 is completely dark. Tergite IX with a high plate produced outwardly, smoothly sinuous at antero lateral margins, the posterior angles strongly curved and distal margin with a short triangular projection. Both males and females have pronounced spots, such as on the wings.

**Description** Male (Fig. 10). **Body length** 10.0 mm (Figs. 10A–10B). **Wing length** 10.0 mm. **Head** Coloration light brown. **Rostrum** light brown, with a brown stripe laterally. **Nasus** with long yellow setae. **Vertex** with a high and bilobed vertical tubercle. **Antenna** 12-segmented. Scape light brown, about four times longer than pedicel, pedicel light brown, Flagellomere ten light brown cylindrical flagellomeres, dark setae on dorsal view. **Palpus** brown. First segment dark brown, Second segment brown, posterior tip white, Third segment dark brown with both tips white, Fourth segment dark brown with both tips white, Fifth segment brown, longer than the first four combined. **Thorax.** **Prescutum and presutural scutum** with four light brown stripes, the lateral ones darker and with complete dark margins. The central fainter but only with external margins, facing the lateral stripes. **Postsutural scutum** with four faint spots with lighter centers and darker margins near V-shaped suture. Posterolateral margin, near wing base, darker than background. **Scutellum** light brown background with dark brown middle stripe. **Mediotergite** almost entirely dark brown with dark middle stripe (Fig. 10C). **Pleura** with dark brown latitudinal stripe near **Anepisternum** and a smaller spot near prescutum and presutural scutum. **Anepisternum** yellow with brown middle spot. **Katepisternum** yellow with grayish brown spot in the center and a darker one on posterior margin, between coxa 1 and 2. The two spots are connected forming a shape the resembles a zigzag. **Anepimeron** yellow with three small faint spots. **Katepimeron** yellow with brown middle spot. **Meron** yellow with dark brown spot in posterior margin, between second and thrid coxa. **Anatergite** dark brown. **Katatergite** white with dark brown margin. **Coxa** yellow with faint brown spot on anterior half. **Trochanter** yellow (Fig. 10D). **Femur** light brown with dark subterminal ring. **Tibia** brown, getting darker near tarsus. **Tarsus** dark brown. **Wing** with large and almost equally dark spots in every cell, present along cell br and near vein r-r and r-m, half discal cell; spread on cell bm, cua, cup and anal; almost entire cells r3, r4, m4 and anal, around cells r5, m1, m2 and m3 leaving a lighter center. **Venation** Vein Rs curved and subequal to m-cu. Vein R_4_ curved in mid-length and straight towards wing margin, Vein R_5_ curved at mid length, Vein M_1+2_ subequal to m-m, Squama bare (Fig. 10E). **Abdomen.** **Tergites** light brown, darker on the posterior margin giving a striped appearance and with dark lateral stripe. **Sternites** light brown, darker on the posterior margin giving a striped appearance. **Hypopygium**. **Tergite IX** with a high plate produced outwardly, smoothly sinuous at antero lateral margins, the posterior angles strongly curved and distal margin with a short triangular projection (Fig. 10F). **Gonocoxite** with a lobe near base on ventral face and apex narrower than mid-length (Fig. 10G). **Lobe of gonostylus** with asymmetric general shape with anterior portion near base long and narrow and posterior apex wide and sinuous on both dorsal and ventral margins, dorsal face with a dark flap with sinuous margins. **Clasper of gonostylus** cylindrical shape with similar width through length and with dark thick setae at the apex. **Sternite VIII** with two small truncate projections (Fig. 10H).

**Description** Female (Figs. 11A–11F). **Body length** 18 mm (Figs. 11A–11B). **Wing length** 18 mm. Generally similar to male except: flagellomere segments 4–8 weakly curved on ventral face; **Postsutural scutum** with darker upper spot; **mediotergite** large spots lighter and not as spread as male; **anepisternum** spot a little darker and wider than male; **katepisternum** center spot darker and posterior stripe fainter than male; **anepimeron** with darker spots than male; **katepimeron** uniformly yellow; **anatergite** lighter than male; **katatergite** darker and thicker margin than male; **coxa** spots darker than male; **femur** subterminal ring lighter than male; **tergites** posterior margin lighter; **sternites** bright yellow with faint lateral spots of segments 2 to 6; **ovipositor** with **cercus** slender, broader at base; **hypogynial valve** broad reaching half of cercus length.

**Etymology.** The species name *monnei* is in honor of Dr. Miguel Angel Monné Barrios (1938-2024), Uruguayan specialist in Coleoptera, former professor of the Departamento de Entomologia, Museu Nacional—UFRJ, Brazil.

**Remarks.** The present species is similar to *Ic. craigi* **sp. nov.** The main differences are in the male terminalia, where *Ic. monnei* **sp. nov.** has a higher tergite IX, gonocoxite with a narrower apex and lobe of gonostylus and its flap with a distinct outline.

**Distribution:** BRAZIL (Fig. 2), *Minas Gerais*, Itamonte; *Rio de Janeiro*, Teresópolis (type locality).

**Material examined:** BRAZIL, *Rio de Janeiro*, Teresópolis, Parque Nacional da Serra dos Órgãos, Riacho atrás da Casa do Pesquisador: 1♂ Alcohol (MNRJ#31,649, Holotype). *Rio de Janeiro*, Teresópolis, Parque Nacional da Serra dos Órgãos; Travessia Teresópolis-Petrópolis; S22°27′11,2″W43°00′53,4″, 1,700 m.a.s.l.: 1♀ Alcohol (MNRJ#31,653, Paratype). *Minas Gerais*, Itamonte, Parque Nacional do Itatiaia, Casa de Pedra, S22°22′05.6″W44°44′42.5″; 2012 m.a.s.l.: 2♂ (MNRJ#31,136, Paratype). *Rio de Janeiro*, Teresópolis, Parque Nacional da Serra dos Órgãos, Casa do Pesquisador, S22°27′17.1″W42°59′50.0″, 1134 m.a.s.l.; CDC trap: 1♂ (MNRJ#31,215, Paratype); 7♂1♀; (MNRJ#31,497, Paratype). Teresópolis, Parque Nacional da Serra dos Órgãos, Travessia Teresópolis-Petrópolis, S22°26′55.3″W43°00′45.1″: 2♂ (MNRJ#31,303, Paratype); 1♂ (MNRJ#31,322, Paratype); 2♂ (MNRJ#31,137, Paratype); 1♂ (MNRJ#31,131, Paratype). Teresópolis; Parque Nacional da Serra dos Órgãos; Alojamento: 1♂ (MZ053527, Paratype). Teresópolis; Parque Nacional da Serra dos Órgãos; Casa do Pesquisador: 1♂3♀ (MNRJ#31,558, Paratype); 1♀ (MZ053528, Paratype).

## Conclusions

This study presents the first phylogenetic analysis of the crane fly genus *Icriomastax*, clarifying its monophyly and stablishing its status as a distinct genus within Tipulidae. The genus now includes 15 valid species, including five newly described and one transferred species.

The recovered phylogeny offers insights into character evolution within the group and raises broader questions about South Pacific Track biogeographic patterns in crane flies. The approach adopted here demonstrates that well-structured morphological data remain indispensable for resolving taxonomic and evolutionary questions in underexplored lineages.

##  Supplemental Information

10.7717/peerj.21121/supp-1 Supplemental Information 1 Terminal taxa included in the phylogenetic analysis and corresponding examined material

10.7717/peerj.21121/supp-2 Supplemental Information 2 Data Matrix for the cladistics analyses for *Icriomastax* (“-” codes for inapplicable data; “?” for unavailable) Polymorphisms are represented by the letter A (0+1), B (0+2), C (0+3), D (1+2), E (1+3), F (2+3); G (0+1+2), H (0+1+3).

10.7717/peerj.21121/supp-3 Supplemental Information 3 Characters list

10.7717/peerj.21121/supp-4 Supplemental Information 4 One of the 12 most parsimonious trees obtained through EW, chosen to represent character distribution Circles represent apomorphies (white = homoplastic; black = non-homoplastic). Numbers above the circles indicate character numbers; numbers below indicate character states. Characters with ambiguous optimization were treated under ACCTRAN.

## Additional Information and Declarations

### Data Availability

The following information was supplied regarding data availability: The raw data is available in the Supplemental Files.

### New Species Registration

The following information was supplied regarding the registration of a newly described species: Publication LSID: urn:lsid:zoobank.org:pub:9342C62F-53CF-4A99-9247-E6EDFFBE429A *Icriomastax catia* LSID: urn:lsid:zoobank.org:act:7B009A34-D84B-45CF-8D4D-0FBB8EF2ED3B *Icriomastax coscaroni* LSID: urn:lsid:zoobank.org:act:371FD209-C8E3-426C-8981-02C0BFBFB191 *Icriomastax craigi* LSID: urn:lsid:zoobank.org:act:D4813148-81C7-42BD-AD90-0AA2CF7042EC *Icriomastax lopesae* LSID: urn:lsid:zoobank.org:act:5D894885-8267-4D24-B24A-4B89D499D4A4 *Icriomastax monnei* LSID: urn:lsid:zoobank.org:act:6092A27F-4A88-4EAC-83BE-0C514D347113
