# Supplementary material for: A new perspective on Icriomastax (Diptera: Tipulidae): phylogeny and description of five new species
Source: PeerJ. 2026 Apr 16;14:e21121. doi: 10.7717/peerj.21121 (PMC13092232; doi:10.7717/peerj.21121)
Supplement: Supplemental Information 1 [file peerj-14-21121-s001.docx]

**Appendix 1**: Terminal taxa included in the phylogenetic analysis and corresponding examined material

**Outgroup:**

***Tipula* (*Tipula*) *oleracea* Linnaeus, 1758:** Characters coded exclusively based on information and images available in the literature (Theowald, 1984. Taxonomie, Phylogenie und Biogeographie der Untergattung *Tipula* (*Tipula*) Linnaeus, 1758 (Insecta, Diptera, Tipulidae). Tijdschrift voor Entomologie 127: 33-78; Young, C.W.; Onore, G.; Proano, K. 2000. First occurrence of *Tipula* (*Tipula*) *oleracea* Linnaeus (Diptera: Tipulidae) in the New World, with biological notes. Journal of the Kansas Entomological Society 72: 226-232).

***Holorusia* *hespera* Arnaud and Byers, 1990**: 1♀ (pinned), USA, Colorado, Mesa County, Colo Nat. Mon., 4660’, VI/15/1963, Col. C. J. McCoy, ANSP, det. J. K. Gelhaus; USA, Idaho,1♂ (pinned), USA, Idaho, Cassia County, Schipper F. C. (camp ground) on Co. Rd. G3 18 mi S, Hansen, Sawtooth National Forest, VII/21/1967, Col. K. M. & W. M. Fender, ANSP (Loan US NMNH), det. J. Gouvêa; 1♀ (pinned), USA, Idaho, Idaho County, Fenn Ranger Station Nez Perce National Forest, near Kooskia, Selway Rd. 223, near Johnson Creek, VI/12/1963, Col. R. W. Portman, ANSP (Loan Univ. Idaho), det. J. Gouvêa (ANSP-ENT-133939); 1♂ 1♀ (pinned), USA, Idaho, Boise County, 3.2km (2mi) E. Banks, VI/20/1977, Col. W. F. Barr & J. Domele, ANSP (Loan Univ. Idaho), det. J. Gouvêa; 1♀ (pinned), USA, Idaho, Elmore County, 11km (7mi) E. Featherville, VIII/12/1982, Col. J. McCaffrey, ANSP (Loan Univ. Idaho), det. J. Gouvêa; 1♀ (pinned), USA, Oregon, Eagle Creek, VIII/2/1921, Col. A. L. Melander, NMNH, det. C. P. Alexander; 1♂ (pinned), USA, Oregon, Corvallis, VI/11/1922, Col. A. L. Lovett, NMNH, det. C. P. Alexander; 1♀ (pinned), USA, Oregon, Hood River, I/7/1917, Col. Childs, NMNH, det. J. K. Gelhaus; 1♂ (pinned), USA, Oregon, Hood River, NMNH; 1? (pinned) ,USA, Oregon, Hood River, II/6/1917, NMNH; 1♀ (pinned), USA, Oregon, Cascadia, V/15/1924, Col. H. A. Scullen, NMNH; 1♀ (pinned), USA, Oregon, Cascadia, VIII/10/1924, Col. H. A. Scullen, NMNH; 1♂ (pinned), USA, Oregon, Independence, VI/30/1934, Col. N. P. Larson, NMNH; 3♂ (pinned), USA, Oregon, Corvallis, V/14/1934, Col. N. P. Larson, NMNH; 1♂ (pinned), USA, Oregon, Corvallis, V/9/1934, Col. N. P. Larson, NMNH; 2♂ (pinned), USA, Oregon, Corvallis, V/10/1934, Col. N. P. Larson, NMNH; 1♂ (pinned), USA, Oregon, Corvallis, V/11/1934, Col. N. P. Larson, NMNH, det. J. K. Gelhaus; 1♀ (pinned), USA, Oregon, Corvallis, V/11/1934, Col. N. P. Larson, NMNH; 1♂ (pinned), USA, Oregon, Corvallis, V/13/1934, Col. N. P. Larson, NMNH; 1♀ (pinned), USA, Oregon, Corvallis, VIII/6/1933, Col. J. Schuh, NMNH; 1♂ (pinned), USA, Oregon, McMinnville, VI/25/1946, Col. Macnab, NMNH; 1♂ (pinned), USA, Oregon, McMinnville, VII/15/1945, Col. Fender, NMNH, det. J. K. Gelhaus; 1? (pinned), USA, Oregon, Hood River, V/27/1941, NMNH; 1? (pinned), USA, Oregon, Summer Lake, VI/16/1938, Col. K. Grey & J. Schuh, NMNH; 1♂ (pinned), USA, Oregon, Alsea Creek, V/23/1931, Col. H. A. Scullen, NMNH, det J. K. Gelhaus; 3♂ (pinned) 3♀ (pinned) 1? (pinned), USA, Oregon, Alsea Creek, V/23/1931, Col. H. A. Scullen, NMNH; 1♂ (pinned), USA, Oregon, Collection J. M. Aldrich, #119; 2♂ (pinned) 1♀ (pinned), USA, Oregon, Prospect, V/29/1922, Col. H. G. Dyar, NMNH; 1♂ (pinned), USA, Oregon, Prospect, V/23/1924, Col. H. G. Dyar, NMNH; 1♂ (pinned) 3♀ (pinned) 1? (pinned), USA, Oregon, Prospect, VI/1922, Col. Heston Grieve, NMNH; 1♀ (pinned), USA, Oregon, Curry County, Brookings, VII/20/1977, Col. D. K. Faulkner, ANSP (Loan San Diego NHM), det. J. Gouvêa; 1 ♀ (pinned), USA, Oregon, Multnomah County, Portland, VI/20/1969, Col. R. W. Portman, ANSP (Loan Univ. Idaho), det. J. Gouvêa; 1♀ (pinned), Oregon, Morrow County, Willow Creek, 24 km SE Heppner, 3500', VII/4/1976, Col. John E. Rawlins, CMNH, det. C. Young (CMNH-20,426); 1♂ (pinned), Oregon, Morrow County, Willow Creek, 24 km SE Heppner, 3500', VII/4/1976, Col. John E. Rawlins, CMNH, det. C. Young (CMNH-20,370); 1♀ (pinned), Oregon, Benton County, Corvallie, VII/21/1976, Col. John E. Rawlins, CMNH, det. C. Young (CMNH-20,525); 2♂ (pinned), USA, Washington, Seatlle, NMNH; 1♂ (pinned), USA, Washington, Seatlle, 1937, NMNH, det. J. K. Gelhaus; 1♀ (pinned), USA, Washington, Seatlle, 1937, NMNH; 3♀ (pinned), USA, Washington, Klickitat County, White Salmon, VIII/1962, Col. J. F. G. Clarke, NMNH; 1♂ (pinned) 2? (pinned), USA, Washington, Asotin, V/19/1923, Col. V. Argo, NMNH; 1♀ (pinned), USA, Washington, Diamond Lake, VI/15/1924, Col. A. L. Melander, NMNH; 1♂ (pinned), USA, Washington, Walla Walla, Mill Creek, VII/2-6/1922, Col. A. L. Melander, NMNH; 1♂ 1♀ (pinned), USA, Washington, Seatlle, King County, Bothell, in house, VI/22/1983, Col. R. Sprague, ANSP (Loan Univ. Idaho), det. J. Gouvêa (ANSP-ENT-133938); 1♂ 1♀ (pinned), USA, Washington, King County, Bothell, in house, in copula, VI/22/1983, Col. R. Sprague, ANSP (Loan Univ. Idaho), det. J. Gouvêa; 1♂ (pinned), USA, California, Lassen Co., Mineral, 4900’, IX/15/1948, Col. Schultz, NMNH; 1♂ (pinned), USA, California, Lassen Co., Mineral, 5000’, IX/8/1948, Col. Lester Bodine, NMNH; 1♀ (pinned), USA, California, Leland Stanford University, IV/10/1915, Col. Bred, NMNH; 1? (pinned), USA, California, Berkeley, VI/1963, NMNH; 1♂ (pinned), USA, California, Berkeley, IV/22/1999, Col. B. M. Marlatt; NMNH; 1? (pinned), USA, California, Pine Canyon WN, VI/28/1924, Col. A. L. Melander, NMNH; 1♂ (pinned) 1? (pinned), USA, California, Big Pine, VIII/15/1938, Col. Tinkham, NMNH; 1♂ (pinned) 2? (pinned), USA, California, Big Pine, VIII/18/1938, Col. Tinkham, NMNH; 1♂ (pinned), USA, California, Felton, Santa Cruz County, V/17-18/1947, NMNH; 1♀ (pinned), USA, California, Ventura County, Ozena Forest, Camp 3200’, VI/15/1963, NMNH, det. J. K. Gelhaus; 1♀ (pinned), USA, California, Ventura County, Ozena Forest, Camp 3200’, VI/16/1963, Col. C. W. Kirkwood; NMNH, det. C. P. Alexander; 1♀ (pinned), USA, California, Trinity County, Claire Engle Lake, VI/24/1967, Col. K. & W. Fender; NMNH, det. C. P. Alexander; 1♂ (pinned), USA, California, Riverside, 1944, NMNH; 1♀ (pinned), USA, California, Riverside, V/20/1951, Col. A. L. Melander, NMNH; 1♂ (pinned), USA, California, San Jacinto Moutains, Hurkey Creek, VI/15/1940, NMNH; 2♂ (pinned), USA, California, Ventura County, Los Padres National Forest, Camp Ozena, VI/21/1963, Col. C. W. Kirkwood, NMNH, det. J. K. Gelhaus; 1♀ (pinned), USA, California, Ventura County, Los Padres National Forest, Camp Ozena, VI/19/1963, Col. C. W. Kirkwood, NMNH; 1♀ (pinned), USA, California, Ventura County, Ozena Forest, Camp 3200’, VI/14/1963, Col. C. P. Alexander, NMNH; 1♂ (pinned), USA, California, Tuolumne County, Pinecrest, VIII/11/1948, Col. P. H. Arnaud Jr., NMNH; 1♂ (pinned) 1♀ (pinned), USA, California, Inyo County, Darwin Falls, VIII/19, Col. Arnold & Nancy Menke, NMNH; 1? (pinned), USA, California, Stanford University, IV/1908, NMNH; 1♀ (pinned), USA, California, Chester, VII/12/1942, Col. D. F. Zamzow; NMNH; 2♂ (pinned), USA, California, Mono Lake, VI/27/1949, Col. Sperry, NMNH; 2♂ (pinned) 3♀ (pinned) 1? (pinned), USA, California, Mono Lake, VII/22/1911, Col. J. M. Aldrich, NMNH; 1♂ (pinned), USA, California, Inyo County, Surprise Canyon, IV/29/1953, Col. R. O. Schuster, NMNH; 2♂ (pinned), USA, California, Humboldt County, Bair’s Rch, Redwood Creek, Col. H. S. Barber, NMNH; 1♂ (pinned), USA, California, Eldorado County, Snowline Camp, VII/4/1948, Col. S. A. Sher, NMNH; 1♀ (pinned), USA, California, Wawona, Yosemite National Park, 4012’, VIII/15/1975, Col. Evoneo Bertor F., NMNH; 1♂ (pinned), USA, California, Sofkstanar, VIII/16/1945, Col. A. L. Melander, NMNH; 1♂ (pinned), USA, California, Barton Flats, VII/10/1946, Col. A. L. Melander, NMNH; 1♀ (pinned), USA, California, Sausalito, XII/1973, Col. J. C. Thompson, NMNH; 1♂ (pinned), USA, California, Big Sur, VI/17/1947, Col. A. L. Melander, NMNH; 1♀ (pinned), USA, California, San Jacinto Moutains, Col. F. Grinnel Jr, NMNH; 1♂ (pinned) 1? (pinned), USA, California, Los Angeles County, April, NMNH; 1♂ (pinned), USA, California, San Bernadino Moutains, Col. J. Grinnel, NMNH; 1♂ (pinned) 1? (pinned), USA, California, NMNH; 1♂ (pinned), USA, California, Pasadena, VI/7/1999, NMNH; 1? (pinned), USA, California, Up. Sta Ana R., IX/1/1947, Col. A. L. Melander, NMNH; 1♂ (pinned), USA, California, Up. Sta Ana River., VII/6/1959, Col. A. L. Melander, NMNH; 1♂ (pinned), USA, California, Up. Sta Ana River., VII/14/1950, Col. A. L. Melander, NMNH; 1? (pinned), USA, California, Kern County, Mount Pinos, VI/1904, Col. F. Grinnel Jr., NMNH; 1♀ (pinned), USA, California, Lake Tahoe, Col. Hubbard & Schwarz, NMNH; 1♂ (pinned), USA, California, Santa Cruz, Accession No 1458, Catal No 57, VI/1929; NMNH; 1♂ (pinned), USA, California, San Andreas, IV/7/1925, Col. C. C. Wilson, NMNH; 1♀ (slide), USA, California, Contra Costa, #482, X/1/1966, Col. Denning, NMNH, det. C. P. Alexander (USNMENT 01222947 as H. grandis); 1♂ (slide), USA, California, Inyo County, Surprise Canyon, #482, IV/29/1953, Col. R. O. Schster, NMNH, det. C. P. Alexander (USNMENT 01222923 as H. rubiginosa); 1♂ (pinned), USA, California, Nevada Co., Chicago Park. Oak-pine woodland, #285, VII/31-VIII/1983, ANSP, det. Jon K. Gelhaus; 1♂ (pinned), USA, California, Stanford University, IV/1990, ANSP, det. Jon K. Gelhaus; 1♂ (pinned), USA, California, Stanford University, IV/20/1908, ANSP, det. Jon K. Gelhaus; 1♂ 1♀ (pinned), USA, California, Milpitas, IX/21/1911, ANSP, det. J. Gouvêa; 1♂ (pinned), USA, California, Sonoma, ANSP, det. J. Gouvêa; 1♂ (pinned), USA, California, San Diego Co., Anza-Borrego Desert St Park, Pena Spring, 6,4km of Ranchita, 1036m, #348, IV/23/1986, Col. Jon K. Gelhaus, ANSP, det. Jon K. Gelhaus; 1♀ (pinned), USA, California, San Diego County, Campo, IX/12/1922, Col. M. Hebard, ANSP, det. Jon K. Gelhaus; 3♂ (pinned), USA, California, Nevada County, Chicago Park. Oak-pine woodland, #285, VII/31-VIII/1983, Col. Jon K. Gelhaus, ANSP, det. Jon K. Gelhaus; 1♂ (pinned), USA, California, Nevada County, Chicago Park. 705m, VII/17/1986, Col. Jon K. Gelhaus, ANSP, det. Jon K. Gelhaus; 2♀ (pinned), USA, California, Turlock Lake, St. Cmp.grd. Stanislaus Co. At yellow light in restroom, IX/22/1977, Col. Jon K. Gelhaus, ANSP, det. Jon K. Gelhaus; 1? (pinned), USA, California, Wild Rose Canyon Spring Paivamint Orange-Inyo Co. 3617ft, IX/6/1922, ANSP, det. Jon K. Gelhaus; 1♂ (pinned), USA, California, St. Clara County, Milpitas, VI/1912, ANSP; 1♀ (pinned), USA, California, Plumas County, Butterfly Valley, 1097m, VIII/20/1983, Col. T. L. McCabe, ANSP, det. S. Teale; 1♂ (pinned), USA, California, El Dorado County, #6, American River, 11.4mi W. of Kyburz, 3040ft, VI/9/1976, Col. G. W. Byers & C. W. Young, ANSP, det. S. Teale; 2♂ (pinned), USA, California, Freno County, Pine Ridge, 5000’, 6mi S Shaver Lake, V/14/1984, Col. W. F. Peregrin, ANSP (Loan DFA Dept. Food Agrc), det. J. Gouvêa; 1♀ (pinned), USA, California, Freno County, Tollhouse Grade, 4000’, VI/15/1995, Col. W & J. Thatcher, ANSP (Loan DFA Dept. Food Agrc), det. J. Gouvêa; 2♂ 1♀ (pinned), USA, California, Inyo County, Batchelder Spring, VII/11/1967, Col. R. O. Schuster, ANSP (Loan Univ. Calif. Davis), det. J. Gouvêa; 1♂ 1♀ (pinned), USA, California, Lassen County, Hallelujah Jct. 1440m, VI/21-25/1982, Col. L. D. French, ANSP (Loan Univ. Calif. Davis), det. J. Gouvêa; 1♂ 1♀ (pinned), USA, California, Lassen County, Hallelujah Jct. 1440m, VII/11/1967, Col. R. O. Schuster, ANSP (Loan Univ. Calif. Davis), det. J. Gouvêa; 1♀ (pinned), USA, California, Santa Cruz County, Santa Cruz, V-VI/1969, Col. S. F. Bailey, ANSP (Loan Univ. Calif. Davis), det. J. Gouvêa; 1♀ (pinned), USA, California, Shasta Mountain, Forest pond, VI/1968, Col. F. E. McGowan, ANSP (Loan Univ. Calif. Davis), det. J. Gouvêa; 1♂ (pinned), USA, California, Shaste County, Bateman Rd nr Whitmore, 3100’, Collected at a blacklight, V/28/1976, Col. R. W. Brooks, ANSP (Loan Univ. Calif. Davis), det. J. Gouvêa; 1♀ (pinned), USA, California, Nevada County, Forest Hill, V/20/1967, Col. M. Boussy, ANSP (Loan Univ. Calif. Davis), det. J. Gouvêa; 1♀ (pinned), USA, California, Yolo County, Putah Canyon, VII/10/1970, ANSP (Loan Univ. Calif. Davis), det. J. Gouvêa; 1♂ (pinned), USA, California, Nevada County, Sagehen Creek, VII/21/1976, Col. G. M. Street, ANSP (Loan Univ. Calif. Davis), det. J. Gouvêa; 1♀ (pinned), USA, California, Nevada County, Sagehen Creek, VII/5/1972, Col. S. K. Ault, ANSP (Loan Univ. Calif. Davis), det. J. Gouvêa; 1♀ (pinned), USA, California, Marin County, Hicks Mountain, VI/21/1959, Col. J. S. Buckett, ANSP (Loan Univ. Calif. Davis), det. J. Gouvêa; 3♀ (pinned), USA, California, San Diego County, Boulevard-Manzanita, IX/17/1979, Col. R. Messner, ANSP (Loan San Diego NHM), det. J. Gouvêa; 2♀ (pinned), USA, California, San Diego County, Boulevard-Manzanita, IX/25/1979, Col. R. Messner, ANSP (Loan San Diego NHM), det. J. Gouvêa; 1♀ (pinned), USA, California, San Diego County, Boulevard-Manzanita, IX/26/1979, Col. R. Messner, ANSP (Loan San Diego NHM), det. J. Gouvêa; 1♂ (pinned), USA, California, San Diego County, Boulevard-Manzanita, VII/23/1979, Col. R. Messner, ANSP (Loan San Diego NHM), det. J. Gouvêa; 1♀ (pinned), USA, California, San Diego County, Escondido, IV/29/1980, Col. S. Hutchison, ANSP (Loan San Diego NHM), det. J. Gouvêa; 1♂ 1♀ (pinned), USA, California, San Diego County, IX/7/1976, Col. DKF, ANSP (Loan San Diego NHM), det. J. Gouvêa; 1♂ (pinned), USA, California, San Diego County, VIII/27/1976, Col. DKF, ANSP (Loan San Diego NHM), det. J. Gouvêa; 1♀ (pinned), USA, California, San Diego County, X/2/1976, Col. DKF, ANSP (Loan San Diego NHM), det. J. Gouvêa; 1♀ (pinned), USA, California, Riverside, ANSP (Loan San Diego NHM), det. J. Gouvêa; 1♀ (pinned), USA, California, Banner, ANSP (Loan San Diego NHM), det. J. Gouvêa; 1♂ (pinned), USA, California, San Diego County, Cameron Junction Service Station, VIII/26/1976, Col. DKF, ANSP (Loan San Diego NHM), det. J. Gouvêa; 1♀ (pinned), USA, California, San Diego County, Poway, Green Valey, VI/18/1978, Col. R. Kappel, ANSP (Loan San Diego NHM), det. J. Gouvêa; 1♀ (pinned), USA, California, San Diego County, Poway, VII/12/1978, Col. R. Kappel, ANSP (Loan San Diego NHM), det. J. Gouvêa; 1♀ (pinned), USA, California, San Diego County, Sorrento Valey, X/28/1978, Col. J. W. Brown & D. K. Faulkner, ANSP (Loan San Diego NHM), det. J. Gouvêa; 1♀ (pinned), USA, California, San Diego County, Spring Valey, VI/24/1960, Col. W. Hedges, ANSP (Loan San Diego NHM), det. J. Gouvêa; 1♀ (pinned), USA, California, Fresno County, Wonder Valey, VII/19/2007, Col. N. J. Smith, ANSP (Loan DFA Dept. Food Agrc), det. J. Gouvêa; 1♀ (pinned), USA, California, Fresno County, Cedar Grove, Kings Canyon National Park, IX/17/1985, Col. N. J. Smith, ANSP (Loan DFA Dept. Food Agrc), det. J. Gouvêa; 1♀ (pinned), USA, California, Fresno County, 2 mi N, X/1/1986, Col. N. J. Smith, ANSP (Loan DFA Dept. Food Agrc), det. J. Gouvêa; 1♂ (pinned), USA, California, Fresno County, Pine Ridge 5000’, 6mi S, Shaver Lake, VII/29/1987, Col. N. J. Smith, ANSP (Loan DFA Dept. Food Agrc), det. J. Gouvêa; 1♂ (pinned), USA, California, Merced County, Merced, IX/15/1943, ANSP (Loan Univ. Idaho), det. J. Gouvêa; 1♀ (pinned), USA, California, Madera County, Willow Creek, VII/29/1951, Col. W. C. Day, ANSP (Loan US NMNH), det. J. Gouvêa; 1♂ 1♀ (pinned), USA, California, Plumas County, Keddie, VI/14/1940, Col. Caziert Aitken, ANSP (Loan US NMNH), det. J. Gouvêa; 1♀ (pinned), USA, California, Stanislaus County, Minnier Day Use Area Del Puerto Canyon, #1206, V/10/2009, Col. J. Gelhaus, ANSP, det. Jon K. Gelhaus; 1♀ (pinned), USA, California, Kem County, Alder Creek Cmpgrd, Sequoia National Forest, +- 16km E Glenville on CA #155, 2 km S CA #155; T25SR31B; 26. 1189m, #620, VI/19/1995, Col. J. Gelhaus, ANSP, det. Jon K. Gelhaus; 5♂ 1♀ (pinned), USA, California, Inyo Co.,Toll House Spring, White Mts., Inyo Nat, Forest, 13km NE Big Pine on CA #168, T8S R34E, 24; 1829m, #628, VI/21/1995, Col. J. Gelhaus, ANSP, det. Jon K. Gelhaus; 1♂ (pinned), USA, California, Calaveras County, Sierra Nevada, Stanislaus National Forest, Crescent Cove Picnic Ground 3, 7km W Avery on CA #4; T4NR14E, 26, elev. 940m, #656, VI/27/1995, Col. J. Gelhaus, ANSP, det. Jon K. Gelhaus; 1♂ (pinned), USA, California, Mono County, 8km W Sonora Junction on CA #108, Sierra Nevada, Toiyabe National Forest; T6N, R22E, sec. 23; elev. 1950m, #653, VI/26/1995, Col. J. Gelhaus, ANSP, det. Jon K. Gelhaus; 1♂ 1♀ (pinned), USA, California, Mono County, Swauger CK., Toiyabe National Forest 7.2km NW Bridgeport on US#395 T5N, R24E, sec. 15; elev. 2060m, #650, VI/26/1995, Col. J. Gelhaus, ANSP, det. Jon K. Gelhaus; 1♂ (pinned), USA, California, Deep Ck., Warner Mountains; 5.1km SW of Cedarville on FR#31, Modoc Nat. Forest; T42N, R15E, section 2; 1425m, #665, VI/30/1995, Col. J. Gelhaus, ANSP, det. Jon K. Gelhaus; 8♂ 5♀ (pinned), USA, California, Nevada Co., Chicago Park, el. 705m, #304, VIII/14-15/1984, Col. J. Gelhaus, ANSP, det. Jon K. Gelhaus; 1♂ (pinned), USA, California, San Diego County, San Diego State University, IX/4/1976, Col. Lee Guidry, ANSP (Donated by Ryan Bridge), det. J. Gouvêa; 1♀ (pinned), USA, California, San Diego County, Scissors Crossing, X/22/1977, Col. Lee Guidry, ANSP (Donated by Ryan Bridge), det. J. Gouvêa; 1♀ (pinned), USA, California, Merced County. Merced River at Henderson Park, 1.6km (1mi) E Snelling; el.84m, larvae sieved wet sandy edge of river. J. Gelhaus #287 Gelhaus Rearing #228 Adult emerged ix/11/1983 8:10pm, VIII/10/1983, Col. J. Gelhaus, ANSP (Donated by Ryan Bridge), det. J. Gouvêa (ANSP-ENT-134000); 1♀ (pinned), USA, California, Merced County. Merced River at Henderson Park, 1.6km (1mi) E Snelling; el.84m, larvae sieved wet sandy edge of river. J. Gelhaus #287 Gelhaus Rearing #228 Adult emerged ix/11/1983 8:10pm, VIII/10/1983, Col. J. Gelhaus, ANSP (Donated by Ryan Bridge), det. J. Gouvêa (ANSP-ENT-134001); 1♀ (pinned), USA, California, Nevada County, Chicago Park (Gelhaus Ranch) elev. 705m (2313ft) (T15N R9E sec. 15 SW1/4)J. Gelhaus #304 Gelhaus Rearing #243 Female laid eggs VIII/15/1984 eggs hatched start VIII/22 pm, VIII/14/1984, Col. J. Gelhaus, ANSP (Donated by Ryan Bridge), det. J. Gouvêa (ANSP-ENT-134003); 1♂ (pinned), USA, California, Nevada County, Chicago Park (Gelhaus Ranch) elev. 705m (2313ft) (T15N R9E sec. 15 SW1/4)J. Gelhaus #304 Gelhaus Rearing #243 Female laid eggs VIII/15/1984 eggs hatched start VIII/22 pm, VIII/14/1984, Col. J. Gelhaus, ANSP (Donated by Ryan Bridge), det. J. Gouvêa (ANSP-ENT-134005); 1♂ (pinned), USA, California, Nevada County, Chicago Park (Gelhaus Ranch) elev. 705m (2313ft) (T15N R9E sec. 15 SW1/4)J. Gelhaus #304 Gelhaus Rearing #243 Female laid eggs VIII/15/1984 eggs hatched start VIII/22 pm, VIII/14/1984, Col. J. Gelhaus, ANSP (Donated by Ryan Bridge), det. J. Gouvêa (ANSP-ENT-134002); 1♀ (pinned), USA, California, Merced County. Merced River at Henderson Park, 1.6km (1mi) E Snelling; el.84m, larvae sieved wet sandy edge of river. J. Gelhaus #287 Gelhaus Rearing #228 Adult emerged ix/11/1983 8:10pm, VIII/10/1983, Col. J. Gelhaus, ANSP (Donated by Ryan Bridge), det. J. Gouvêa (ANSP-ENT-134004); 1♂ 1♀ (pinned), USA, California, Humboldt County, 24km (15mi) SE Carlotta, VI/24/1969, Col. W. F. Barr, ANSP, det. J. Gouvêa; 1♀ (pinned), USA, California, Inyo Co. White Mtns., Antelope Spring, 37.33°N 118.09°W elev. 1700m, VI/23/2003, Col. R. W. Garrison, CMNH, det. J. N. Hogue; 1♂ (pinned), USA, Bel Air, West Los Angeles. Carn. Mus. Acc 9410, IV/29/1932, CMNH, det. C. Young (CMNH-20,257); 1♀ (pinned), USA, California, San Mateo, San Mateo County, Herman G. Real Collection. Carn, Mus. Acc. 23489, IV/21/1965, Col. H. G. Real, CMNH, det. C. Young (CMNH-20,306); 1♀ (pinned), USA, California, Nevada County, 8km N Truckee Prosser Creek Lake 1720m., VI/28/1987, Col. J. Rawlins & C. Young, CMNH, det. C. Young (CMNH-15,479); 1♀ (pinned), USA, California, Santa Cruz County, Big Basin Claude D. Hynes Donation 2019, Carnegie Museum Accession, VII/30/1988, Col. S. Balch, CMNH, det. J. Gouvêa (CMNH-38,927); 1♂ (pinned), USA, California, San Luis Obispo County, Claude D. Hynes Donation 2019, Carnegie Museum Accession, VII/5/1988, Col. G. Kenney, CMNH, det. J. Gouvêa (CMNH-38,927); 1♀ (pinned), USA, California, San Luis Obispo County, Claude D. Hynes Donation 2019, Carnegie Museum Accession, V/14/1990, Col. M. Swason, CMNH, det. J. Gouvêa (CMNH-38,927); 1♀ (pinned), USA, California, San Luis Obispo County, Claude D. Hynes Donation 2019, Carnegie Museum Accession, IV/23/1990, Col. Ikenoyana, CMNH, det. J. Gouvêa (CMNH-38,927); 1♂ (pinned), USA, California, San Luis Obispo County, Claude D. Hynes Donation 2019, Carnegie Museum Accession, VII/6/1989, Col. Oberholtzer, CMNH, det. J. Gouvêa (CMNH-38,927); 1♀ (pinned), USA, California, San Luis Obispo County, Claude D. Hynes Donation 2019, Carnegie Museum Accession, VII/25/1988, Col. Clymek, CMNH, det. J. Gouvêa (CMNH-38,927); 1♂ (pinned), USA, California, San Luis Obispo County, Claude D. Hynes Donation 2019, Carnegie Museum Accession, III/8/1997, Col. S. Noyes, CMNH, det. J. Gouvêa (CMNH-38,927); 1♂ (pinned), USA, California, San Luis Obispo County, Cal Poly Campus Claude D. Hynes Donation 2019, Carnegie Museum Accession, V/13/1994, Col. A. Sandoval, CMNH, det. J. Gouvêa (CMNH-38,927); 1♀ (pinned), USA, California, San Luis Obispo County, Cal Poly Campus Claude D. Hynes Donation 2019, Carnegie Museum Accession, IV/10/1992, Col. W. Theis, CMNH, det. J. Gouvêa (CMNH-38,927); 1♀ (pinned), USA, California, San Luis Obispo County, Cal Poly Campus Claude D. Hynes Donation 2019, Carnegie Museum Accession, XI/5/1991, Col. G. Adams, CMNH, det. J. Gouvêa (CMNH-38,927); 1♀ (pinned), USA, California, San Luis Obispo County, Cal Poly Campus Claude D. Hynes Donation 2019, Carnegie Museum Accession, V/5/1992, Col. K. Koepke, CMNH, det. J. Gouvêa (CMNH-38,927); 1♀ (pinned), USA, California, San Luis Obispo County, Cal Poly Campus Claude D. Hynes Donation 2019, Carnegie Museum Accession, IV/19/1992, Col. John Sheder, CMNH, det. J. Gouvêa (CMNH-38,927); 1♂ (pinned), USA, California, San Luis Obispo County, Cal Poly Campus Claude D. Hynes Donation 2019, Carnegie Museum Accession, Col. D. Loukas, CMNH, det. J. Gouvêa (CMNH-38,927); 1♂ (pinned), USA, California, San Luis Obispo County, Cal Poly Campus Claude D. Hynes Donation 2019, Carnegie Museum Accession, V/3/1993, Col. G. Leonard, CMNH, det. J. Gouvêa (CMNH-38,927); 1♂ (pinned), USA, California, San Luis Obispo County, Apartment Claude D. Hynes Donation 2019, Carnegie Museum Accession, IV/26/1992, Col. D. Pratt, CMNH, det. J. Gouvêa (CMNH-38,927); 1♂ (pinned), USA, California, San Luis Obispo County, Cal Poly Campus Claude D. Hynes Donation 2019, Carnegie Museum Accession, IV/23/1992, Col. C. Oswald, CMNH, det. J. Gouvêa (CMNH-38,927); 1♀ (pinned), USA, California, San Luis Obispo County, Cal Poly Campus Claude D. Hynes Donation 2019, Carnegie Museum Accession, V/4/1994, Col. R. Palaikis, CMNH, det. J. Gouvêa (CMNH-38,927); 1♀ (pinned), USA, California, San Luis Obispo County, See Canyon – S. L.O. Claude D. Hynes Donation 2019, Carnegie Museum Accession, XI/10/1991, Col. N. Null, CMNH, det. J. Gouvêa (CMNH-38,927); 1♀ (pinned), USA, California, San Luis Obispo County, Cal Poly Campus, IV/12/1993, Col. M. Crites, CMNH, det. J. Gouvêa (CMNH-38,927); 1♂ (pinned), USA, California, San Luis Obispo County, Atamoca, Claude D. Hynes Donation 2019, VI/29/1989, Col. Centeno, CMNH, det. J. Gouvêa (CMNH-38,927); 1♂ (pinned), USA, California, San Luis Obispo County, Cal Poly, III/5/1988, Col. C. W. Herrala, CMNH, det. J. Gouvêa (CMNH-38,927); 1♀ (pinned), USA, California, San Luis Obispo County, Carnegie Museum Accession, VIII/10/1986, Col. D. C. Smith, CMNH, det. J. Gouvêa (CMNH-36,729); 1♀ (pinned), USA, California, San Luis Obispo County, Carnegie Museum Accession, V/20/1986, Col. B. Holtz, CMNH, det. J. Gouvêa (CMNH-36,729); 1♀ (pinned), USA, California, San Luis Obispo County, Carnegie Museum Accession, V/14/1986, Col. L. Davis, CMNH, det. J. Gouvêa (CMNH-36,729); 1♂ (pinned), USA, California, San Luis Obispo County, Carnegie Museum Accession, V/25/1986, Col. Tama Rachado, CMNH, det. J. Gouvêa (CMNH-36,729); 1♀ (pinned), USA, California, San Luis Obispo County, Carnegie Museum Accession, IX/27/1957, Col. Hynes, CMNH, det. J. Gouvêa (CMNH-36,729); 1♂ (pinned), USA, California, San Luis Obispo County, Carnegie Museum Accession, V/7/1986, Col. M. Suverkrabbe, CMNH, det. J. Gouvêa (CMNH-36,729); 1♂ (pinned), USA, California, San Luis Obispo County, Cambria, Carnegie Museum Accession, IV/6/1963, Col. E. Anderson, CMNH, det. J. Gouvêa (CMNH-36,729); 1♂ (pinned), USA, California, San Luis Obispo County, Carnegie Museum Accession, VIII/10/1988, Col. M. Gasta, CMNH, det. J. Gouvêa (CMNH-38,927); 1♀ (pinned), USA, California, San Luis Obispo County, Carnegie Museum Accession, V/20/1988, Col. M. Achkar, CMNH, det. J. Gouvêa (CMNH-38,927); 1♀ (pinned), USA, California, San Luis Obispo County, Carnegie Museum Accession, IV/18/1988, Col. S. Bigley, CMNH, det. J. Gouvêa (CMNH-38,927); 1♂ (pinned), USA, California, San Luis Obispo County, Carnegie Museum Accession, VII/16/1988, CMNH, det. J. Gouvêa (CMNH-38,927); 1♂ (pinned), USA, California, San Luis Obispo County, Carnegie Museum Accession, V/5/1988, Col. Grannis, CMNH, det. J. Gouvêa (CMNH-38,927); 1♀ (pinned), USA, California, San Luis Obispo County, Carnegie Museum Accession, VII/29/1988, Col. J. Tracy, CMNH, det. J. Gouvêa (CMNH-38,927); 1♀ (pinned), USA, California, San Luis Obispo County, Carnegie Museum Accession, XI/4/1999, Col. L. McDocgall, CMNH, det. J. Gouvêa (CMNH-38,927); 1♀ (pinned), USA, California, San Luis Obispo County, Carnegie Museum Accession, IV/27/1949, Col. R. W. Buchwitz, CMNH, det. J. Gouvêa (CMNH-38,927); 1♀ (pinned), USA, California, San Luis Obispo County, Carnegie Museum Accession, IV/12/1949, Col. R. Harader, CMNH, det. J. Gouvêa (CMNH-38,927); 1♀ (pinned), USA, California, San Luis Obispo County, Arroyo Grande, Carnegie Museum Accession, V/11/1988, Col. H. Hammon, CMNH, det. J. Gouvêa (CMNH-38,927); 1♂ (pinned), USA, Sacramento County, Folsom, 362 Silberhorn Rd., At Light 4852, IV/24/1999, Col. T. J. McNary, CMNH, det. J. Gouvêa (CMNH-446,743); 1♀ (pinned), USA, California, San Luis Obispo County, Cal. Poly, Carnegie Museum Accession, IX/27/1999, Col. Ron Mullisen, CMNH, det. J. Gouvêa (CMNH-38,927); 1♀ (pinned), USA, California, Santa Clara County, Carnegie Museum Accession, IV/30/1988, Col. S. Fontain, CMNH, det. J. Gouvêa (CMNH-38,927); 1♂ (pinned), USA, California, Santa Clara County, Carnegie Museum Accession, IV/20/1988, Col. S. Fontain, CMNH, det. J. Gouvêa (CMNH-38,927); 1♂ (pinned), USA, California, Mendocino County, Van Damme Beach State Park, Carnegie Museum Accession, VIII/30/1961, Col. C. D. Hynes, CMNH, det. J. Gouvêa (CMNH-36,729); 1♀ (pinned), USA, California, Mendocino County, Van Damme Beach State Park, Carnegie Museum Accession, VIII/30/1961, Col. Hynes, CMNH, det. J. Gouvêa (CMNH-36,729); 1♀ (pinned), USA, California, Mendocino County, Van Damme Beach State Park, Carnegie Museum Accession, VIII/30/1961, Col. B. Page, CMNH, det. J. Gouvêa (CMNH-36,729); 1♀ (pinned), USA, California, Monterey County, Elkhonor, Carnegie Museum Accession, VIII/10/1986, Col. Kelden, CMNH, det. J. Gouvêa (CMNH-36,729); 1♀ (pinned), USA, Arizona, S. W. Res. Sta. 5mi, sw. Portal, 5400ft, V/23-IV/5/1967, Col. C. W. Sabrosky, NMNH; 1♂ (pinned), USA, Arizona, Sulphur Draws s. Portal, V/29/1967, Col. C. W. Sabrosky, NMNH; 1♂ (pinned), USA, Arizona, Flagstaff, VI/1949, Col. E. McRee, NMNH; 1♀ (pinned), USA, Arizona, Flagstaff, VI/22/1949, Col. E. McRee, NMNH; 2♀ (pinned), USA, Arizona, Cochise County, Southwestern Research Station, VI/24-25/1958, Col. C. W. Kirkwood, NMNH; 1♂ (pinned) 1♀ (pinned), USA, Arizona, Coconino County, VI/13/1939, Col. Gloyd, NMNH; 12♂ (pinned) 5♀ (pinned), USA, Arizona, Oak Creek Canon, 5180ft, VI/10/1942, Col. C. P. Alexander, NMNH; 2♀ (pinned), USA, Arizona, Oak Creek Canyon, Manzanita Camp, VII/27/1950, NMNH; 1? (pinned), USA, Arizona, San Francisco Peaks, VIII/10/1929, NMNH; 1♀ (pinned), USA, Arizona, Cochise County, Chiricahua Mountains, Southwestern Res. Station, VI/21-23/1958, Col. C. W. Kirkwood, NMNH; 2♂ (pinned), USA, Arizona, Cochise County, Chiricahua Mountains, Southwestern Res. Station, VI/20/1958, Col. C. W. Kirkwood, NMNH; 1♂ (pinned), USA, Arizona, Cochise County, Pinery Cnyon 6000ft Chiricahua Mts, VII/10/1919, Col. Witmer Stone, ANSP, det. Jon K. Gelhaus; 1♂ (pinned), USA, Arizona ,Greenlee County, White mountains, Granville Campground, elev. 2100m Chase Creek, along creek at night, , #600, V/28/1993, Col. Gelhaus, Nelson & Koenig, ANSP, det. Jon K. Gelhaus; 1♂ (pinned), Arizona, Cochise County, Portal. 1600m., V/30/1991, Col. Chen W. Young, CMNH, det. C. Young (CMNH-20,217); 1♂ (pinned), Arizona, Cochise County, Portal. 1600m., H10, V/30/1991, Col. Chen W. Young, CMNH, det. J. Gouvêa (CMNH-17,610); 1♂ (pinned), Arizona, Cochise County, Portal. 1600m., H9, V/30/1991, Col. Chen W. Young, CMNH, det. J. Gouvêa (CMNH-20,516); 1♂ (pinned), Arizona, Graham Co. Pinaleno Mts. 8km WSW Artesia, Noon Creek 1680m., H11, VIII/7-8/1991, Col. M. Daman, R. Davidson, M. Klingler, W. Zanol & J. Rawlins, CMNH, det. J. Gouvêa (CMNH-168,455); 3♂ (pinned), USA, Utah, Zion National Park, alt. 4500ft, VI/22/1942, Col. C. P. Alexander, NMNH; 1♂ (pinned), USA, Utah, Beaver County, Wildcat Valley, 144. Catal. No. 58, NMNH; 2♂ 1♀ 1? (pinned), USA, Utah, Juab Co., Trout Creek, VII/24/1922, Col. Tom Spalding, ANSP, det. G. S. Rogers; 1♂ (pinned), USA, Utah, Juab Co., Trout Creek, VII/24/1922, Col. Tom Spalding, ANSP, det. Jon K. Gelhaus; 2♀, 1? (pinned), USA, Utah, Juab Co., Trout Creek, VII/24/1922, Col. Tom Spalding, ANSP; 1?, USA, Utah, Provo, VII/31/1912, Col. Tom Spalding, ANSP; 3♂ 2♀ (pinned), USA, Nevada, Reno, VI/1911, ANSP; 1♂ 1♀ (pinned), USA, Nevada, Clark County, Willow Ck Cp, Charlston Mountains, VII/14/1977, Col. N. J. Smith, ANSP (Loan Univ. Calif. Davis), det. J. Gouvêa; 5♂ 2♀ (pinned), USA, Nevada, Humboldt County, Pine Forest Range, hillside springs, 6.4km W hwy, 140 +-18.5km S Denio Jct; T45N, R30E, 15; 1545m, #681, VIII/1/1995, Col. J. Gelhaus & Bouchard, ANSP, det. Jon K. Gelhaus; 1♂ (pinned), USA, California, Nevada Co. Chicago Park (Gelhaus Ranch) elev. 705m (2313ft) (T15N R9E sec. 15 SW1/4), #304, VIII/14/1984, Col. J. Gelhaus, ANSP, det. J. Gouvêa (ANSP-ENT-134024); 1♂ (pinned), USA, California, Nevada Co. Chicago Park (Gelhaus Ranch) elev. 705m (2313ft) (T15N R9E sec. 15 SW1/4), #304, VIII/14/1984, Col. J. Gelhaus, ANSP, det. J. Gouvêa (ANSP-ENT-134036); 1♀ (pinned), USA, California, Nevada Co. Chicago Park (Gelhaus Ranch) elev. 705m (2313ft) (T15N R9E sec. 15 SW1/4), #304, VIII/14/1984, Col. J. Gelhaus, ANSP, det. J. Gouvêa (ANSP-ENT-134032); 1♀ (pinned), USA, New Mexico, Grant County, North of Silver City, 2,4mi of North Cherr Creek Camp, VII/7/1969, Col. Koss, McCafferty, Provonsha, NMNH; 1♂, USA, New Mexico, Jemez Mountains, VI/28/1914, ANSP, det. S. A. Teale; 1♂, USA, New Mexico, Jemez Mountains, VII/8/1914, ANSP, det. S. A. Teale; 1♂, USA, New Mexico, Jemez Mountains, VII/16/1914, ANSP; 1♂, USA, New Mexico, Jemez Mountains, VI/4/1919, ANSP; 1?, USA, New Mexico, Jemez Mountains, VI/6/1919, ANSP; 1♂, USA, New Mexico, Fort Wingate, VII/23/1908, Col. John Woodgate, ANSP; 1♀ (pinned), USA, New Mexico, Catron Co. Pueblo Creek & Park Campgd. Gila National Forest, 27km S. Luna elev. 2100m. at light, #603, V/29/1993, Col. Gelhaus, Nelson & Koenig, ANSP, det. Jon K. Gelhaus; 2♀ (pinned), USA, Alaska, Mckinley Park, F. Morand, NMNH; 1♀ (pinned), Canada, BC, Vancouver, VII/1929, Col. G. J. Spencer; NMNH; 1♂ (pinned), Canada, BC, Vancouver, VI/20/1930, Col. G. J. Spencer; NMNH; 1♂ (pinned), Canada, BC, Vancouver, U. B. C. Campus, VII/4-9/1988, Col. W.E. Steiner, NMNH; 1♂ (pinned)1♀ (pinned), Canada, BC, Lilloock, VII/12/1939, Col. C. R. Phair, NMNH; 1♀ (pinned), Canada, BC, Vancouver, VII/28/1931, Col. H. B. Leech, NMNH; 1♀ (pinned), Canada, BC, VII/2/1931, Col. H. B. Leech, NMNH; 1♀ (pinned), Canada, BC, Vancouver, Point Grey, VI/17/1960, Col. A. P. Gupta, ANSP (Loan Univ. Idaho), det. J. Gouvêa;

***Holorusia* *laticellula* (Alexander, 1949)**: 1♂ (pinned), China, Kwangtung, VI/3/1938, NMNH, det. C. P. Alexander (USNMENT 0135153 [holotype]).

***Holorusia* *makara* (Alexander, 1967)**: 1♂ (pinned), India, Assam, Manipur, Lakhan-Khuman, 3000’, VII/26/1960, Col. Schmid, NMNH, det. C. P. Alexander (USNMENT 01351098 [holotype]); 1♂ (slide), India, Assam, Manipur, Lakhan-Khuman, 3000’, #11227, VII/26/1960, Col. Schmid, NMNH, det. C. P. Alexander (USNMENT 01222732 [holotype]); 1♂ (slide), India, Assam, Manipur, Lakhan-Khuman, 3000’, #11227, VII/26/1960, Col. Schmid, NMNH, det. C. P. Alexander (USNMENT 01222733 [holotype]).

***Holorusia* *mara* (Alexander, 1953)**: 1♂ (pinned), India, Assam, Meghalaya, Khasi, Cherrapunji, IV/8/1935, Col. S. Sircar, NMNH, det. C. P. Alexander (USNMENT 01351035 [holotype]); 1♂ (slide), India, Assam, Meghalaya, Khasi, Cherrapunji, #9583, IV/8/1935, Col. S. Sircar, NMNH, det. C. P. Alexander (USNMENT 01222734 [holotype]); 1♂ (slide), India, Assam, Meghalaya, Khasi, Cherrapunji, #9583, IV/8/1935, Col. S. Sircar, NMNH, det. C. P. Alexander (USNMENT 01222725 [holotype]).

***Holorusia* *similis* (Edwards, 1921)**: 1♀ (pinned), China, Taiwan, Taipei Mozar, V/10/1985, Col. C. Y. Lee, CMNH, det. C. Young (CMNH-20,535); 1♀ (pinned), China, Taiwan, Taipei Mozar, V/10/1985, Col. C. Y. Lee, CMNH, det. C. Young (CMNH-20,272); 1♀ (pinned), China, Taiwan, Taipei Mozar, IV/25/1985, Col. W. K. Shu, CMNH, det. C. Young (CMNH-20,507); 1♂ (pinned), China, Taiwan, Taipei Mozar, V/10/1985, Col. C. Y. Lee, CMNH, det. C. Young (CMNH-20,484); 1♀ (pinned), China, Guizhou Province, Chishui, Soulou Res., VII/15-16/2001, Col. D. Yang & C. Young, CMNH, det. C. Young; 1♂ (pinned), China, Guizhou Province, Chishui, Xishui, Shanchahe, 1000m, H9, VII/14/2001, Col. D. Yang & C. Young, CMNH, det. C. Young (CMNH-451,360); 1♂ 1♀ (pinned), Taiwan, Taipei, SanSia, ChienAn, V/7/2007, Col. Ling-Chu Lin, CMNH, det. J. Gouvêa; 1♂ 1♀ (pinned), Taiwan, Taipei, SanSia, ChienAn, V/24/2007, Col. Ling-Chu Lin, CMNH, det. J. Gouvêa; 1♀ (pinned), Taiwan, Taipei, Sijhih Dajian Mts. 220m 25-03-14N 121-40-22E, V/6/2007, Col. Ling-Chu Lin, CMNH, det. J. Gouvêa; 2♂ (pinned), Taiwan, Taipei, Tu Cheng, Kan Lu Temple, IV/27/2007, Col. Ling-Chu Lin, CMNH, det. J. Gouvêa; 1♂ (pinned), Taiwan, Taipei, Tu Cheng, Kan Lu Temple, VII/13/2007, Col. Ling-Chu Lin, CMNH, det. J. Gouvêa; 1♂ (pinned), Taiwan, Taipei, YangmingSan Cgung-Ping Trail 476m 25-09-05N 121-32-45E, VI/13/2007, Col. Ling-Chu Lin, CMNH, det. J. Gouvêa; 1♂ (pinned), Taiwan, Taipei, YangmingSan Cgung-Ping Trail 476m 25-09-05N 121-32-45E, VI/21/2007, Col. Ling-Chu Lin, CMNH, det. J. Gouvêa; 1♂ 1♀ (pinned), Taiwan, Kaohsiung, Ten-chih, 1550m, 23-04-03N 120-45-13E, VIII/23/1996, Col. Chen Wen Young, CMNH, det. J. Gouvêa; 1♂ (pinned), Taiwan, Pintung County, Manzhou Twp. Lanren Road 22.0410N 120.8382E 107m 1620-000027, H10, V/23/2011, Col. Yu-Da Chuan, CMNH, det. J. Gouvêa (CMNH576,512).

***Holorusia* *siva* (Alexander, 1950)**: 1♂ (pinned), India, Nilgiris, Mango Range, 3800’, V/20/1949, Col. S. Nathan, NMNH, det. C. P. Alexander (USNMENT 01351090 [holotype]); 1♂ (pinned), India, Nilgiris, Cherangode, 3500’, X/1950, Col. Nathan, NMNH, det. C. P. Alexander; 1♀ (pinned), India, Nilgiris, Cherangode, 3500’, IX/1950, Col. Nathan, NMNH, det. C. P. Alexander (USNMENT 01239020); 1♂ (pinned), India, Nilgiris, Cherangode, 3500’, IX/1950, Col. Nathan, NMNH, det. C. P. Alexander; 1♂ (slide), India, Nilgiris, Mango Range, 3800’, #9043, V/20/1949, Col. S. Nathan, NMNH, det. C. P. Alexander (USNMENT 01222804 [holotype]).

***Holorusia* *viettei* (Alexander, 1957)**: 1♂ (pinned), Principe, Roca, Porto Real, 60m, VI/29-30/1956, Col. Viette, NMNH, det. C. P. Alexander (USNMENT 01222884 [paratype]); 1♂ (SLIDE), Principe, Roca, Porto Real, 60m, #10226, VI/29-30/1956, Col. Viette, NMNH, det. C. P. Alexander (USNMENT 01222904 [paratype]).

***Holorusia* *vinsoniana* (Alexander, 1956)**: 1♂ (pinned), Mauritius, Mount Cocotte, X/9/1954, Col. J. Vinson, NMNH, det. C. P. Alexander (USNMENT 01222875 [paratype]); 1♂ (pinned), Mauritius, Mount Cocotte, X/9/1954, Col. J. Vinson, NMNH, det. C. P. Alexander; 1♂ (pinned), Mauritius, Mount Cocotte, IV/10/1954, Col. J. Vinson, NMNH, det. C. P. Alexander; 1♂ (slide), Mauritius, Mount Cocotte, # 10819, IV/10/1954, Col. J. Vinson, NMNH, det. C. P. Alexander (USNMENT 01222895 [paratype]).

***Ischnotoma* (*Ischnotoma*) *araucana* (Alexander, 1929)**: 1♂ (pinned) 1♀ (pinned), Chile, Curacautin, XII/4/1950, Col. Peña, NMNH, det. C. P. Alexander; 1♀ (pinned), Chile, Malleco, Araucania, Las Raíces, 1100m, XII/19-22/1976, Col. L. Peña, NMNH, det. C. P. Alexander; 1♀ (pinned), Chile, Arauco, Nahuelbuta, II/2/1953, Col. Luis E. Peña, NMNH, det. C. P. Alexander (USNMENT 01240555); 1♀ (pinned), Chile, Arauco, Nahuelbuta, II/2/1953, Col. Luis E. Peña, NMNH, det. C. P. Alexander; 1♂ (pinned), Chile, Curacautin, XII/4/1950, Col. Peña, NMNH, det. C. P. Alexander (USNMENT 01240556); 1♂ (slide), Chile, Malleco, Curacautin, 400m, #3379, XII/14/1950, Col. L. S. Peña, NMNH, det. C. P. Alexander (USNMENT 01222979); 1♂ (SLIDE), Chile, Ancua, #3379, XII/17-19/1926, Col. F. & M. Edwards, NMNH, det. C. P. Alexander (USNMENT 01222976); 1♂ (pinned), Chile, Los Lagos Region, Valvdivia Prov.,La Union; Las Trancas, I/23-29/1995, Col. Luis E. Peña, ANSP, det. J. Gouvêa (ANSP-ENT-133997); 1♀ (pinned), Chile, Los Lagos Region, Llanquihue Province, Correntoso, , I/29-31/1995, Col. Luis E. Peña, ANSP, det. J. Gouvêa (ANSP-ENT-133996); 1♀ (pinned), Chile, Bío-Bío Reg;, Malleco Province; 'Rio Blanco' ([E of] Curacautín); [= Termas (Banos) de Rio Blanco], II/1995, Col. Luis E. Peña, ANSP, det. J. Gouvêa (ANSP-ENT-133995); 1♀ (pinned), Chile, La Araucanía Reg.; Malleco Province; Lonquimay, XII/1994, Col. Luis E. Peña, ANSP, det. J. Gouvêa (ANSP-ENT-133994).

***Ischnotoma* (*Ischnotoma*) *concinna* (Philippi, 1866)**: 1♀ (pinned), Chile, Cautin, Villarrica, 30Km NE, I/1-30/1965, Col. Peña, NMNH, det. C. P. Alexander (USNMENT 01222557); 1? (pinned), Chile, Cautin, Villarrica, 30Km NE, I/1-30/1965, Col. Peña, NMNH, det. C. P. Alexander; 1? (pinned), Chile, Curanipe, XII/4/1953, Col. Peña, NMNH, det. C. P. Alexander (USNMENT 01222558); 1♀ (pinned), Chile, Ñuble, Las Trancas, 1300m, #50, XII/14-15/1976, Col. Luis Peña, NMNH, det. C. P. Alexander; 1♂ (slide), Chile, Cautin, Villarrica, 30Km NE, #13089, I/1-30/1965, Col. Peña, NMNH, det. C. P. Alexander (USNMENT 01222717); 1♂ (slide), Chile, Maule, Curanipe, #13089, XII/4/1953, Col. L. E. Peña, NMNH, det. C. P. Alexander (USNMENT 01222715); 1♀ (pinned), Chile, La Araucanía Reg.; Malleco Province; Lonquimay, XII/1994, Col. Luis E. Peña, ANSP, det. J. Gouvêa (ANSP-ENT-133992); 1♀ (pinned), Chile, La Araucanía Reg.; Malleco Province; Lonquimay, XII/1994, Col. Luis E. Peña, ANSP, det. J. Gouvêa (ANSP-ENT-133998).

***Ischnotoma* (*Ischnotoma*) *decorata* (Philippi, 1866)**: 1♂ (pinned), Chile, Santiago, Marga Marga, X/7/1927, Col. Jaffuel & Pirion, NMNH, det. J. Gouvêa (USNMENT 01222885); 1♂ (pinned), Chile, Chiloe, Ancud, XII/17-19/1926, Col. R. C. Shannon, NMNH; 1♂ (pinned), Chile, Valle Ramon, 1000m, II/10-21/1955, NMNH; 1♀ (pinned), Chile, Panguipulli, XII/23/1923, Col. Jaffuel & Pirion, NMNH, det. J. Gouvêa (USNMENT 01222894); 1♂ (pinned) 1♀ (pinned) 1? (pinned), Chile, Marga Marga, II/1933, Col. Pirion, NMNH; 1♂ (pinned), Chile. Pailahueque, I/1928, Col. A. Pirion, NMNH; 1♀ (pinned), Chile, Panguipulli, XII/23/1923, Col. Jaffuel & Pirion, NMNH, det. C. P. Alexander; 1♂ (pinned) 1♀ (pinned), Chile, Marga Marga, X/17/1927, Col. Jaffuel & Pirion, NMNH, det. C. P. Alexander; 1♂ (pinned), Chile, Marga Marga, X/13/1927, Col. Jaffuel & Pirion, NMNH, det. C. P. Alexander; 1♂ (pinned), Chile, Marga Marga, X/14/1927, Col. Jaffuel & Pirion, NMNH, det. C. P. Alexander; 1♂ (pinned), Chile, Marga Marga, X/12/1927, Col. Jaffuel & Pirion, NMNH, det. C. P. Alexander; 1♀ (pinned), Chile, Valdivia, Panguipulli, I/13/1924, Col. Alfredo Faz, NMNH, det. C. P. Alexander; 1♀ (pinned), Chile, Marga Marga, X/14/1927, Col. Jaffuel & Pirion, NMNH, det. C. P. Alexander; 1♂ (slide), Chile, Marga Marga, Valparaiso, #13090, I/1933, Col. A. Pirion, NMNH, det. C. P. Alexander (USNMENT 01222716); 1♂ (slide), Chile, Marga Marga, #13090, I/1933, Col. A. Pirion, NMNH, det. C. P. Alexander (USNMENT 01222719); 1♂ (slide), Chile, Marga Marga, #13090, I/1933, Col. A. Pirion, NMNH, det. C. P. Alexander (USNMENT 01222719); 1♂ (slide), Chile, Valle Ramon, 1000m, #13090, II/10-21/1955, NMNH, det. C. P. Alexander (USNMENT 01222977); 1♀ (pinned), Chile, Parque Nacional Laguna del Laja, 1100m, I/26-29/1991, Col. P. Oosterbroek & F. M. Hartveld, NATURALIS; 1♂ (pinned), Chile, Ensenada, 50km E Puerto Varas, 0-50m, I/8-15/1991, Col. P. Oosterbroek & F. M. Hartveld, NATURALIS.

***Ischnotoma* (*Ischnotoma*) *delpontei* (Alexander, 1926)**: 1♂ (pinned), Chile, Ensenada, Type No. 55922 USNM, XII/14/1926, Col. E. Del Ponte, NMNH, det. C. P. Alexander [holotype]; 1♀ (pinned), Chile, Puerto Montt, #5592, XII/24/1926, Col. R. C. Shannon, NMNH, det. C. P. Alexander (USNMENT 01222401 [paratype]); 1♂ (pinned), Chile, Casa Panque, #5592, XII/10/1926, Col. R. C. Shannon, NMNH, det. C. P. Alexander (USNMENT 01222886 [paratype]); 1♀ (pinned), Chile, R. Blanco, III/5-27/1951, Col. Peña, NMNH, det. C. P. Alexander; 1♀ (pinned), Chile, Malleco, Nahuelbuta, Cabreira, #69, I/15-20/1977, Col. Luis Peña, NMNH, det. C. P. Alexander (USNMENT 01222559); 1♀ (pinned), Chile, Chiloé Island, Dalcahue, II/10-12/1954, Col. Peña, NMNH, det. C. P. Alexander; 1? (pinned), Argentina, Lago Nonthué, XII/18/1950, Col. Schachovsky, NMNH, det. C. P. Alexander (USNMENT 01222546); 1♀ (pinned), Argentina, Lago Nonthué, I/5/1951, Col. Schachovsky, NMNH, det. C. P. Alexander (USNMENT 01222550); 1♂ (pinned), Argentina, Neuquén, Lago Lacar, 650m, I/12/1954, Col. Schachovsky, NMNH, det. C. P. Alexander; 1♂ (pinned), Argentina, Neuquén, Lago Lacar, 650m, II/16/1954, Col. Schachovsky, NMNH, det. C. P. Alexander; 1♂ (slide), Chile, Ensenada, #3938, XII/14/1926, Col. E. Del Ponte, NMNH, det. C. P. Alexander (USNMENT 01222481 [holotype]); 1♂ (slide), Chile, Casa Pangue, #3938, XII/4-10/1926, Col. F. M. Edwards, NMNH, det. C. P. Alexander (USNMENT 01222974 [paratype]); 1♂ (slide), Chile, Malleco, Nahnelbuta, Cabreria, 1100m, #3938, I/15-20/1977, Col. Luis Peña, NMNH, det. C. P. Alexander (USNMENT 01222971).

***Ischnotoma* (*Ischnotoma*) *eburnea* (Walker, 1848)**: 1♀ (pinned), Australia, New South Wales, Dorrigo, IX/13/1931, Col. Herm, NMNH, det C. P. Alexander (USNMENT 01222551); 1? (pinned), Australia, New South Wales, Dorrigo, II/10/1931, Col. Herm, NMNH, det C. P. Alexander; 1♂ (slide), Australia, New South Wales, Dorrigo, #1691, IX/13/1931, Col. Herm, NMNH, det C. P. Alexander (USNMENT 01222964); 2♂ 1♀ (pinned), Australia, Tasmania, Univ. Tasmania Hobart, XII/22/1975, Col. O. Sheilds, ANSP (Loan Univ. Calif. Davis), det. J. Gouvêa. As *serricornis*: 1♂ (slide), Australia, Tasmania, Mount Wellington, #1691, Col. A. M. Lea, NMNH, det C. P. Alexander (USNMENT 01222914); 1♂ (slide), Australia, Tasmania, Mount Wellington, #1691, I/1920, Col. A. M. Lea, NMNH, det C. P. Alexander (USNMENT 01222919).

***Ischnotoma* (*Ischnotoma*) *episema* Alexander, 1924**: 1♀ (pinned), Australia, Mountain Kosciusko, XII/7/1923, Col. Goldfinch, NMNH, det C. P. Alexander (USNMENT 01222552); 1♀ (slide), Australia, New South Wales, Mountain Kosciusko, #2698, XII/7/1922, Col. Goldfinch, NMNH, det C. P. Alexander (USNMENT 01222965).

***Ischnotoma* (*Ischnotoma*) *fagetorum* (Alexander, 1929)**: 1♂ (pinned), Argentina, Correntoso, #55924, XI/14/1926, Col. R. C. Shannon, NMNH, det C. P. Alexander [paratype]; 1♂ (slide), Argentina, Bariloche, Patagonia, XI/28-XII/1/1926, Col. F. e M. Edwards, NMNH, det C. P. Alexander (USNMENT 01222960 [paratype]).

***Ischnotoma* (*Ischnotoma*) *fuscostigmosa* (Alexander, 1929)**: 1? (pinned), Chile, Bio Bio, I/1928, Col. Pirion, NMNH, det C. P. Alexander (USNMENT 01240568 [paratype]); 1♂ (pinned), Chile, Pailanhueque, I/1928, Col. Pirion, NMNH, det C. P. Alexander (USNMENT [paratype]); 1? (pinned), Chile, Chiloé Island, Aucar, I/1915, Col. Luis Peña Guzman, NMNH, det C. P. Alexander; 1♂ (pinned), Chile, Chiloé Island, Dalcahue, II/10-12/1954, Col. Peña, NMNH, det C. P. Alexander (USNMENT 01222547); 1? (pinned), Chile, Chiloé Island, Dalcahue, II/10-12/1954, Col. Peña, NMNH, det C. P. Alexander; 1? (pinned), Argentina, Lago Nonthué, II/6/1951, Col. Schachovskoy, NMNH, det C. P. Alexander; 1♂ (slide), Chile, Chiloé Island, Dalcahue, #3939, II/10-12/1954, Col. Peña, NMNH, det C. P. Alexander (USNMENT 01222724); 1♀ (slide), Argentina, Lago Nonthué, #3939, II/6/1951, Col. Schachovskoy, NMNH, det C. P. Alexander (USNMENT 01222723); 1♀ (pinned), Chile, Aisen, Tranquilo, S. Murta, H1, II/6-7/1990, Col. Luis E. Peña, CMNH, det. J. Gouvêa (CMNH-576,407); 1♀ (pinned), Chile, Aisen, Tranquilo, S. Murta, H2, II/6-7/1990, Col. Luis E. Peña, CMNH, det. J. Gouvêa (CMNH-576,408); 1♂ (pinned), Chile, Aisen, Tranquilo, S. Murta, G10, II/6-7/1990, Col. Luis E. Peña, CMNH, det. J. Gouvêa (CMNH-576,404); 1? (pinned), Chile, Pehuenco, Malleco, 1150m, XII/13-17/1990, Col. L. Peña, CMNH, det. J. Gouvêa (CMNH-20,545).

***Ischnotoma* (*Ischnotoma*) *goldfinchi* Alexander, 1924**: 1♀ (pinned), Australia, Mountain Kosciusko, XII/7/1922, Col. Goldfinch, NMNH, det C. P. Alexander (USNMENT 01240548 [allotype]); 1♂ (slide), Australia, New South Wales, Mountain Kosciusko, #2790, XII/7/1922, Col. Goldfinch, NMNH, det C. P. Alexander (USNMENT 01222460 [holotype]); 1♀ (slide), Australia, New South Wales, Mountain Kosciusko, #2790, XII/7/1922, Col. Goldfinch, NMNH, det C. P. Alexander (USNMENT 01222946 [allotype]).

***Ischnotoma* (*Ischnotoma*) *larotypa* (Alexander, 1929)**: 1♀ (pinned), Chile, Chiloé, Castro, XII/19/1926, Col. R. Shannon & E. Shannon, NMNH, det C. P. Alexander (USNMENT 01222775 [holotype]); 1♂ (pinned), Chile, Malleco, Nahuelbuta, Cabreira, 1100m, I/9-10/1977, Col. L. Peña, NMNH, det C. P. Alexander (USNMENT 01222512); 1♀ (slide), Chile, Chiloé, Castro, #3941, XII/19/1926, Col. R. Shannon & E. Shannon, NMNH, det C. P. Alexander (USNMENT 01222735 [holotype]); 1♂ (slide), Chile, Malleco, Nahuelbuta, Cabreira, 1100m, #3941, I/9-10/1977, Col. L. Peña, NMNH, det C. P. Alexander (USNMENT 01222940).

***Ischnotoma* (*Ischnotoma*) *par* (Walker, 1856)**: 1? (pinned), Australia, Blue Mountains, Health Dept., T.23, IV/3/1927, NMNH, det C. P. Alexander; 1♂ (pinned), Australia, Barrington, SU Zoo Exp., I/1925, NMNH, det C. P. Alexander (USNMENT 01222513); 1? (pinned), Australia, Barrington, SU Zoo Exp., I/1925, NMNH, det C. P. Alexander; 1♂ (pinned), Australia, Victoria, Col. Fernshaw, NMNH, det C. P. Alexander; 1♀ (pinned), Australia, Victoria, II/25/1914, NMNH, det C. P. Alexander; 1♂ (pinned), Australia, Victoria, NMNH, det C. P. Alexander (USNMENT 01222514); 1♀ (slide), Australia, Victoria, Millgrove, #1689, IV/1/1928, Col. F. E. Wilson, NMNH, det C. P. Alexander (USNMENT 01222933); 1♂ (slide), Australia, Blue Mountains, #1689, III/4/1922, Col. E. W. Ferguson, NMNH, det C. P. Alexander (USNMENT 01222925); 1♂ (pinned), Australia, North South Wales, Dumaresq Dam 3.5mi N Armidale, III/1978, Col. D. Katz, ANSP (Loan Univ. Calif., Davis), det. J. Gouvêa.

***Ischnotoma* (*Ischnotoma*) *penai* (Alexander, 1929)**: 1♂ (pinned), Chile, Chiloe Island, Aucar, I/6-15/1952, Col. Peña, NMNH, det C. P. Alexander (USNMENT 01222429 [holotype of *Holorusia guzmani*]); 1♂ (slide), Chile, Chiloe Island, Aucar, #7551, I/6-15/1952, Col. Peña, NMNH, det C. P. Alexander (USNMENT 01222690 [holotype of *Holorusia guzmani*]).

***Ischnotoma* (*Ischnotoma*) *peracuta* Alexander, 1971**: 1♂ (pinned), Chile, Llanquihue, Hornohuinco, XII/1968, Col. Peña, NMNH, det. C. P. Alexander, (USNMENT 01240739 [holotype]); 1♀ (pinned), Chile, Nahuelbuta, Pichinahuel, 1300m, I/15-20/1977, Col. Luis Peña, NMNH, det. C. P. Alexander (USNMENT 01518288); 1♂ (pinned), Chile, Nahuelbuta, Pichinahuel, 1300m, I/15-20/1977, Col. Luis Peña, NMNH, det. C. P. Alexander (USNMENT 01222506); 1♂ (slide), Chile, Llanquihue, Hornohuinco, #12630, XII/1968, Col. Peña, NMNH, det. C. P. Alexander, (USNMENT 01222685 [holotype]); 1♂ (slide), Chile, Nahuelbuta, Pichinahuel, 1300m, #12630, I/15-20/1977, Col. Luis Peña, NMNH, det. C. P. Alexander (USNMENT 01222703); 1♂ (slide), Chile, Malleco, Nahuelbuta, El Manzano, 800m, #12630, I/23-24/1977, Col. Luis Peña, NMNH, det. C. P. Alexander (USNMENT 01222702).

***Ischnotoma* (*Ischnotoma*) *porteri* (Alexander, 1929)**: 1♀ (pinned), Chile, Puerto Mont, 55925 USNM, XII/24/1926, Col. R. C. Shannon, NMNH, det. C. P. Alexander, (USNMENT 01222381 [paratype]); 1♀ (pinned), Chile, Chiloe, Castro, XII/15/1926, Col. R. C. Shannon, NMNH, det. C. P. Alexander [paratype]; 1♂ (pinned), Chile, Puerto Mont, XII/24/1926, Col. R. C. Shannon, NMNH, det. C. P. Alexander [paratype]; 1♀ (pinned), Chile, Valdivia, Panguipulli, XI/16/1923, Col. Alfredo Faz, NMNH, det. C. P. Alexander (USNMENT 01222382 [paratype]); 1♀ (pinned), Chile, Ñuble, Niblinto, El Roble, 35m E Coihueco, 650m, I/18/1918, Col. Peña, NMNH, det. C. P. Alexander (USNMENT 01222507); 1? (pinned), Chile, Cautin, Villarrica, 30Km NE, XII/16-31/1964, Col. L. E. Peña, NMNH, det. C. P. Alexander; 1♀ (pinned), Chile, Chiloé Island, Aucar, I/6-15/1952, Col. Peña, NMNH, det. C. P. Alexander; 1♂ (slide), Chile, Cautin, Villarrica, 30km NE, #3942, XII/16-31/1969, Col. Peña, NMNH, det. C. P. Alexander (USNMENT 01222926); 1♂ (pinned), Chile, La Araucanía Reg.; Malleco Province; Lonquimay, XII/1994, Col. Luis E. Peña, ANSP, det. J. Gouvêa (ANSP-ENT-133993).

***Ischnotoma* (*Ischnotoma*) *prionoceroides* Alexander, 1922**: 1♀ (pinned), Australia, Tasmania, Cradle Val., I/27/1923, Col. A. Tonnoir, NMNH, det. C. P. Alexander (USNMENT 01222508); 1♀ (slide), Australia, Tasmania, Summit, Mount Wellington, #1741, NMNH, det. C. P. Alexander (USNMENT 01222686 [holotype]); 1♂ (slide), Australia, Tasmania, Cradle Valley, #1741, I/22/1923, Col. A. Tonnoir, NMNH, det. C. P. Alexander (USNMENT 01222706 [allotype]); 1♀ (slide), Australia, Tasmania, Cradle Valley, 3500m, #1741, I/27/1923, Col. A. Tonnoir, NMNH, det. C. P. Alexander (USNMENT 01222707).

***Ischnotoma* (*Ischnotoma*) *problematica* (Alexander, 1945)**: 1♀ (pinned), Chile, Guape, I/25/1943, Col. Bullock, NMNH, det. C. P. Alexander (USNMENT 01240743 [holotype]); 1♀ (pinned), Chile, Guape, I/25/1943, NMNH, det. C. P. Alexander (USNMENT 01222835 [paratype]); 1♀ (slide), Chile, Guape, #7678, I/25/1943, Col. Bullock, NMNH, det. C. P. Alexander (USNMENT 01222786 [holotype]).

***Ischnotoma* (*Ischnotoma*) *rubriventris* (Macquart, 1846)**: 1? (pinned), Australia, Blue Mountains, Health Dept., I/7/1922, NMNH, det. C. P. Alexander; 1♂ (pinned), Australia, F. C. T., Canberra, X/1/1930, Col. A. L. Tonnoir, NMNH, det. C. P. Alexander (USNMENT 01222509); 1? (pinned) Australia, New South Wales, Dorrigo, III/14/1928, Col. W. Neron, NMNH, det. C. P. Alexander; 1? (pinned) Australia, Tasmania, King Island, Col. A. M. Lea, NMNH, det. C. P. Alexander; 1♂ (pinned), Australia, New South Wales, Barrington, S.U. Zoo Exp., I/1925, NMNH, det. C. P. Alexander; 1♂ (pinned), Australia, F. C. T., Blundell’s,, II/18/1931, Col. A. L. Tonnoir, NMNH, det. C. P. Alexander; 1? (pinned), Australia, Victoria, Woori Yallock, 1500m, IV/1927, Col. C. Barrett, NMNH, det. C. P. Alexander; 1♂ (slide) Australia, New South Wales, Dorrigo, #1690, III/14/1928, Col. W. Heron, NMNH, det. C. P. Alexander (USNMENT 01222924); 1♂ 1♀ (pinned), Australia, Victoria, near Sawmill in Healesville, 400m, III/25/1989, Col. P. Oosterbroek & C. Hartveld, NATURALIS.

***Ischnotoma* (*Ischnotoma*) *rubroabdominalis* Alexander, 1922**: 1♀ (pinned), Australia, Tasmania, Waratah, Col. H. J. Carter & A. M. Lea, NMNH, det. C. P. Alexander (USNMENT 01239035 [paratype]); 1♂ (pinned), Australia, Tasmania, Waratah, Col. H. J. Carter & A. M. Lea, NMNH, det. C. P. Alexander (USNMENT 01239036 [paratype]); 1? (pinned), Australia, Tasmania, Waratah, Col. H. J. Carter & A. M. Lea, NMNH, det. C. P. Alexander (USNMENT 01239036 [paratype]); 1♂ (pinned), Australia, Millthorpe, NMNH, det. C. P. Alexander; 1♂ (slide), Australia, Tasmania, Waratah, #1743, Col. Lea & Carter, NMNH, det. C. P. Alexander (USNMENT 01222922 [paratype]); 1♂ (slide), Australia, Tasmania, Cradle Valley, #1743, XI/1930, Col. G. Wendorfer, NMNH, det. C. P. Alexander (USNMENT 01222921).

***Ischnotoma* (*Ischnotoma*) *rufiventris* (Macquart, 1846)**: As *abnormalis*: 1♂ (slide), Australia, Tasmania, Eaglehawk Neck, #1744, XI/22/1922, Col. A. Tonnoir, NMNH, det. C. P. Alexander (USNMENT 01222996).

***Ischnotoma* (*Ischnotoma*) *rufistigmosa* (Macquart, 1838)**: 1♀ (pinned), Chile, Col. E. C. Reed, NMNH, det. C. P. Alexander (USNMENT 01222897); 1♀ (pinned), Chile, Col. E. C. Reed, NMNH, det. C. P. Alexander.

***Ischnotoma* (*Ischnotoma*) *schineriana* (Alexander, 1928)**: 1♂ (pinned), Chile, Marga Marga, II/20/1917, Col. A. Pirion, NMNH, det. C. P. Alexander [holotype]; 1♀ (pinned), Chile, Marga Marga, IX/17/1927, Col. Jaffuel & Pirion, NMNH, det. C. P. Alexander [paratype]; 1♀ (pinned), Chile, Marga Marga, 25/1928, Col. A. Pirion, NMNH, det. C. P. Alexander (USNMENT 01222899 [paratype]); 1♀ (pinned), Chile, Marga Marga, II/14/1928, Col. Pirion, NMNH, det. C. P. Alexander [paratype]; 1♀ (pinned), Chile, Santiago, Marga Marga, IX/13/1927, Col. Jaffuel & Pirion, NMNH, det. C. P. Alexander [paratype]; 1♂ (slide), Chile, Marga Marga, #3944, II/20/1917, Col. A. Pirion, NMNH, det. C. P. Alexander (USNMENT 01222799 [holotype]).

***Ischnotoma* (*Ischnotoma*) *scutellumnigrum* Alexander, 1924**: 1? (pinned), Australia, Mountain Kosciusko, XII/7/1922, Col. Goldfinch, NMNH, det. C. P. Alexander (USNMENT 01239037); 1♀ (slide), Australia, New South Wales, Mountain Kosciusko, #2699, XII/5/1921, Col. E. W. Ferguson, NMNH, det. C. P. Alexander (USNMENT 01222917 [allotype]); 1♂ (slide), Australia, New South Wales, Mountain Kosciusko, #2699, XII/7/1922, Col. Gilbert Goldfinch, NMNH, det. C. P. Alexander (USNMENT 01222916 [paratype]).

***Ischnotoma* (*Ischnotoma*) *shannoniana* (Alexander, 1929)**: 2♂ (pinned), Chile, Chiloe, Castro, XII/19/1926, Col. R. C. Shannon, NMNH, det. C. P. Alexander [paratype]; 3♂ (pinned), Chile, Chiloe, Ancud, XII/17-19/1926, Col. R. C. Shannon, NMNH, det. C. P. Alexander [paratype]; 2♀ (pinned), Chile, Chiloe Island, Aucar, I/6-15/1952, Col. Peña, NMNH, det. C. P. Alexander (USNMENT 01239030); 1♂ (pinned), Chile, Chiloe Island, Aucar, I/20/1952, Col. Peña, NMNH, det. C. P. Alexander; 1♂ (pinned), Chile, Llanquihue, Hornohuinco, X/1968, Col. Peña, NMNH, det. C. P. Alexander (USNMENT 01239031); 1♀ (slide), Chile, Llanquihue, Hornohuinco, #3945, X/1968, Col. L.E. Peña, NMNH, det. C. P. Alexander (USNMENT 01222911); 1♂ (slide), Chile, Chiloe, Ancud, #3945, I/20/1952, Col. Peña, NMNH, det. C. P. Alexander (USNMENT 01222913).

***Ischnotoma* (*Ischnotoma*) *silvai* (Alexander, 1929)**: 1♀ (pinned), Chile, Puerto Montt, XII/24/1926, Col. R. C. Shannon, NMNH, det. C. P. Alexander [paratype]; 1♂ (pinned), Chile, Valdivia, I/1924, NMNH, det. C. P. Alexander [paratype]; 1♀ (pinned), Chile, Panguipulli, I/17/1924, Col. Alfredo Faz, NMNH, det. C. P. Alexander [paratype]; 1? (pinned), Chile, Pillim Pilli, 600-800m, I/15/1954, Col. Luis Peña, NMNH, det. C. P. Alexander; 1? (pinned), Chile, Llanquihue, Hornohuinco, XII/1968, Col. L. E. Peña, NMNH, det. C. P. Alexander; 1? (pinned), Chile, Malleco, Nahuelbuta, 800m, I/23-24/1977, Col. L. Peña, NMNH, det. C. P. Alexander; 1♂ (slide), Chile, Malleco, Nahuelbuta, 800m, #3946, I/23-24/1977, Col. L. Peña, NMNH, det. C. P. Alexander (USNMENT 01222910).

***Ischnotoma* (*Ischnotoma*) *skuseana* Alexander, 1928**: 1♂ (pinned), Australia, Tasmania, Adventure Bay, XII/29/1922, Col. A. Tonnoir, [paratype]; 1♂ (pinned), Australia, Tasmania, Mount Wellington, I/1924, Col. G. H. Hardy, NMNH, det. C. P. Alexander (USNMENT 01239033 [paratype]); 1♂ (pinned), Australia, Tasmania, Mount Wellington, XI/25/1922, Col. A. Tonnoir, NMNH, det. C. P. Alexander (USNMENT 01239034 [paratype]); 1♂ (slide), Australia, Tasmania, Adventure Bay, #3366, XII/29/1922, Col. A. Tonnoir, (USNMENT 01222912 [paratype]); 1♂ (slide), Australia, Tasmania, Fern Tree, Mount Wellington, #3366, XI/11/1922, Col. A. Tonnoir, (USNMENT 01222909 [paratype]).

***Ischnotoma* (*Ischnotoma*) *terminata* Alexander, 1928**: 1♀ (pinned), Australia, Mount Wellington, XI/30/1922, Col. A. Tonnoir, NMNH, det. C. P. Alexander (USNMENT 01239026 [paratype]); 1♀ (slide), Australia, Mount Wellington, #3368, XI/30/1922, Col. A. Tonnoir, NMNH, det. C. P. Alexander (USNMENT 01222903 [paratype]).

***Ischnotoma* (*Ischnotoma*) *trunculata* (Alexander, 1962)**: 1♂ (pinned), Argentina, Neuquén, Lago Nacar, 650m, I/23/1954, Col. Schachovskoy, NMNH, det C. P. Alexander (USNMENT 01240752 [holotype]); 1♀ (pinned), Argentina, Neuquén, Lago Nacar, 650m, II/23/1954, Col. Schachovskoy, NMNH, det C. P. Alexander (USNMENT 01240756 [allotype]); 1♀ (pinned), Argentina, Lago Nonthué, II/4/1957, Col. Schachovskoy, NMNH, det C. P. Alexande; 1♀ (pinned) 1? (pinned), Argentina, Lago Nonthué, I/2/1951, Col. Schachovskoy, NMNH, det C. P. Alexander; 1? (pinned), Chile, Ñuble, El Roble, 35m E Coihueco, 650m, I/18-20/1968, Col. Peña, NMNH, det C. P. Alexander; 1♂ (slide), Argentina, Neuquén, Lago Nacar, 650m, #11089, I/23/1954, Col. Schachovskoy, NMNH, det C. P. Alexander (USNMENT 01222819 [holotype]); 1♂ (slide), Argentina, Lago Nonthué, #11089, I/2/1951, Col. Schachovskoy, NMNH, det C. P. Alexander (USNMENT 01222963).

***Ischnotoma* (*Neotipula*) *maya* (Alexander, 1912)**: 1♀ (pinned), Guatemala, Aguna, 1030ft, C. A., VIII/16/1902, Col. G. Eisen, NMNH, det. C. P. Alexander (USNMENT 01222401 [holotype]).

***Ischnotoma* (*Neotipula*) *paprzyckii* (Alexander, 1941)**: 1♀ (pinned), Peru, Satipo, V/2/1939, Col Paprzycki, NMNH, det. C. P. Alexander (USNMENT 01222403 [holotype]); 1 ♂ (pinned), Peru; Madre de Dios, Manu, Parque Manu, Pakitza; 11° 56’S, 71° 18’W, alt. 250m; #440, stream at Tr. 2-12, IX16/1989; Col. J. Gelhaus & M. Epstei, ANSP, (ANSP-ENT-133991); 1♂ (pinned), Peru; Cuzco, Quita Calzon (km164); 13°09’S, 71°22’W, alt. 1050m, #433, trib. Stream IX/2/1989, Col. J. Gelhaus,; ANSP, (ANSP-ENT-133990).

***Ischnotoma* (*Neotipula*) *pectinella* (Alexander, 1940)**: 1♂ (pinned), Panama, Potrerillos, Chiriqui, 3000’, V/20/1935, Col. J. W. MacSwain, NMNH, det. C. P. Alexander (USNMENT 01222402 [holotype]).

***Ischnotoma* (*Neotipula*) *penata* (Alexander, 1966)**: 1♀ (pinned), Ecuador, Quito, V/31/1963, NMNH, det. C. P. Alexander (USNMENT 01222404 [holotype]); 1♂ (pinned), Peru, Junin, Satipo, Jauja, 800-900m, VII/15/1940, Col P. Paprzycki, NMNH, det. C. P. Alexander (USNMENT 01222396); 1♀ (pinned), Peru,Junin, Satipo, Jauja, 800-900m, IV/13/1940, Col P. Paprzycki, NMNH, det. C. P. Alexander (USNMENT 01222395); 1♀ (slide), Ecuador, Quito, #11663, V/31/1963, NMNH, det. C. P. Alexander (USNMENT 01222688 [holotype]); 1♂ (slide), Peru, Junin, Satipo, Jauja, 800-900m, #11663, VII/15/1940, Col P. Paprzycki, NMNH, det. C. P. Alexander (USNMENT 01222928).

***Tipula* (*Tipula*) *oleracea* Linnaeus, 1758**: ???

***Zelandotipula* *diducta* Alexander, 1969**: 1♂ (pinned), Ecuador, San Francisco, Rio Pastaza, 1300m, X/1/1938, Col. Macintyr, NMNH, det. C. P. Alexander (USNMENT 01240542 [holotype]); 1♂ (pinned) 1♀ (pinned), Ecuador, San Francisco, Rio Pastaza, 1300m, X/1/1938, Col. Macintyr, NMNH, det. C. P. Alexander (USNMENT 01222888 [allotype and paratype]); 1♂ (slide), Ecuador, San Francisco, Rio Pastaza, 1300m, #11699, X/1/1938, Col. Macintyr, NMNH, det. C. P. Alexander (USNMENT 01222482 [holotype]).

***Zelandotipula* *flavicornis* (Alexander, 1914)**: 1? (pinned), Venezuela, Merida, La Mucuy, 2500m, VI/17/1938, Col. Berthier, NMNH, det. C. P. Alexander; 1♀ (pinned), Venezuela, Merida, Env. de Chachopo 2800m, VIII/7/1942, Col. R. Lichy, NMNH, det. C. P. Alexander; 1♀ (pinned), Venezuela, Maracay, Choroni, Rilo/6500, 980m, II/18/1939, Col. Ex. Anduae, NMNH, det. C. P. Alexander (USNMENT 01240570); 1♂ (slide), Venezuela, #911, Col. H. Fruhstorfer, NMNH, det. C. P. Alexander (USNMENT 01222473 [holotype]); 1♂ (slide), Venezuela, Merida, La Mucuy, 2500m, VI/17/1938, Col. Berthier, NMNH, det. C. P. Alexander (USNMENT 01222958); 1♂ (pinned), Peru, Dept. Cuzco, Qda. Morro Leguia, nr. Buenos Aires (km135) 2150m LT, VIII/28-29/1989, Col. J. Gelhaus, ANSP, det. J. Gouvêa (ANSP-ENT-133899); 1♀ (pinned), Peru, Dept. Cuzco, Qda. Morro Leguia, nr. Buenos Aires (km135) 2150m LT, VIII/28-29/1989, Col. J. Gelhaus, ANSP, det. J. Gouvêa (ANSP-ENT-133900); 1♀ (pinned), Ecuador, Pichincha Prov, stream, ca. 84kmSW Quito (km#84, Old St. Domingo-Chiriboga Road) elev 1400m. Bellardina-like larvae and pupa in wet leaf litter and shallow pools on bedrock outcrop seep along stream. Pupa seen ix/25/1990 Female emerged x/2/1990 PM see larval and pupal exuvia in ETOH (larva type#3), #489, IX/7/1990, Col. J. Gelhaus, ANSP, det. J. Gouvêa (ANSP-ENT-133894); 1♀ (pinned), Ecuador, Pichincha Prov, stream, ca. 84kmSW Quito (km#84, Old St. Domingo-Chiriboga Road) elev 1400m. Bellardina-like larvae and pupa in wet leaf litter and shallow pools on bedrock outcrop seep along stream. Pupa seen ix/25/1990 Female emerged x/2/1990 PM see larval and pupal exuvia in ETOH (larva type#3), #489, IX/7/1990, Col. J. Gelhaus, ANSP, det. J. Gouvêa (ANSP-ENT-133895); 1♂ (pinned), Peru, Dept. Cuzco, Qda. Morro Leguia nr. Buenos Aires (km135)2150m LT, #431, VIII/28-29/1989, Col. J. Gelhaus, ANSP, det. J. Gouvêa (ANSP-ENT-133986); 1♀ (pinned), Peru, Cuzco Dept., vic. Quebrada{=stream} Morro Leguia (km136,5); elev2220-2310m, VI/20/1989, Col. R. Bouchard, ANSP, det. J. Gouvêa (ANSP-ENT-133987).

***Zelandotipula* *furcifera* (Alexander, 1944)**: 1♂ (pinned), Costa Rica, Pedregoso, 2075’, I/1939, Col. D. L. Rounds, NMNH, det. C. P. Alexander (USNMENT 01222427 [holotype]); 1♂ (pinned), Costa Rica, Prov. San José, Moravia, Zurqui de Moravia, 1600m, Hand collected, Along stream ZADBI-1169. #107775 [Barcode] INB0004381267 INB OCRI Costa Rica, #1518, VIII/5/2013, Col. J. Gelhaus, ANSP, det. J. Gouvêa (ANSP-ENT-133934).

***Zelandotipula* *novarae* (Schiner, 1868)**: 1♂ (pinned) 1♀ (pinned), New Zealand, Auckland, St Heliers, X/27/1930, Col. A. T. Pyaoft, NMNH, det. C. P. Alexander; 1♀ (pinned), New Zealand, North Island, Kauari Forest, X/23/1978, Col. J. D. Hallaham, NMNH, det. C. P. Alexander; 1♂ (pinned), New Zealand, Westland, Ross, II/19/1923, T. R. Harris, NMNH, det. C. P. Alexander; 1♂ (pinned), New Zealand, Taihape, X/12/1921, T. R. Harris, NMNH, det. C. P. Alexander; 1♀ (pinned), New Zealand, Ohakune, 2060ft, X/5/1921, T. R. Harris, NMNH, det. C. P. Alexander; 1♂ (pinned) 2♀ (pinned), New Zealand, Auckland, Maraitai, Clevedon, III/19/1931, Col. Pritchard, NMNH, det. J. Gouvêa (USNMENT 01222883); 1♀ (pinned), New Zealand, Auckland, Clevedon, II/18-25/1931, Col. Pritchard, NMNH, det. J. Gouvêa (USNMENT 01222882); 2♂ (pinned), New Zealand, Auckland, Beachlands. II/1950, Col. J. W. Campbell, NMNH, det. J. Gouvêa; 1♂ (pinned), New Zealand, Col. J. W. Campbell, NMNH, det. J. Gouvêa; 1♂ (pinned), New Zealand, Kumara, III/9/1929, Col. J. W. C., NMNH, det. J. Gouvêa; 1♂ (pinned), New Zealand, Kumara, III/17/1929, Col. J. W. C., NMNH, det. J. Gouvêa; 1♀ (slide), New Zealand, Auckland, Mataivai, Clevedon, #1722, III/19/1931, Col. Pritchard, NMNH, det. C. P. Alexander (USNMENT 01222939); 1♂ (slide), New Zealand, Tisbury, #1722, X/18/1910, Col. A. Philpott, NMNH, det. C. P. Alexander (USNMENT 01222941); 1♂ (slide), New Zealand, Auckland, Mercer, Waikato, #1722, III/17/1932, Col. E. Pritchard, NMNH, det. C. P. Alexander (USNMENT 01222943); 1♂ (slide), New Zealand, Auckland, Mercer, Waikato, #1722, III/17/1932, Col. E. Pritchard, NMNH, det. C. P. Alexander (USNMENT 01222935); 1♂ (pinned), New Zealand, Ohakune, 2060ft, X/1921, Col. T. R. Harris, ANSP, det. C. P. Alexander; 1♂ (pinned), New Zealand, Ohakune, 2060ft, III/1/2002, Col. G. W. Cowper & C. M. Cowper, ANSP, det. C. P. Alexander (ANSP-ENT-133935); 1♀ (pinned), New Zealand, Pohara, III/1922, Col. Crouley, ANSP, det. C. P. Alexander; 1? (pinned), New Zealand, WN, Wainuiomata, Wright St. Bush, Lighttrap, X/22/1979, Col. J. R. Grehan, CMNH, det. J. Gouvêa.

***Zelandotipula* *otagana* (Alexander, 1922)**: 1♂ (pinned), New Zealand, Seaward moss, VIII/18/1906, NMNH, det. C. P. Alexander (USNMENT 01240738 [holotype]); 1♂ (pinned), New Zealand, Seaward moss, X/15/1905, NMNH, det. C. P. Alexander (USNMENT 01222531 [paratype]); 1♂ (pinned), New Zealand, Seaward moss, X/15/1905, NMNH, det. C. P. Alexander (USNMENT 01222532 [paratype]).

***Zelandotipula* *perstrangalia* (Alexander, 1962)**: 1♀ (pinned), Bolivia, Santa Cruz, Buenavista, NMNH, det. C. P. Alexander (USNMENT 01240740 [holotype]); 1♀ (slide), Bolivia, Santa Cruz, Buenavista, #11091, NMNH, det. C. P. Alexander (USNMENT 01222783 [holotype]); 1♂ (pinned), Peru, Madre de Dios, Parque Maru Paktiza, 250m. seepage area, Plot 1, IX/16/1989, Col. J. Gelhaus, ANSP, det. Jon K. Gelhaus (ANSP-ENT-133936); 1♀ (pinned), Peru, Madre de Dios, Parque Maru Paktiza, 250m. seepage area, Plot 1, IX/16/1989, Col. J. Gelhaus, ANSP, det. Jon K. Gelhaus (ANSP-ENT-133937); 1♂ (pinned), Peru, Madre de Dios, Parque Maru Paktiza, 250m. seepage area at Rd-1, #441, IX/17/1989, Col. J. Gelhaus, ANSP, det. Jon K. Gelhaus (ANSP-ENT-133909); 1♀ (pinned), Ecuador, Zamora-Chinchipe. 8kmNW Zamora, mouth Rio Sabanilla 1420m, XI/1/1987, Col. C. Young, R. Davidson & J. Rawlins, CMNH, det. J. Gouvêa (CMNH-9,232).

**Ingroup:**

***Icriomastax* *antinympha* (Alexander, 1942)**: 1♀ (pinned), Brazil, São Paulo, Juquiá, 400m, #60124, XI/1940, Col. L. Travassos, MZSP, det. C. P. Alexander (MZ 001745 [holotype]); 1♀ (slide), Brazil, São Paulo, Juquiá, 400m, #7198, XI/1940, Col. L. Travassos, NMNH, det. C. P. Alexander (USNMENT 01222590 [holotype]); 1♂ (ethanol), Brazil, Rio de Janeiro, VIII/2011, Col. J. C. Bernardes, MNRJ, det. J. Gouvêa (MNRJ 30,034) (lost in the 2018 fire in National Museum); 1♂ (ethanol), Brazil, Nova Friburgo, Rio Cascatinha, Alto Cascatinha, 28/IX/1991, MNRJ, det. J. Gouvêa (MNRJ 30,284) (lost in the 2018 fire in National Museum); 11♂ (ethanol), Brazil, Rio de Janeiro, PARNA Tijuca, Represa dos Ciganos, 1968, Col. C. Alvarenga, MNRJ, det. J. Gouvêa (MNRJ 30,568) (lost in the 2018 fire in National Museum); 1♂ (ethanol), Brazil, Rio de Janeiro, PARNA Tijuca, Represa dos Ciganos, 1968, Col. C. Alvarenga, MNRJ, det. J. Gouvêa (MNRJ 30,569) (lost in the 2018 fire in National Museum); 1♀ (ethanol), Brazil, Rio de Janeiro, PARNA Tijuca, Represa dos Ciganos, 1968, Col. C. Alvarenga, MNRJ, det. J. Gouvêa (MNRJ 30,570) (lost in the 2018 fire in National Museum); 10♂ (ethanol), Brazil, Rio de Janeiro, PARNA Tijuca, Represa dos Ciganos, 1968, Col. C. Alvarenga, MNRJ, det. J. Gouvêa (MNRJ 30,571) (lost in the 2018 fire in National Museum); 12♂ (ethanol), Brazil, Rio de Janeiro, PARNA Tijuca, Represa dos Ciganos, II/1967, Col. Hugo Souza Lopes, MNRJ, det. J. Gouvêa (MNRJ 30,572) (lost in the 2018 fire in National Museum); 8♂ (ethanol), Brazil, Rio de Janeiro, PARNA Tijuca, Represa dos Ciganos, II/1967, Col. Hugo Souza Lopes, MNRJ, det. J. Gouvêa (MNRJ 30,573) (lost in the 2018 fire in National Museum); 10♂ (ethanol), Brazil, Rio de Janeiro, PARNA Tijuca, Represa dos Ciganos, 1968, Col. C. Alvarenga, MNRJ, det. J. Gouvêa (MNRJ 30,574) (lost in the 2018 fire in National Museum); 3♀ (ethanol), Brazil, Rio de Janeiro, PARNA Tijuca, Represa dos Ciganos, 1968, Col. C. Alvarenga, MNRJ, det. J. Gouvêa (MNRJ 30,575) (lost in the 2018 fire in National Museum); 7♂ (ethanol), Brazil, Rio de Janeiro, PARNA Tijuca, Represa dos Ciganos, II/1967, Col. Hugo Souza Lopes, MNRJ, det. J. Gouvêa (MNRJ 30,576) (lost in the 2018 fire in National Museum);

10♂ (ethanol), Brazil, Rio de Janeiro, PARNA Tijuca, Represa dos Ciganos, 1968, Col. C. Alvarenga, MNRJ, det. J. Gouvêa (MNRJ 30,578) (lost in the 2018 fire in National Museum); 11♂ (ethanol), Brazil, Rio de Janeiro, PARNA Tijuca, Represa dos Ciganos, 1968, Col. C. Alvarenga, MNRJ, det. J. Gouvêa (MNRJ 30,597) (lost in the 2018 fire in National Museum); 2♀ (ethanol), Brazil, Rio de Janeiro, PARNA Tijuca, Represa dos Ciganos, 1968, Col. C. Alvarenga, MNRJ, det. J. Gouvêa (MNRJ 30,598) (lost in the 2018 fire in National Museum); 10♂ (ethanol), Brazil, Rio de Janeiro, PARNA Tijuca, Represa dos Ciganos, 1968, Col. C. Alvarenga, MNRJ, det. J. Gouvêa (MNRJ 30,599) (lost in the 2018 fire in National Museum); 10♂ (ethanol), Brazil, Rio de Janeiro, PARNA Tijuca, Represa dos Ciganos, 1968, Col. C. Alvarenga, MNRJ, det. J. Gouvêa (MNRJ 30,600) (lost in the 2018 fire in National Museum); 10♂ (ethanol), Brazil, Rio de Janeiro, PARNA Tijuca, Represa dos Ciganos, 1968, Col. C. Alvarenga, MNRJ, det. J. Gouvêa (MNRJ 30,601) (lost in the 2018 fire in National Museum); 10♂ (ethanol), Brazil, Rio de Janeiro, PARNA Tijuca, Represa dos Ciganos, 1968, Col. C. Alvarenga, MNRJ, det. J. Gouvêa (MNRJ 30,603) (lost in the 2018 fire in National Museum); 10♂ (ethanol), Brazil, Rio de Janeiro, PARNA Tijuca, Represa dos Ciganos, 1968, Col. C. Alvarenga, MNRJ, det. J. Gouvêa (MNRJ 30,604) (lost in the 2018 fire in National Museum); 10♂ (ethanol), Brazil, Rio de Janeiro, PARNA Tijuca, Represa dos Ciganos, 1968, Col. C. Alvarenga, MNRJ, det. J. Gouvêa (MNRJ 30,605) (lost in the 2018 fire in National Museum); 10♂ (ethanol), Brazil, Rio de Janeiro, PARNA Tijuca, Represa dos Ciganos, 1968, Col. C. Alvarenga, MNRJ, det. J. Gouvêa (MNRJ 30,606) (lost in the 2018 fire in National Museum); 10♂ (ethanol), Brazil, Rio de Janeiro, PARNA Tijuca, Represa dos Ciganos, 1968, Col. C. Alvarenga, MNRJ, det. J. Gouvêa (MNRJ 30,609) (lost in the 2018 fire in National Museum); 10♂ (ethanol), Brazil, Rio de Janeiro, PARNA Tijuca, Represa dos Ciganos, 1968, Col. C. Alvarenga, MNRJ, det. J. Gouvêa (MNRJ 30,610) (lost in the 2018 fire in National Museum); 4♀ (ethanol), Brazil, Rio de Janeiro, PARNA Tijuca, Represa dos Ciganos, II/1967, Col. Hugo Souza Lopes, MNRJ, det. J. Gouvêa (MNRJ 30,615) (lost in the 2018 fire in National Museum); 2♂ (ethanol), Brazil, Rio de Janeiro, Itatiaia, Parque Nacional do Itatiaia, Casa do Pesquisador (Leo’s House), 22°27’16,29’’S 44°36’29,23’’W, 806m, 2/XI/2015, Col. A. P. Pinto, MNRJ, det. J. Gouvêa (MNRJ 30,630) (lost in the 2018 fire in National Museum); 1♂2♀ (ethanol), Brazil, Rio de Janeiro, Itatiaia, Parque Nacional do Itatiaia, Casa do Pesquisador (Leo’s House), 22°27’16,29’’S 44°36’29,23’’W, 806m, 3/XI/2015, Col. L. H. Gil-Azevedo, MNRJ, det. J. Gouvêa (MNRJ 30,632) (lost in the 2018 fire in National Museum); 4♂ (ethanol), Brazil, Rio de Janeiro, Itatiaia, Parque Nacional do Itatiaia, Casa do Pesquisador (Leo’s House), 22°27’16,29’’S 44°36’29,23’’W, 806m, 5/XI/2015, Col. L. H. Gil-Azevedo, MNRJ, det. J. Gouvêa (MNRJ 30,648) (lost in the 2018 fire in National Museum); 1♀ (ethanol), Brazil, Rio de Janeiro, Teresópolis, PNSO, Rio Beija Flor (PAC17), 22°26’50,9’’S 43°00’19,4’’W, 1187m, 11-15/XI/2011, Col. A. P. M. Santos, B. Camisão & J. L. Nessimian, MNRJ, det. J. Gouvêa (MNRJ 30,721) (lost in the 2018 fire in National Museum); 1♂ (ethanol), Brazil, Rio de Janeiro, Teresópolis, PARNASO, Casa do Pesquisador, 13/XII/2017, Col. C. C. D. Correa, MNRJ, det. J. Gouvêa (MNRJ 30,830) (lost in the 2018 fire in National Museum); 1♂ (ethanol), Brazil, Rio de Janeiro, Teresópolis, Trilha, 13/XII/2017, Col. L. H. Gil-Azevedo, MNRJ, det. J. Gouvêa (MNRJ 30,831) (lost in the 2018 fire in National Museum); 1♂ (ethanol), Brazil, Rio de Janeiro, Itatiaia, Parque Nacional do Itatiaia, Complexo do Maromba, Travessia Ruy Braga, PNI-MB2, 22°26’07,50’’S 44°37’33,20’’W, 1234m, 2/VII-3/VIII/2015, Col. A.A.P., J.B.M., ACA, MNRJ, det. J. Gouvêa (MNRJ 30,859) (lost in the 2018 fire in National Museum); 2♀ (ethanol), Brazil, Minas Gerais, Itamonte, Parque Nacional do Itatiaia, Setor Brejo da Lapa, PNI-M1A, 22°21’32,40’’S 44°44’14,04’’W, 2142m, 5/IV-5/V/2016, Col. M.L.M., J.P.B, A.C.A., MNRJ, det. J. Gouvêa (MNRJ 30,872) (lost in the 2018 fire in National Museum); 2♀ (ethanol), Brazil, Minas Gerais, Itamonte, Parque Nacional do Itatiaia, Complexo do Maromba, PNI-M2A, 22°25’36,10’’S 44°37’05,80’’W, 1153m, 4/XII/2015-6/I/2016, Col. M.L.M., A.P.P., A.C.A., MNRJ, det. J. Gouvêa (MNRJ 30,874) (lost in the 2018 fire in National Museum); 1♀ (ethanol), Brazil, Minas Gerais, Itamonte, Parque Nacional do Itatiaia, Complexo do Maromba, PNI-M2A, 22°25’36,10’’S 44°37’05,80’’W, 1153m, 3/VIII/-1/IX/2015, Col. A.L.C., A.P.P., O.S.M., MNRJ, det. J. Gouvêa (MNRJ 30,875) (lost in the 2018 fire in National Museum); 5♂ (ethanol), Brazil, Rio de Janeiro, Petrópolis, Araras, Rebio Araras, Trilha do Caneco, Afluente do Rio Araras, Ara14, Malaise, 9/I-28/I/2020, Col. L.L.D., J.L.N., C.N.M.B., N.H.P., MNRJ, det. J. Gouvêa (MNRJ 31,494).

***Icriomastax* *calliope* (Alexander, 1945)**: 1♂ (pinned) 1♀ (pinned), Brazil, São Paulo, Guarujá, 50m, I/21/1942, Col. Carrera, NMNH, det. C. P. Alexander (USNMENT 01240521 [holotype and allotype]); 1♂ (slide), Brazil, São Paulo, Guarujá, 50m, #13091, I/21/1942, Col. Carrera, NMNH, det. C. P. Alexander (USNMENT 01222593 [holotype]); 1♀ (slide), Brazil, São Paulo, Guarujá, 50m, #13091, I/21/1942, Col. Carrera, NMNH, det. C. P. Alexander (USNMENT 01222594 [allotype]); 1♀ (ethanol), Brazil, Rio de Janeiro, Tijuca, Floresta da Tijuca, XII/2012, Col. J. C. Bernardes, MNRJ, det. J. Gouvêa (MNRJ 30,081) (lost in the 2018 fire in National Museum); 2♂1♀ (ethanol), Brazil, Rio de Janeiro, Itatiaia, PNI, Casa do Pesquisador (Leo House), 22°27’16,29’’S 44°36’29,23’’W, 3/XI/2015, Col. L. H. Gil-Azevedo, MNRJ, det. J. Gouvêa (MNRJ 30,620) (lost in the 2018 fire in National Museum); 1♀ (ethanol), Brazil, Paraná, Curitiba, 25/VII/1954, Col. N. Santos, MNRJ, det. J. Gouvêa (MNRJ 30,757) (lost in the 2018 fire in National Museum); 1♀ (ethanol), Brazil, Paraná, Curitiba, 25/VII/1954, Col. N. Santos, MNRJ, det. J. Gouvêa (MNRJ 30,758) (lost in the 2018 fire in National Museum); 1♀ (ethanol), Brazil, Minas Gerais, Conceição da Aparecida, Fazenda São José, II/1960, Col. J. C. M. Carvalho, MNRJ, det. J. Gouvêa (MNRJ 30,782) (lost in the 2018 fire in National Museum); 1♀ (ethanol), Brazil, Minas Gerais, Itamonte, Parque Nacional do Itatiaia, Setor Brejo da Lapa, PNI-M1A, 22°21’32,40’’S 44°44’14,04’’W, 2142m, 5/IV-5/V/2016, Col. M.L.M., J.P.B, A.C.A., MNRJ, det. J. Gouvêa (MNRJ 30,870) (lost in the 2018 fire in National Museum); 1♀ (ethanol), Brazil, Rio de Janeiro, Itatiaia, Parque Nacional do Itatiaia, Setor Lago Azul, Rio Campo Belo, PNI-M3A, 22°26’49,20’’S 44°36’45,00’’W, 854m, 4/IV-6/V/2016, Col. M.L.M., J.P.B, A.C.A., MNRJ, det. J. Gouvêa (MNRJ 30,878) (lost in the 2018 fire in National Museum); 1♀ (ethanol), Brazil, Rio de Janeiro, Parque Nacional da Tijuca, Rio Taquaruçu (Abaixo da Cachoeira), 22°57’36,20’’S 43°17’36,2’’W, 509m, 7/IX/2016, Col. J.L. Nessimian, L.L. Dumas, B.M. Silva, F. Q. Machado, MNRJ, det. J. Gouvêa (MNRJ 31,002); 1♀ (ethanol), Brazil, Rio de Janeiro, Nova Iguaçu, Rebio Tinguá, Sede, 14-16/II/2020, Col. C. C. D. Correa, MNRJ, det. J. Gouvêa (MNRJ 31,481).

***Icriomastax catia*** sp. nov.: see description.

***Icriomastax craigi*** sp. nov.: see description.

***Icriomastax coscaroni*** sp. nov.: see description.

***Icriomastax* *euterpe* (Alexander, 1945)**: 2♂ (pinned), Brazil, Rio de Janeiro, I-III/1941, Col. Tulloch, NMNH, det. C. P. Alexander (USNMENT 01240520 [holotype and paratype]); 1? (pinned), Brazil, Rio de Janeiro, Distrito Federal, X/9/1931, Col. D. F., NMNH, det. C. P. Alexander [paratype]; 2♂ (pinned) 1? (pinned), Brazil, Rio de Janeiro, NMNH, det. C. P. Alexander [paratype]; 1♂ (slide), Brazil, Rio de Janeiro, #7875, I-III/1941, Col. Tulloch, NMNH, det. C. P. Alexander (USNMENT 01222476 [holotype]); 3♂ (ethanol), Brazil, Rio de Janeiro, Itatiaia, PNI, Casa do Pesquisador (Leo House), 22°27’14,9’’S 44°36’27,8’’W, 821m, 2/XI/2015, Col. L. H. Gil-Azevedo, MNRJ, det. J. Gouvêa (MNRJ 30,519) (lost in the 2018 fire in National Museum); 1♀ (ethanol), Brazil, Rio de Janeiro, Itatiaia, PNI, Casa do Pesquisador (Leo House), 22°27’14,9’’S 44°36’27,8’’W, 821m, 2/XI/2015, Col. L. H. Gil-Azevedo, MNRJ, det. J. Gouvêa (MNRJ 30,520) (lost in the 2018 fire in National Museum); 1♂ (ethanol), Brazil, Rio de Janeiro, Itatiaia, PNI, Casa do Pesquisador (Leo House), 22°27’14,9’’S 44°36’27,8’’W, 821m, 2/XI/2015, Col. L. H. Gil-Azevedo, MNRJ, det. J. Gouvêa (MNRJ 30,616) (lost in the 2018 fire in National Museum); 1♀ (ethanol), Brazil, Rio de Janeiro, Itatiaia, PNI, Casa do Pesquisador (Leo House), 22°27’16,29’’S 44°36’29,23’’W, 806m, 5-8/I/2016, Col. M. Monné, MNRJ, det. J. Gouvêa (MNRJ 30,622) (lost in the 2018 fire in National Museum); 1♂1♀ (ethanol), Brazil, Rio de Janeiro, Itatiaia, PNI, Casa do Pesquisador (Leo House), 22°27’16,29’’S 44°36’29,23’’W, 806m, 5/IV/2016, Col. L. H. Gil-Azevedo, MNRJ, det. J. Gouvêa (MNRJ 30,623) (lost in the 2018 fire in National Museum); 6♂ (ethanol), Brazil, Rio de Janeiro, Itatiaia, PNI, Casa do Pesquisador (Leo House), 22°27’16,29’’S 44°36’29,23’’W, 806m, 5/XI/2015, Col. L. H. Gil-Azevedo, MNRJ, det. J. Gouvêa (MNRJ 30,623) (lost in the 2018 fire in National Museum); 1♀ (ethanol), Brazil, Paraná, Curitiba, 25/VII/1954, Col. N. Santos, MNRJ, det. J. Gouvêa (MNRJ 30,756) (lost in the 2018 fire in National Museum);1♀ (pinned), Brazil, Rio de Janeiro, Itatiaia, PNI, Retiro, VI/1902, Col. Carlos Moreira, MNRJ, det. J. Gouvêa (MNRJ 30,763) (lost in the 2018 fire in National Museum); 1♀ (ethanol), Brazil, Minas Gerais, Conceição de Aparecida, Fazenda São José, II/1960, Col. J. C. M. Carvalho, MNRJ, det. J. Gouvêa (MNRJ 30,764) (lost in the 2018 fire in National Museum).

***Icriomastax* *helios* (Alexander, 1949)**: 1♂ (pinned), Brazil, Paraná, Curitiba, VI/1943, Col. Hatschbach, NMNH, det. C. P. Alexander (USNMENT 01240526 [holotype]); 1♀ (pinned), Brazil, São Paulo, Ypiranga, 800m, on banana, V/27/1941, Col. D’amica, MZSP, det. C. P. Alexander (MZ 001746 [allotype]); 1♀ (pinned), Brazil, São Paulo, Ypiranga, 800m, X/11/1941, Col. D’amico, NMNH, det. C. P. Alexander (USNMENT 01240584 [paratype]); 1♀ (pinned), Brazil, São Paulo, Ypiranga, XI/7/1944, Col. F. Lane, NMNH, det. C. P. Alexander (USNMENT 01240575 [paratype]); 1♂ (pinned), Brazil, Santa Catarina, Neu Bremen, V/18/1936, Col. Hoffmann, NMNH, det. C. P. Alexander (USNMENT 01240526 [paratype]); 1♀ (pinned), Brazil, Santa Catarina, Neu Bremen, XI/1/1936, Col. Hoffmann, NMNH, det. C. P. Alexander (USNMENT 01222576 [paratype]); 1♀ (pinned), Brazil, São Paulo, Ribeirão Pires, Barreira das Amelias, XI/1941, Col. D’amico, MZSP, det. C. P. Alexander [paratype]; 1♂ (pinned), Brazil, São Paulo, C. Jordão, XII/1945, Col. F. Lane, NMNH, det. C. P. Alexander; 1♂ (pinned), Brazil, São Paulo, Barueri, at light, X/28/1955, Col. Karol Lenko, NMNH, det. J. Gouvêa; 1♂ (pinned), Brazil, São Paulo, Barueri, at light, XI/21/1955, Col. Karol Lenko, NMNH, det. J. Gouvêa; 1♂ (pinned), Brazil, São Paulo, Barueri, at light, XI/25/1955, Col. Karol Lenko, NMNH, det. J. Gouvêa; 1♂ (pinned), Brazil, São Paulo, Barueri, at light, X/15/1955, Col. Karol Lenko, NMNH, det. J. Gouvêa; 1♂ (pinned), Brazil, São Paulo, Barueri, at light, VIII/7/1955, Col. Karol Lenko, NMNH, det. J. Gouvêa; 1♂ (pinned), Brazil, São Paulo, Barueri, at light, II/22/1955, Col. Karol Lenko, NMNH, det. J. Gouvêa; 1♂ (pinned), Brazil, São Paulo, Barueri, at light, IV/21/1955, Col. Karol Lenko, NMNH, det. J. Gouvêa; 1♂ (pinned), Brazil, São Paulo, Barueri, at light, X/29/1955, Col. Karol Lenko, NMNH, det. J. Gouvêa; 1♂ (pinned), Brazil, São Paulo, Barueri, X/25/1955, Col. Karol Lenko, NMNH, det. J. Gouvêa; 1♂ (pinned), Brazil, São Paulo, Barueri, XI/8/1955, Col. Karol Lenko, NMNH, det. J. Gouvêa; 2♂ (pinned), Brazil, São Paulo, Barueri, XI/16/1955, Col. Karol Lenko, NMNH, det. J. Gouvêa; 1♂ (pinned), Brazil, São Paulo, Barueri, X/26/1955, Col. Karol Lenko, NMNH, det. J. Gouvêa; 1♂ (pinned), Brazil, São Paulo, Campos do Jordão, Lefevre, IX/1953, Col. L. Travassos Filho & P. de Pereira, NMNH, det. J. Gouvêa; 1♂ (slide), Brazil, Paraná, Curitiba, #13092, VI/1943, Col. Hatschbach, NMNH, det. C. P. Alexander (USNMENT 01222591 [holotype]); ]); 1♀ (slide), Brazil, São Paulo, Ypiranga, #13092, XI/7/1944, Col. F. Lane, NMNH, det. C. P. Alexander (USNMENT 01222580 [paratype]); 1♂ (slide), Brazil, Santa Catarina, Neu Bremen, #13092, V/18/1936, Col. Hoffmann, NMNH, det. C. P. Alexander (USNMENT 01222582 [paratype]); 1♂ (slide), Brazil, São Paulo, C. Jordão, #13092, XII/1945, Col. J. Lane, NMNH, det. C. P. Alexander (USNMENT 01222711); 1♀ (ethanol), Brazil, Minas Gerais, Itamonte, Ponto da Fazenda, Rio Aiuruoca, 7/IV/2005, Col. J. L. Nessimian, N. Pereira Jr. , MNRJ, det. J. Gouvêa (MNRJ 30,288) (lost in the 2018 fire in National Museum);

1♀ (ethanol), Brazil, Paraná, Curitiba, 25/VII/1954, Col. N. Santos, MNRJ, det. J. Gouvêa (MNRJ 30,759) (lost in the 2018 fire in National Museum).

***Icriomastax lopesae*** sp. nov.: see description.

***Icriomastax monnei*** sp. nov.: see description.

***Icriomastax* *nebulipennata* (Alexander, 1980)**: 1♂ (slide), Argentina, Catamarca, Aconquija, 1700m, #13503, X/2/1968, Col. Luis Peña, NMNH, det. C. P. Alexander (USNMENT 01222701 [holotype]).

***Icriomastax* *nitra* (Alexander, 1945)**: 1♂ (pinned), Brazil, Rio, Teresópolis, 920m, VIII/1942, Col. Gomes, NMNH, det. C. P. Alexander (USNMENT 01240523 [holotype]); 1♂ (pinned), Brazil, Rio, Teresópolis, 920m, VIII/1942, Col. Gomes, NMNH, det. C. P. Alexander [allotype]; 1♂ (pinned), Brazil, Rio, Teresópolis, Organ Mountains,1000m, VII/1942, Col. Gomes, NMNH, det. C. P. Alexander (USNMENT 01222511 [paratype]); 1? (pinned), Brazil, Rio, Teresópolis, 1000m, Col. Gomes, VIII/1942, NMNH, det. C. P. Alexander; 1♂ (slide), Brazil, Rio de Janeiro, Teresópolis, Organ Mountains, 920m, #7876, VIII/1942, Col. Lério Gomes, NMNH, det. C. P. Alexander (USNMENT 01222585 [paratype]); 1♂ (slide), Brazil, Rio de Janeiro, Teresópolis, 1000m, #7876, VIII/1942, Col. L. Gomes, NMNH, det. C. P. Alexander (USNMENT 01222586); 1♀ (slide), Brazil, São Paulo, Butantan, Horto Oswaldo Cruz, #7876, IV/2/1974, Col. Travassos, NMNH, det. C. P. Alexander (USNMENT 01222712); 1♀ (ethanol), Brazil, Minas Gerais, Itamonte, Ponto da Fazenda, Rio Aiuruoca, 7/IV/2005, Col. J. L. Nessimian, N. Pereira Jr. , MNRJ, det. J. Gouvêa (MNRJ 30,287) (lost in the 2018 fire in National Museum); 1♂ (ethanol), Brazil, Rio de Janeiro, Itatiaia, PNI, Casa do Pesquisador (Leo’s House), 22°27’16,29’’S 44°36’29,23’’W, 806m, 2/XI/2015, Col. L.H.Gil-Azevedo, MNRJ, det. J. Gouvêa (MNRJ 30,617) (lost in the 2018 fire in National Museum); 1♀ (ethanol), Brazil, Paraná, Curitiba, 25/VII/1954, Col. N. Santos, MNRJ, det. J. Gouvêa (MNRJ 30,760) (lost in the 2018 fire in National Museum); 1♂ (ethanol), Brazil, Minas Gerais, Santana do Riacho, Serra do Cipó, 17/I/1951, Col. Machado & Santos, MNRJ, det. J. Gouvêa (MNRJ 30,815) (lost in the 2018 fire in National Museum).

***Icriomastax* *nudicornis* (Macquart, 1838)**: 1♀ (pinned), Argentina, Jujuy, Tilcara, I/12/1920, Col. Weiser V., NMNH, det. C. P. Alexander (USNMENT 01240528 [holotype]); 1♂ (pinned), Argentina, Cordoba, Alta Gracia, La Granja, IV/1-8/1920, Col. C. Bruch, NMNH, det. C. P. Alexander [paratype]; 3♂ (pinned) 2? (pinned), Argentina, Cordoba, Alta Gracia, IV/1-8/1920, Col. C. Bruch, NMNH, det. C. P. Alexander [paratype]; 1♂ (pinned), Argentina, Cordoba, Arroyo de Tanti, III/26/1966, Col. A. Hemmingsen, NMNH, det. C. P. Alexander; 1? (pinned), Argentina, Catamarca, El Suncho, II/20/1931, Col. N. Kosnesso, NMNH, det. C. P. Alexander; 1♀ (pinned), Argentina, Cordoba, Unquillo, Cabana, #T2975, XI/9/1926, Col. C. Bruch, NMNH, det. C. P. Alexander; 1♀ (pinned), Argentina, Cordoba, Unquillo, Cabana, #T2976, I/23/1926, Col. C. Bruch, NMNH, det. C. P. Alexander; 1♀ (pinned), Argentina, Cordoba, Unquillo, Cabana, #T2978, I/4/1926, Col. C. Bruch, NMNH, det. C. P. Alexander; 1♀ (pinned), Argentina, Cordoba, Unquillo, Cabana, #T2977, I/2/1926, Col. C. Bruch, NMNH, det. C. P. Alexander; 1? (pinned), Argentina, Cordoba, So Amer Paras Lab, No 853-6, IX/7/1943, Col. Silveira, NMNH, det. C. P. Alexander; 1♀ (pinned), Argentina, Cordoba, So Amer Paras Lab, No 853-6, IX/7/1943, Col. Silveira, NMNH, det. C. P. Alexander; 1♂ (pinned), Argentina, Catamarca, Aconquija, 1700m, X/2/1968, Col. L.E.Peña, NMNH, det. C. P. Alexander (USNMENT 01222549); 1♂ (pinned) 1? (pinned), Argentina, Catamarca, Aconquija, 1700m, X/2/1968, Col. L.E.Peña, NMNH, det. C. P. Alexander; 1? (pinned), Argentina, Tucuman, Tafi, II/17/1953, NMNH, det. C. P. Alexander; 1♀ (SLIDE), Argentina, Jujuy, Tilcara, #1462, I/12/1920, Col. Weiser V., NMNH, det. C. P. Alexander (USNMENT 01222587 [holotype]); 1♂ (slide), Argentina, Cordoba, Alta Gracia, La Granja, #1462, IV/1-8/1920, Col. C. Bruch, NMNH, det. C. P. Alexander (USNMENT 01222708 [paratype]); 1♂ (slide), Argentina, Cordoba, Alta Gracia, La Granja, #1462, IV/1-8/1920, Col. C. Bruch, NMNH, det. C. P. Alexander (USNMENT 01222588 [paratype]); 1♂ (slide), Argentina, Catamarca, El Suncho, #1462, II/20/1931, NMNH, det. C. P. Alexander (USNMENT 01222714); 1♂ (slide), Argentina, Catamarca, Aconquija, 1700m, #1462, X/2/1968, Col. L.E.Peña, NMNH, det. C. P. Alexander (USNMENT 01222589); 1♂ (slide), Argentina, Catamarca, Aconquija, 1700m, #1462, X/2/1968, Col. L.E.Peña, NMNH, det. C. P. Alexander (USNMENT 01222713); 1♂ (slide), Argentina, Cordoba, Arroyo de Tanti, #1462, III/26/1966, Col. A. Hemmingsen, NMNH, det. C. P. Alexander (USNMENT 01222705); 1♂ (slide), Argentina, Cordoba, Alta Gracia, La Granja, #1462, IV/1-8/1920, Col. Bruch, NMNH, det. C. P. Alexander (USNMENT 01222709); 1? (pinned), Argentina,Catamarca, N. Aconquija, 1700m, X/2/1968, Col. L. E. Peña, MZLU, det. C. P. Alexander (MZLU-DIPT 00044636).

***Icriomastax* *ocellata* (Enderlein, 1912)**: 1? (pinned), Brazil, Minas Geraes, A. Caparaó, 2650m, XII/1941, Col. L. Gomes, NMNH, det. C. P. Alexander; 1? (pinned), Brazil, Campo Belo, 1100m, VII/5/1929, Col. Schade, NMNH, det. C. P. Alexander; 1? (pinned), Brazil, Nova Teutonia, Brasilien, XI/1938, Col. Fritz Plaumann, NMNH, det. C. P. Alexander (USNMENT 01240525); 1♂ (slide), Brazil, Santa Catarina, Nova Teutonia, Brasilien, #3380, XI/29/1938, Col. Plaumann, NMNH, det. C. P. Alexander (USNMENT 01222718); 1♂ (ethanol), Brazil, Rio de Janeiro, Itatiaia, PNI, BR485 entre posto Marcão e Trilha para as Agulhas Negras, 17/I/2016, Col. A.P.P., M.L.M, F.L.N., MNRJ, det. J. Gouvêa (MNRJ 30,617) (lost in the 2018 fire in National Museum); 1♀ (pinned), Brazil, Rio de Janeiro, Itatiaia, PNI, Retiro, XII/1903, Col. Carlos Moreira, MNRJ, det. J. Gouvêa (MNRJ 30,762) (lost in the 2018 fire in National Museum); 1♂ (ethanol), Brazil, Minas Gerais, Itamonte, Parque Nacional do Itatiaia, Complexo do Maromba, PNI-M2A, 22°25’36,10’’S 44°37’05,80’’W, 1153m, 3/VIII/-1/IX/2015, Col. A.L.C., A.P.P., O.S.M., MNRJ, det. J. Gouvêa (MNRJ 30,840) (lost in the 2018 fire in National Museum); 1♂ (ethanol), Brazil, Minas Gerais, Itamonte, Parque Nacional do Itatiaia, Setor Brejo da Lapa, PNI-M1A, 22°21’32,40’’S 44°44’14,04’’W, 2142m, 5/IV-5/V/2016, Col. M.L.M., J.P.B, A.C.A., MNRJ, det. J. Gouvêa (MNRJ 30,873) (lost in the 2018 fire in National Museum); 1♂1♀ (ethanol), Brazil, Rio de Janeiro, Teresópolis, PARNASO, Cachoeira de Papel, 26/VII-4/IX/2021, Col. S. Vaz, A. Freitas, M. Mendes, MNRJ, det. J. Gouvêa (MNRJ 31,484).

***Icriomastax* *phaeton* (Alexander, 1945)**: 1♂ (slide), Brazil, São Paulo, Serra da Cantareira, 900m, #7675, XII/1940, Col. L. Travassos, NMNH, det. C. P. Alexander (USNMENT 01222699 [holotype]); 1♂ (ethanol), Brazil, Rio de Janeiro, Cachoeiras de Macacu, Regua, Riacho, 19-26/V/2017, Col. Mermudes, Roza, Campello, Vaz, MNRJ, det. J. Gouvêa (MNRJ 30,660) (lost in the 2018 fire in National Museum).

***Icriomastax* *zikani* (Alexander, 1936)**: 1? (pinned), Brazil, Campo Belo, XI/22/1933, Col. Zikán, NMNH, det. C. P. Alexander [holotype]; 1? (pinned), Brazil, Campo Belo, III/31/1929, Col. Zikán, NMNH, det. C. P. Alexander [paratype]; 1? (pinned), Brazil, Campo Belo, XI/17/1935, Col. Zikán, NMNH, det. C. P. Alexander (USNMENT 01240524); 1♀, (slide), Brazil, Campo Belo, 700-800m, #5935, XI/22/1933, Col. Zikán, NMNH, det. C. P. Alexander (USNMENT 01222592 [holotype]); 1♀ (slide), Brazil, Campo Belo, #5935, III/31/1929, Col. Zikán, NMNH, det. C. P. Alexander (USNMENT 01222581 [paratype]); 1♂ (slide), Brazil, Campo Belo, #5935, XI/17/1935, Col. Zikán, NMNH, det. C. P. Alexander (USNMENT 01222710); 1♂ (ethanol), Brazil, Rio de Janeiro, Itatiaia, Casa do Pesquisador (Leo’s House), 22°27’14,9’’S 44°36’27,8’’W, 821m, 4-6/IV/2016, Col. A. P. Pinto, MNRJ, det. J. Gouvêa (MNRJ 30,565) (lost in the 2018 fire in National Museum); 1♂ (ethanol), Brazil, Rio de Janeiro, Itatiaia, Casa do Pesquisador (Leo’s House), 22°27’14,9’’S 44°36’27,8’’W, 821m, 4-6/IV/2016, Col. L. H. Gil-Azevedo, MNRJ, det. J. Gouvêa (MNRJ 30,566) (lost in the 2018 fire in National Museum); 1♀ (ethanol), Brazil, Rio de Janeiro, Angra dos Reis, Posto de Policiamento Florestal e do Meio Ambiente da Ilha Grande, UPAm-Joatinga, 23°8’23’’S 44°9’59’’W, 25/XI/2015, Col. L. Gouvêa, MNRJ, det. J. Gouvêa (MNRJ 30,608) (lost in the 2018 fire in National Museum); 1♀ (ethanol), Brazil, Rio de Janeiro, Itatiaia, PNI, Trilha para o Lago Azul, 821m, 1/X/2015, Col. L. H. Gil-Azevedo, MNRJ, det. J. Gouvêa (MNRJ 30,629) (lost in the 2018 fire in National Museum); 1♀ (ethanol), Brazil, Rio de Janeiro, Itatiaia, Casa do Pesquisador (Leo’s House), 22°27’16,29’’S 44°36’29,23’’W, 806m, 2/II/2016, Col. A. P. Pinto, MNRJ, det. J. Gouvêa (MNRJ 30,643) (lost in the 2018 fire in National Museum); 1♀ (ethanol), Brazil, Rio de Janeiro, Angra dos Reis, Posto de Policiamento Florestal e do Meio Ambiente da Ilha Grande, UPAm-Joatinga, 23°8’23’’S 44°9’59’’W, 25/XI/2015, Col. L. Gouvêa, MNRJ, det. J. Gouvêa (MNRJ 30,710) (lost in the 2018 fire in National Museum); 1♀ (ethanol), Brazil, Rio de Janeiro, Angra dos Reis, Posto de Policiamento Florestal e do Meio Ambiente da Ilha Grande, UPAm-Joatinga, 23°8’23’’S 44°9’59’’W, 25/XI/2015, Col. L. Gouvêa, MNRJ, det. J. Gouvêa (MNRJ 30,720) (lost in the 2018 fire in National Museum); 1♀ (ethanol), Brazil, Rio de Janeiro, Teresópolis, PNSO, Estrada da Barragem, 22°27’17,1’’S 42°59’50,0’’W 1134m, 26/IX/2017, Col. L. H. Gil-Azevedo, MNRJ, det. J. Gouvêa (MNRJ 30,722) (lost in the 2018 fire in National Museum); 1♀ (ethanol), Brazil, Rio de Janeiro, Petrópolis, Correas, Abrigo de Montanha do Dani, 13/XII/2017, Col. L. H. Gil-Azevedo, MNRJ, det. J. Gouvêa (MNRJ 30,824) (lost in the 2018 fire in National Museum).
