# Supplementary material for: A new perspective on Icriomastax (Diptera: Tipulidae): phylogeny and description of five new species
Source: PeerJ. 2026 Apr 16;14:e21121. doi: 10.7717/peerj.21121 (PMC13092232; doi:10.7717/peerj.21121)
Supplement: Supplemental Information 2 — Polymorphisms are represented by the letter A (0+1), B (0+2), C (0+3), D (1+2), E (1+3), F (2+3); G (0+1+2), H (0+1+3). [file peerj-14-21121-s002.docx]

**Appendix 2.** Data Matrix for the cladistics analyses for *Icriomastax* (“-” codes for inapplicable data; “?” for unavailable). Polymorphisms are represented by the letter A (0+1), B (0+2), C (0+3), D (1+2), E (1+3), F (2+3); G (0+1+2), H (0+1+3).

**1 2 3 4 5 6 7 8 9 10 11 12 13 14 15**

**123456789012345678901234567890123456789012345678901234567890123456789012345678901234567890123456789012345678901234567890123456789012345678901234567890123**

*T*.(*Tipula*)*oleracea* 2100101011210000110-001100-000-01-00-1001--11--01001100000-0-000000000-0-01100-0-000101-?01-0-0-00-00--31--0100111100010000---0-0????????????10--10-0200-

*Holorusia hespera* 2101A010101010001011001110-101100011101100A11--01101010000-0-000100000-0-001110111000120000-0-11-0-0201-0010100000000-10000---0-0050--------000--00-0200-

*Holorusia laticellula* 210010111??00010110-000110-10111011110???--01--11100110100-0-000100000-0-0001111110?012100110-??????21--00?0?????????????????????????????????1100????????

*Holorusia makara* 2101101010?011000111001110-101111-1110011--01--11101110000-0-000100000-0-0011101010?012101110-??????200-001000011011--00000---0-003-????????01100????????

*Holorusia mara* 21010-1110?011001111011000-101110111100110101--11100110100-0-000100000-0-0011101110?012100110-??????21--000000011011--10000---0-0???????????01100????????

*Holorusia similis* 21010-1110100010110-001010-101110111100100101--11100110100-0-000000000-0-001111111000121010-1-1010-0200-00000101101???0???????0-?????????????110010-1010-

*Holorusia siva* 21010-1A10?000101111101101000001001110111--01--11101010000-0-000100000-0-000111111000121001-1-1-10-01---1--001100011--10000---0-0???????????000--10-1010-

*Holorusia viettei* 1100101110?00100110-001000-101A10A1110110--00G111111110100-0-000100000-0-0001101011?0121001-0-??????21--000011000011--10000---0-0???????????100--????????

*Holorusia vinsoniana* 1110101110?00000110-001000-101100A1110010--00G111110110100-0-000100000-0-0011101010?0121001-0-??????21--000011100011--10000---0-00??????????000--????????

*I*. (*Neot*.) *maya* 20--0-111????1001111111100-101111-10-0111--01--11101110100-11000100000-0-00110-0-1?101200?????0101-1?????????????????????????????????????????????00-1210-

*I*. (*Neot*.) *paprzyckii* 20--0-111?0010101111111110-101110110-0111--01--11101010100-0-000000000-0-00110-0-1?101200?????1000-0?????????????????????????????????????????????01010111

*I*. (*Neot*.) *pectinella* 20--0-111????010110-110110-101111-10-0111--01--11101110100-11000100000-0-00110-0-11?0120000-0-??????1---1--0??10001??????????????????????????1101????????

*I*. (*Neot*.) *penata* 20--0-111?200110110-1101111101010210-0110--1A0111101110100-0100111111111110010-00111112000010-1-00-01---1--0?00000002101000---10?????????????10000101010-

*Z.diducta* 10--0-1111200011000-0011010011101-00-0011--01--11100111100-0-000100000-0-01101111010000-10A10-0--0-021--00010?01111010000112110-1???????????000--0100010-

*Z. flavicornis* 00--0-111?200010110-0011110111101-01110010111--11100111100-0-000100000-0-01101111010000-101-??10-0-021--00010100001000000112110-0?4-001010--000--0100110-

*Z. furcifera* 00--100110?00011001A001010-000-11-10-0011--01--11000111101011110100000-0-0110111211?000-10???????????????????????????????????????????????????????????????

*Z. novarae* 21000-1000200010110-010110-011210A10-00000111--0A001011110-11110100000-1101100-11011001-101-0-1000-021--00000010031010000012110-22500011----100--20-0110-

*Z. otagana* 1100101000?00011100-010110-010-10011200100100G001000011100-0-000100000-0-01100-1001?101-10110-??????21--0000??11100??????????????????????????????????????

*Z. perstrangalia*  10--10010?1000101010000000-000-11-10-0011--01--1-00011110101D110100000-0-01100-121?1000-1001??1-00-0?????????????????????????????????????????????20-0110-

*Icriomastax antinympha* 21100-0010100010110-010100-101010A11D00101001--1-000100001312111111110-0-10100-11000001-01--0-0--0-00--2021001000000120001100111?????????????111010-1010-

*Icriomastax calliope* 01100-01001000111111010110-101011-11200100101--1-000100001E1D111111110-1110101110000001-000-0-01-0--0--10121000000001200000---11?????????????????01000011

*Icriomastax catia* 11100-1110100000110-011110-10101001120010--01--1101?10001131111101111101D10101110000101-000-1-00-0-00--10---01???????????????????????????????010010-0200-

*Icriomastax coscaroni* 11110-1110?00011110-011110-101010011101100?11--0000?10001131D11111111111G1010111000?001-00011-??????0--21---010??????????????????????????????0110????????

**Appendix 2 (Cont.).** Data Matrix for the cladistics analyses for *Icriomastax* (“-” codes for inapplicable data; “?” for unavailable). Polymorphisms are represented by the letter A (0+1), B (0+2), C (0+3), D (1+2), E (1+3), F (2+3); G (0+1+2), H (0+1+3).

**1 2 3 4 5 6 7 8 9 10 11 12 13 14 15**

**123456789012345678901234567890123456789012345678901234567890123456789012345678901234567890123456789012345678901234567890123456789012345678901234567890123**

*Icriomastax craigi* 1100??1110?00111110-011110-101010111201100100G01100?110011E1A111111111A1G1010111100?001-000-01??????0--21--001???????????????????????????????0100????????

*Icriomastax euterpe* 01100-110????1?11111010110-101010011000100101--110??100101E1D111011110-110010111000?011-01A-0-??????1---1--01100000012000110000-??50011011--00110????????

*Icriomastax helios* 11110-0100100010110-000110-101010011200100111--0-001110001E1D111111111A1G10101110000111-00A-0-00-0-00--101211100000012000110000-01??????????001100100000-

*Icriomastax lopesae* 11100-1110?00010110-011110-101010211D01100100D01000?111011C1G111111111A1D1010111100?001-000-0-??????1---1---01???????????????????????????????0100????????

*Icriomastax monnei* 1111??1110?00111110-010110-101010011D01100100G111001110011H1G111111111A1G1010111100?101-000-0-??????0--21---010??????????????????????????????0110????????

*Icriomastax nebulipennata* ??????0??0?0011?????????????????????????????????1001000011F1111111111101D1000111101?101-0???????????0--10---0111101012000010000-?????????????0110????????

*Icriomastax nitra* 01100-0100?001110111011110-101010011100100101--11001100101E11111011110-110010111101?101-00A-0-??????0--00110100000001100000---11015001100---01-00????????

*Icriomastax nudicornis* 11100-1000100000110-011110-10100000121000--11--00000100001E12111011110-1100101110000101-00A-010A-0-00--101200110001012000010000-012-00100---0010000-1010-

*Icriomastax ocellata* 211A0-111000000011110101101101010011101010111--0-001100001E1D111011111A1G11101110000001-00111-0--0-00--1012001000000100000100010????00100---?????10-0000-

*Icriomastax phaeton* 01100-0100?000?01111000100-101001-111001001A1--1-000100001E1D11101111111D0010111001?001-000-0-??????B1-10100?1111000210001101211?????????????????????????

*Icriomastax zikani* 11110-010?1000101111011110-101010011201100101--10000100001311111011110-1110001111000001-000-0-???????????????100000012000010000-?????????????0110????????

*I*. (*Isch*.) *araucana* 21010-1010101011010-010110-000-00011101100101--00000011110-0-111111110-1111101010000101-000-0-1A-11021--00001111001010001-0---11?150001011--?????10-0000-

*I*. (*Isch*.) *concinna* 011110101010101A110-000000-011B10010-10100111--0-00111001131211111111111D11A00-10011101-01--0-11-0-021--001011000001--00000---110?51001011--0????10-0200-

*I*. (*Isch*.) *decorata* 21110-1010101010110-010100-010-00011110100111--0-001111100-0-11111111111111101010000A01-00-00-11-1-121--00001100001010001-100011005100100---000--10-0010-

*I*. (*Isch*.) *delpontei* 1110101010101010010-0001110010-00010-00100101--0-001110111F12111111110-1D11100-10011101-01--1111-1-121--00001100000010001-0---110????????????00--10-0200-

*I*. (*Isch*.) *eburnea* 1101101011?0101011110101111011110011110100101--01001010111E1D111011110-1G11101110000001-001-0010-1-021--0000110000000001000---0-?????????????00--0101210-

*I*. (*Isch*.) *episema* 110010101?110000010-0100000000-11-1110011--01--1????000000-0-111011110-0-11100-100?1001-0?????0--0-0?????????????????????????????????????????????01002011

*I*. (*Isch*.) *fagetorum* 2110111010?01001100-010100-000-00000-11010111--0-001110110-0-111111000-111100101000?101-01101-??????200-001010000001--01010---10?????????????00--????????

*I. (Isch.)* *fuscostigmosa* 21010-1010100000010-0101011000-00000-11000111--00001110100-0-111100000-1111101010000101-000-1-??????2?1?00001011101000001-0---0-?????????????00--????????

*I. (Isch.)* *goldfinchi* 1100111011?010?111110111110011010011110000101--00001110010-0-110000000-0-01100-100?0101-0?????0--1-121--?????????????????????????????????????00--00-0000-

*I. (Isch.)* *larotypa* 21110-101010001001110101011010-00010-10100111--0-001011110-1211111111101G11101010000001-000-1-11-1-021--0000100000000100010---111???????????000--0100200-

*I. (Isch.)* *par* 110110101010111011110101111011010011D1100010A0000001010110-0-111100000-1101100-10000A01-00110-00-10021--1--0010000000000000---1100??001010--000--????????

**Appendix 2 (Cont.).** Data Matrix for the cladistics analyses for *Icriomastax* (“-” codes for inapplicable data; “?” for unavailable). Polymorphisms are represented by the letter A (0+1), B (0+2), C (0+3), D (1+2), E (1+3), F (2+3); G (0+1+2), H (0+1+3).

**1 2 3 4 5 6 7 8 9 10 11 12 13 14 15**

**123456789012345678901234567890123456789012345678901234567890123456789012345678901234567890123456789012345678901234567890123456789012345678901234567890123**

*I. (Isch.)* *penai* 01000-1010?011?0000-001000-001010000-0010--11--0-001110110-0-000100000-0-01100-1001?101-01--0-??????1---1--0110000001000000---0-1???????????000--????????

*I. (Isch.)* *peracuta* 21101A10002011101111000110-010-01-00-10010111--0000111011131211111111101G11001011000001-001-1-10-110200-001011000001--01010---10??5100100---000--10-0000-

*I. (Isch.)* *porteri* 21000-1010100010110-010100-000-00000-10000111--0-001010000-0-000100000-0-0110110-0A0101-00110-1101-121--000001001011--00000---0-0?51001011--000--00-0000-

*I. (Isch.)* *prionoceroides* 11100-101?10100101100001011011G1001110010--01--0-001000000-0-000000000-0-01100-0-0?1101-0?????00-0-0?????????????????????????????????????????????00-0000-

*I. (Isch.)* *problematica* 01000-100?1010?0100-000010-000-11-10-0010--0AB01-000010100-0-000100000-0-01100-0-0?1101-0?????1A-0-0?????????????????????????????????????????????00-0000-

*I. (Isch.)* *rubriventris* 1110101010?0110111110111111010-1001110110010A0000001000100-0-000000000-0-01100-1000?111-00-1001--1-021--1--011000001--01010---11?????????????00--10-0000-

*I. (Isch.)* *rubroabdominalis* 0110101010?0100111110101111011010010-00100101--0-001000100-0-000000000-0-01100-10000101-01--0-00-100200-1--011000001--01000---11?????????????00--10-0010-

*I*. (*Isch*.) *rufistigmosa* 11000-101?100000000-0101011000-00000-11000011--0-000011100-0-111011100-10111011110?0011-0?????0--111?????????????????????????????????????????????10-0010-

*I*. (*Isch*.) *schineriana* 1110101010100011110-010100-011200010-00100100G01000101010131211111111111211100-11011101-01011-0--0-021--0000110000002001010---100???001011--?00--10-0000-

*I*. (*Isch*.) *scutellumnigra* 0100101010?101?0000-0011011011210011100100101--00001000000-0-000000000-0-01100-10010001-001-0-??????201-1--0110100000000010---11??5000100---?00--????????

*I*. (*Isch*.) *shannoniana* 1100101010100100110-0111111000-01-00-10010111--0-00111010131211111111111111100111011101-01--0-0--0-0201-0000110000001001010---10????001011--?00--00-0000-

*I*. (*Isch*.) *silvai* 21001A1010?00010100-010100-001010010-0110--01--1-001010000-0-000000000-0-01101110000011-01--0-0--0-0201-0010110000001000000---10?????????????00--00-0200-

*I*. (*Isch*.) *skuseana* 01000-1000101000110-010000-000-01-00-11000011--0000100111111111101111111111101010011001-00011-01-1-021--1--0010000001100000---111????????????00--1100000-

*I*. (*Isch*.) *rufiventris* 01000-101?110000010-000100-000-10010-01100101--11001000000-0-000000000-0-01100-100?1A01-0?????1A-0-021--1--001000011--00000---100???????????000--10-0010-

*I*. (*Isch*.) *terminata* 00--0-101?101000010-000000-001010010-0010--01--0-001110000-0-000000000-0-01100-0-0?0001-0?????00-0-0?????????????????????????????????????????????10-0210-

*I*. (*Isch*.) *trunculata* 211110101010?000100-010100-000-01-00-10010111--0-001110100-0-11111111111D11001111000101-001-0-10010121--001011000001--01010---10?????????????110010-0000-
