# Supplementary material for: A new perspective on Icriomastax (Diptera: Tipulidae): phylogeny and description of five new species
Source: PeerJ. 2026 Apr 16;14:e21121. doi: 10.7717/peerj.21121 (PMC13092232; doi:10.7717/peerj.21121)
Supplement: Supplemental Information 3 [file peerj-14-21121-s003.docx]

Appendix 3: Characters list

1. Body length, size:

(0) small, up to 15mm;

(1) medium, between 16mm and 22mm;

(2) large, over 23mm.

2. Head, vertex, vertical tubercle:

(0) absent;

(1) present.

3. Head, vertex, vertical tubercle, shape:

(0) one lobe;

(1) with two lobes.

4. Head, vertex, vertical tubercle, height:

(0) low, smoothly projected off the head;

(1) high strongly projected off the head.

5. Head, occiput, color:

(0) homogeneous

(1) with spots.

6. Head, occiput, spots, shape:

(0) stripe;

(1) circular.

7. Head, rostrum, length:

(0) shorter than head;

(1) subequal or longer than head.

8. Head, rostrum, lateral region, stripes:

(0) absent;

(1) present.

9. Head, rostrum, nasus, length:

(0) short, not exceeding rostrum length;

(1) long, exceeding rostrum length.

10. Head, antenna (male), flagellomeres, number:

(0) ten

(1) eleven

11. Head, antenna (female), flagellomeres, number:

(0) 9

(1) 10

(2) 11

12. Head, antenna, flagellomeres, width:

(0) segments gradually thinner towards the apex, becoming progressively narrower from first to terminal segment;

(1) last segment abruptly thinner/smaller towards the apex.

13. Head, antenna, flagellomeres, shape:

(0) filiform

(1) serrate

14. Head, palpus, color:

(0) homogeneous;

(1) at least one segment with proximal and/or distal tip lighter.

15. Head, palpus, segment V, length:

(0) shorter than the segments I-IV combined.

(1) subequal or longer than the segments I-IV combined;

16. Thorax, prescutum, color pattern:

(0) lateral and central stripes with same color;

(1) lateral stripes darker than the central stripes;

17. Thorax, prescutum, stripes, borders:

(0) absent;

(1) present.

18. Thorax, scutum, upper and inner spot:

(0) absent;

(1) present.

19. Thorax, scutum, darker margin:

(0) absent;

(1) present.

20. Thorax, scutum, darker margin, position:

(0) proximal margin, near pleura;

(1) distal margin, near scutellum.

21. Thorax, scutum, distal margin, horizontal band:

(0) absent;

(1) present.

22. Thorax, scutellum, middle stripe:

(0) absent;

(1) present.

23. Thorax, scutellum, distal margin, shape:

(0) rounded;

(1) squared.

24. Thorax, mediotergite, middle stripe:

(0) absent;

(1) present.

25. Thorax, mediotergite, lateral spots:

(0) absent;

(1) present.

26. Thorax, mediotergite, inferior margin, spots:

(0) absent;

(1) present.

27. Thorax, mediotergite, inferior margin, spots, color:

(0) white to yellow;

(1) brown to black.

28. Thorax, pleura, dark stripe from cervical sclerites to base of wing:

(0) absent;

(1) present.

29. Thorax, pleura, between prescutum and anepisternum, spot:

(0) absent;

(1) present.

30. Thorax, anepisternum, color pattern:

(0) homogeneous;

(1) with spot.

31. Thorax, anepisternum, spot, position:

(0) middle;

(1) near tergum;

(2) near katepisternum.

32. Thorax, anepisternum, background color:

(0) brown to black;

(1) white to yellow.

33. Thorax, katepisternum, color pattern:

(0) with spot;

(1) homogeneous.

34. Thorax, katepisternum, spot, position:

(0) distal margin, between coxa I and II;

(1) proximal margin, near aneposternum.

(2) middle.

35. Thorax, katepisternum, background color:

(0) brown to black;

(1) white to yellow.

36. Thorax, anepimeron, color pattern:

(0) homogeneous;

(1) with spot.

37. Thorax, anepimeron, spot, position:

(0) near katepimeron;

(1) near tergum;

(2) middle.

38. Thorax, katepimeron, background color:

(0) white to yellow;

(1) brown to black.

39. Thorax, meron, inferior margin, shape:

(0) rounded;

(1) square.

40. Thorax, meron, background color:

(0) brown to black;

(1) white to yellow.

41. Thorax, meron, color:

(0) with spot;

(1) homogeneous.

42. Thorax, anatergite, color:

(0) brown to black;

(1) white to yellow.

43. Thorax, katatergite, color:

(0) brown to black;

(1) white to yellow.

44. Coxa, background color:

(0) white to yellow;

(1) brown to black.

45. Coxa, color:

(0) with spot;

(1) homogeneous.

46. Coxa, spot, distribution:

(0) fore;

(1) mid;

(2) hind.

47. Coxa, spot, position:

(0) proximal margin

(1) center

48. Trochanter, background color:

(0) brown to black;

(1) yellow.

49. Femora, terminal ring:

(0) absent;

(1) present.

50. Femora, femoral ctenidium:

(0) absent;

(1) present.

51. Tibia, base near femur:

(0) homogeneous, same color as tibia length.

(1) whitish yellow, lighter than tibia length;

52. Tibia, spur:

(0) absent;

(1) present.

53. Wing, cell C, color:

(0) hyaline.

(1) brown;

54. Wing, stigma, whitened streak:

(0) absent;

(1) present.

55. Wing, vein Rs, origin, spot:

(0) absent;

(1) present.

56. Wing, vein m-cu, near fork, spot:

(0) absent;

(1) present.

57. Wing, Rs cell, color:

(0) homogeneous;

(1) with a spot.

58. Wing, br cell, color:

(0) homogeneous;

(1) with a spot.

59. Wing, br cell, spots, distribution:

(0) following R;

(1) center of cell;

(2) following Rs;

(3) distal portion of the cell, near r-r, r-m and discal cell.

60. Wing, bm cell, color:

(0) homogeneous;

(1) with a spot.

61. Wing, bm cell, spots, distribution:

(0) following vein M;

(1) mid cell;

(2) distal portion of the cell, near m-cu and/or discal cell

62. Wing, r3 cell, color:

(0) homogeneous;

(1) with a spot.

63. Wing, r4 cell, color:

(0) homogeneous;

(1) with a spot.

64. Wing, r5 cell, color:

(0) homogeneous;

(1) with a spot.

65. Wing, discal cell, color:

(0) homogeneous;

(1) with whitened streak.

66. Wing, m1 cell, color:

(0) homogeneous;

(1) with a spot.

67. Wing, m2 cell, color:

(0) homogeneous;

(1) with a spot.

68. Wing, m3 cell, color:

(0) homogeneous;

(1) with a spot.

69. Wing, m4 cell, color:

(0) homogeneous;

(1) with a spot.

70. Wing, cua cell, color:

(0) homogeneous;

(1) with a spot.

71. Wing, cua cell, spots, position:

(0) cell base

(1) following vein CuA

72. Wing, cup cell, color:

(0) homogeneous;

(1) with a spot.

73. Wing, cup cell, spots, position:

(0) near the cell base;

(1) middle of the cell;

(2) margin of the wing;

74. Wing, anal lobe, color:

(0) homogeneous;

(1) with a spot.

75. Wing, vein Rs, length:

(0) shorter to subequal to m-cu

(1) longer than m-cu

76. Wing, vein Rs, base, shape:

(0) straight;

(1) curved.

77. Wing, vein r, position:

(0) joining R3;

(1) joining R3+4.

78. Wing, vein R4, shape:

(0) straight;

(1) sinuous at midlength.

79. Wing,vein R4, shape, sinuous at midlength:

(0) strongly sinuous;

(1) weakly sinuous.

80. Wing, vein R5, middle length:

(0) parallel to vein R4;

(1) curved towards vein R4.

81. Wing, vein R5, middle length, curved to vein R4:

(0) very smooth

(1) intermediate

(2) strong

82. Wing, vein M, before branching:

(0) straight;

(1) arched.

83. Wing, vein M1+2 (male), length:

(0) shorter to subequal to m-m;

(1) longer than m-m.

84. Wing, vein M1+2 (female), length:

(0) shorter to subequal to m-m;

(1) longer than m-m.

85. Wing, vein M4, origin, originated from discal cell:

(0) separate from vein m-cu;

(1) at same point as m-cu.

86. Wing, cua cell, width at wing margin:

(0) Narrow at wing margin, less the extent of M1-M3

(1) Unusually wide at wing margin, nearly equaling the extent of M1-M3

87. Wing, anal lobe, development:

(0) undeveloped;

(1) developed;

(2) very developed.

88. Wing, anal lobe, development, very development, margin:

(0) curved;

(1) straight.

89. Wing, squama:

(0) bare;

(1) not bare.

90. Abdomen (male), tergites:

(0) with spots;

(1) homogeneous.

91. Abdomen (male), tergite, lateral margin:

(0) darker than center;

(1) lighter than center.

92. Abdomen (male), tergite, posterior margin:

(0) light;

(1) dark.

93. Abdomen (male), sternites, color:

(0) all sternites with similar colors / pattern;

(1) gradually getting darker towards hypopygium;

(2) the first and last sternites darker.

94. Abdomen (male), sternites, lateral margin, color:

(0) lighter than center;

(1) darker than center.

95. Abdomen (female), tergites, color:

(0) homogeneous, same shade from anterior to posterior margin;

(1) different shades from anterior to posterior margin.

96. Abdomen (female), tergites, lateral margin, color:

(0) darker than center;

(1) lighter than center.

97. Abdomen (female), tergites, dorsal margin, color:

(0) dark line;

(1) light line.

98. Abdomen (female), sternites, color:

(0) homogeneous, same shade from anterior to posterior margin;

(1) different shades from anterior to posterior margin.

99. Abdomen (female), sternites, color, lateral margin:

(0) lighter than center;

(1) darker than center.

100. Abdomen (female), sternites, middle line:

(0) absent;

(1) present.

101. Hypopygium, tergite IX, posterior margin, shape:

(0) produced outwardly;

(1) linear;

(2) emarginated.

102. Hypopygium, tergite IX, posterior margin, shape of emargination:

(0) V-shaped;

(1) U-shaped.

103. Hypopygium, tergite IX, posterior margin, shape, emarginated, V-shaped:

(0) wide V-shape;

(1) deep V-shape.

104. Hypopygium, tergite IX, posterior margin, shape, produced outwardly:

(0) obtuse;

(1) truncate;

(2) acuminate;

(3) sinuous (U-shape).

105. Hypopygium, tergite IX, posterolateral lobes:

(0) present;

(1) absent.

106. Hypopygium, tergite IX, posterolateral lobes, number:

(0) 1+1

(1) one

(2) three

107. Hypopygium, tergite IX, posterolateral lobes, shape:

(0) round

(1) triangular

(2) straight to smootly sinuous

(3) bifurcate

108. Hypopygium, tergite IX, spineous setae, on posterolateral lobes:

(0) absent;

(1) present.

109. Hypopygium, gonocoxite, length:

(0) longer than tergite IX;

(1) about same length as tergite IX.

110. Hypopygium, gonocoxite, length:

(0) same width throught lenght;

(1) apex narrower

(2) midlenght wider

111. Hypopygium, lobe of gonostylus, width along the length:

(0) similar width;

(1) with width variation.

112. Hypopygium, lobe of gonostylus, ventral margin:

(0) straight;

(1) sinuous (concave/convex).

113. Hypopygium, lobe of gonostylus, dorsal margin:

(0) straight;

(1) sinuous (concave/convex).

114. Hypopygium, lobe of gonostylus, posterior margin, shape:

(0) round or narrowly round;

(1) truncate;

(2) serrated;

(3) pointed.

115. Hypopygium, lobe of gonostylus, setae abundance:

(0) havily distributed;

(1) weakly destributed.

116. Hypopygium, clasper of gonostylus, shape:

(0) variable width throught length;

(1) similar width throught length.

117. Hypopygium, clasper of gonostylus, shape, variable width, apex:

(0) narrower than midlength;

(1) apex wider than midlength, oupassing the direction of midlenght;

(2) similar width than midlength.

118. Hypopygium, clasper of gonostylus, shape, variable width, base:

(0) similar width as midlength;

(1) wider than midlenght;

(2) narrower.

119. Hypopygium, clasper of gonostylus, posterodorsal margin:

(0) no width variation;

(1) expanded.

120. Hypopygium, clasper of gonostylus, posterior margin:

(0) no width variation;

(1) expanded ventrally.

121. Hypopygium, clasper of gonostylus, setae:

(0) present;

(1) absent.

122. Hypopygium, clasper of gonostylus, setae abundance:

(0) weakly destributed;

(1) havily distributed.

123. Hypopygium, clasper of gonostylus, spines:

(0) absent;

(1) present.

124. Hypopygium, clasper of gonostylus, spines, distribution:

(0) outer margin;

(1) intire inner face, from base to outer margin;

(2) inner face near apex.

125. Hypopygium, clasper of gonostylus, spines, number:

(0) less than 50;

(1) over 50.

126. Hypopygium, clasper of gonostylus, spines, shape:

(0) triangular;

(1) round;

(2) long and pointed.

127. Hypopygium, clasper of gonostylus and lobe of gonostylus, fusion:

(0) absent;

(1) present.

128. Hypopygium, clasper of gonostylus and lobe of gonostylus, fusion extension:

(0) Extensive fused, from base to midlength;

(1) Not extensive fused, only near base.

129. Hypopygium, sperm pump, shape:

(0) round, as wide as long;

(1) oval, wider than long;

(2) pear shape.

130. Hypopygium, compressor apodeme, width:

(0) wider than longer;

(1) longer than wider;

(2) long as wide.

131. Hypopygium, aedeagal guide, posterior margin, shape:

(0) leaf-like;

(1) truncate;

(2) round to weakly produced;

(3) arrow;

(4) bifid;

(5) long and narrow, finger-like.

132. Hypopygium, aedeagal guide, posterior margin, finger-like:

(0) getting thinner into a triangular shape;

(1) same width through length.

133. Hypopygium, aedeagal guide, lateral expansions, shape:

(0) round;

(1) triangular.

134. Hypopygium, aedeagal guide, lateral expansions, shape, round, stem:

(0) absent;

(1) present.

135. Hypopygium, aedeagal guide, lateral expansions, shape, round, heigth:

(0) weakly projected;

(1) strongly projected.

136. Hypopygium, aedeagal guide, lateral expansions, shape, round, posterior margin, strongly sinuos, almost bifurcate:

(0) absent;

(1) present.

137. Hypopygium, aedeagal guide, lateral expansions, shape, round, width:

(0) same width through length;

(1) width variation through length.

138. Hypopygium, aedeagal guide, lateral expansions, shape, round, width, variation through length:

(0) long as wide;

(1) longer than wider.

139. Hypopygium, aedeagal guide, lateral expansions, shape, triangular, apex:

(0) round;

(1) pointed.

140. Hypopygium, aedeagal guide, lateral expansions,shape, triangular, apex, pointed:

(0) convergent;

(1) divergent.

141. Hypopygium, aedeagus, diameter:

(0) getting thinner;

(1) thick through length.

142. Abdomen (male), sternite VIII, posterior margin, shape:

(0) linear;

(1) U-shape.

143. Abdomen (male), sternite VIII, posterior margin, posterolateral projections:

(0) absent;

(1) present.

144. Abdomen (male), sternite VIII, posterior margin, posterolateral projections, origin:

(0) posterior margin;

(1) center of sternite.

145. Abdomen (male), sternite VIII, posterior margin, posterolateral projections, length:

(0) low, just exceeding the margin of the sternite;;

(1) exceeding the entire male terminalia.

146. Ovopositor, cercus, shape:

(0) ventrally expanded at base;

(1) slender, same width through length;

(2) interly broad.

147. Ovopositor, cercus, setae:

(0) absent;

(1) present.

148. Ovopositor, cercus, setae, lenght:

(0) short, barely exceeding cercus margin;

(1) long, clearly exceeding cercus margin.

149. Ovopositor, hypogynial valve, width:

(0) stout;

(1) slender.

150. Ovopositor, hypogynial valve, lenght:

(0) reaching half of hypogynial valve;

(1) reaching one third or less of hypogynial valve;

(2) reaching two thirds or more of hypogynial valve;

151. Ovopositor, hypogynial valve, postero magin, shape:

(0) rounded;

(1) pointed/triangular;

(2) truncate.

152. Ovopositor, hypogynial valve, setae:

(0) absent;

(1) present.

153. Ovopositor, hypogynial valve, setae, abundace:

(0) very abundant;

(1) sparsely distributed.
