# Supplementary figures and images for: A new perspective on Icriomastax (Diptera: Tipulidae): phylogeny and description of five new species
Source: PeerJ. 2026 Apr 16;14:e21121. doi: 10.7717/peerj.21121 (PMC13092232; doi:10.7717/peerj.21121)

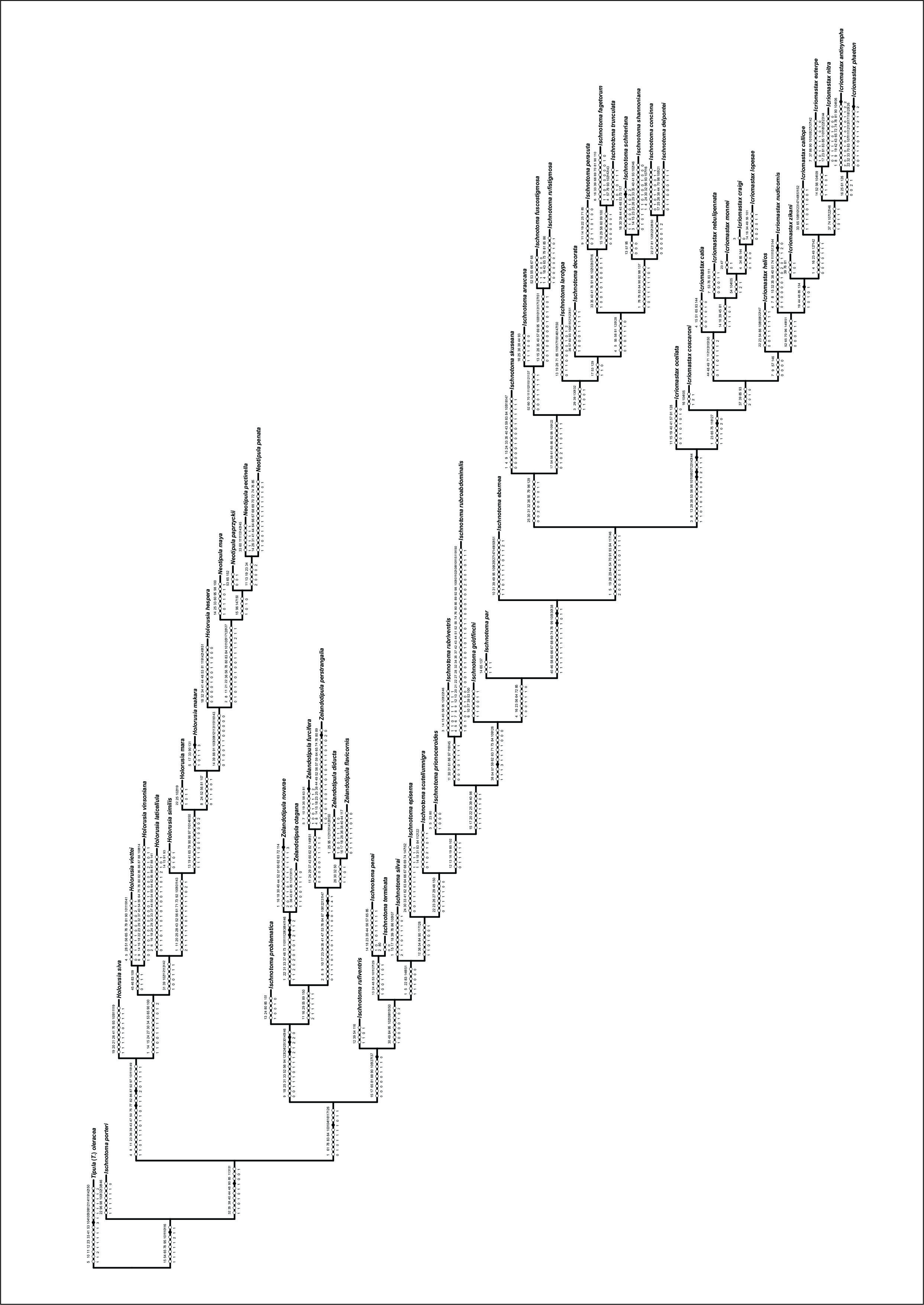

Supplement: Supplemental Information 4 — Circles represent apomorphies (white = homoplastic; black = non-homoplastic). Numbers above the circles indicate character numbers; numbers below indicate character states. Characters with ambiguous optimization were treated under ACCTRAN. [file peerj-14-21121-s004.jpg]
